# Long-term characteristics of exudative age-related macular degeneration in Japanese patients

**DOI:** 10.1371/journal.pone.0261320

**Published:** 2021-12-14

**Authors:** Masaaki Saito, Tomohiro Iida, Kuniharu Saito, Mariko Kano, Kanako Itagaki, Ichiro Maruko, Tetsuju Sekiryu

**Affiliations:** 1 Department of Ophthalmology, Fukushima Medical University School of Medicine, Fukushima, Japan; 2 Department of Ophthalmology, Hirosaki University Graduate School of Medicine, Hirosaki, Japan; 3 Department of Ophthalmology, Tokyo Women’s Medical University School of Medicine, Tokyo, Japan; Sung Kyun Kwan University School of Medicine at Samsung Medical Center Cancer Center, REPUBLIC OF KOREA

## Abstract

**Purpose:**

The present study aimed to evaluate the clinical characteristics of exudative age-related macular degeneration (AMD) in Japanese patients over a 10-year period and to compare the past our report.

**Methods:**

We retrospectively reviewed 1,600 treatment-naïve patients (1,777 eyes) with exudative AMD. The 10 years were divided into 2-year phases I to V.

**Results:**

Of the 1,600 patients, 720 (45.0%), 733 (45.8%), 98 (6.1%), and 49 (3.1%) were diagnosed with typical AMD, polypoidal choroidal vasculopathy (PCV), retinal angiomatous proliferation, and combined subtypes, respectively. The prevalence of PCV decreased from 54.7% in phase I to 46.0% at phase V. Of the 1,777 eyes, the mean baseline logarithm of the minimum angle of resolution best-corrected visual acuities (BCVAs) in phases I, II, III, IV, and V were 0.70, 0.66, 0.55, 0.50, and 0.48, respectively. Phases III, IV, and V had significantly (P = 0.0012, P<0.0001, P<0.0001, respectively) better baseline VAs compared with phase I. The mean lesion sizes in phases I, II, III, IV, and V were 8.6, 6.7, 5.3, 5.7, and 5.7 Macular Photocoagulation Study disc areas, respectively. The sizes were significantly (P<0.0001 for all comparisons) smaller in phases III, IV, and V compared with phase I.

**Conclusions:**

Although the prevalence of PCV decreased from 54.7% in phase I to 46.0% at phase V, PCV has nevertheless been highly prevalent in Japanese patients with AMD compared with Caucasian patients. The annual better baseline VAs and smaller lesion sizes over time might be related to development of treatment and better concerns about AMD.

## Introduction

Choroidal neovascularization (CNV) secondary to exudative age-related macular degeneration (AMD) is responsible for significant visual loss [1–3]. Anti-vascular endothelial growth factor (VEGF) therapy using ranibizumab (Lucentis, Genentech, Inc., South San Francisco, CA) or aflibercept (Eylea, Regeneron, Tarrytown, NY, and Bayer, Berlin, Germany) has become an evidence-based therapy for treating AMD worldwide after major clinical trials [4–6]. However, anti-VEGF drugs tend to cause different responses based on the CNV subtypes in clinical settings. Major studies have not reported the prevalence of polypoidal choroidal vasculopathy (PCV) because of the absence of indocyanine green angiography (ICGA) findings. Our previous study showed that PCV is highly prevalent in Japanese patients with AMD [7]. In addition, the high percentage of PCV is a major characteristic in Asian patients with AMD [8,9]. It is important to distinguish CNV subtypes correctly to achieve adequate treatment results.

Recently, a new anti-VEGF drug, brolucizumab (Beovu^®^, Novartis Pharma AG, Basel, Switzerland), was reported to be non-inferior to aflibercept in major clinical trials and became available for medical use in Japan in May 2020 [10,11]. Brolucizumab has been approved to use 12-week dosing schedule after three monthly loading doses, which can prevent frequent visits for AMD patients. However, intraocular inflammation (IOI) after injection of brolucizumab has been reported [12,13]. Therefore, it has been reconfirmed that the proper use of anti-VEGF drugs should be needed according to CNV subtypes.

The advances in treatments for patients with AMD such as photodynamic therapy (PDT) and anti-VEGF therapy can help general ophthalmologists when consulting with retina specialists about patients with early-stage AMD. As a result, the prevalence of early-stage AMD might increase along with the evolution of treatments for AMD. Therefore, the percentages of the CNV subtypes also might be changed or differ compared with past reports. However, the long-term characteristics of exudative AMD and the changes in the baseline visual acuity (VA) levels have not been evaluated.

The purpose of the current study was to evaluate the clinical characteristics of AMD in Japanese patients over a 10-year period and to compare the past our report.

## Methods

We retrospectively reviewed 1,600 treatment-naïve patients (1,199 men, 401 women; age range, 50–94 years; mean ± standard deviation, 74.3 ± 8.5 years) with exudative AMD for ten years from March 2003 to February 2013 at the Macula Services of Fukushima Medical University Hospital. The institutional review board/ethics committee at Fukushima Medical University approved the retrospective chart review study of AMD and the retrospective comparative analyses performed in this study. All patients provided written informed consent to have their medical records used in this research. All date was fully anonymized before accessed them. We classified the patients into three subtypes of neovascular AMD: PCV, retinal angiomatous proliferation (RAP), and typical AMD according to previous report in Phase I [7]. We also defined patients as having combined subtypes if, for example, one eye had PCV and the other eye had typical AMD [7,14]. A clinical diagnosis of PCV was established based on the findings of the polypoidal lesions on ICGA. The diagnosis of RAP and typical AMD was made based on fundus color photography, optical coherence tomography (OCT), fluorescein angiography (FA) and ICGA. The exclusion criteria were previous treatments for AMD such as laser photocoagulation, submacular surgery, or PDT; CNV secondary to other causes such as high myopia or angioid streaks; idiopathic submacular CNV; tears in the retinal pigment epithelium (RPE); history of phacoemulsification followed by IOL implantation within 3 months; and other maculopathies such as diabetic maculopathy, retinal vascular occlusion or idiopathic juxtafoveal retinal telangiectasis. We divided the 10-year period into five 2-year periods (phases I-V) with phase I extending from March 2003 to February 2005, phase II from March 2005 to February 2007, phase III from March 2007 to February 2009, phase IV from March 2009 to February 2011, and phase V from March 2011 to February 2013. We previously reported the clinical characteristics of AMD in phase I [7]. In Japan, PDT was approved in May 2004 and anti-VEGF drugs ranibizumab and aflibercept became available for medical use in March 2009 and December 2012, respectively.

We used the best-corrected VA (BCVA) measured with a Japanese standard decimal VA chart and converted that measurement to the logarithm of the minimum angle of resolution (logMAR) scale for analyses. We divided the VA levels as following: good, exceeding 20/40 (0.5 decimal VA); moderate, 20/200 (0.1 decimal VA) to 20/40 (0.5 decimal VA); and poor, below 20/200 (0.1 decimal VA). All patients underwent a standardized examination including slit-lamp biomicroscopy with a contact lens, fundus color photography, FA, ICGA with a fundus camera (TRC-50 FA/IA/ImageNet H1024 system, Topcon, Tokyo, Japan), and/or a confocal scanning laser ophthalmoscope (Heidelberg Retina Angiograph 2 [HRA2], Heidelberg Engineering, Heidelberg, Germany). High-speed ICGA using HRA2 was performed routinely from August 2005. All OCT examinations were performed using time-domain OCT (OCT 3000, Carl Zeiss Meditec, Dublin, CA, or OCT-Ophthalmoscope, Nidek-OTI, Gamagori, Japan) or spectral-domain OCT (3D-OCT, Topcon, Tokyo; Cirrus OCT, Carl Zeiss; or Heidelberg Spectralis OCT, Heidelberg Engineering).

Three retina specialists (KS, KI, and MK) evaluated all color fundus photographs and angiograms. A fourth reviewer (TI or MS) evaluated 52 (3.3%) of the 1,600 patients when the three reviewers were unable to classify the findings into one of the three subtypes. We measured the lesion sizes based on FA findings by ImageNet 2000 software (Topcon) in all eyes according to the Treatment of Age-Related Macular Degeneration with Photodynamic Therapy Study [15], i.e., the lesions were measured by FA including CNV, serous detachment of the RPE, blood, and an area of elevated blocked fluorescence corresponding to scar tissue or pigment. Two independent examiners (KI and MK) measured the lesion sizes using FA findings, and if the thicknesses between the two examiners differed by more than 15% of the mean of the two values, a third examiner (MS) made the decision. According to the criteria and results of lesion sizes based on major PDT studies [15–17], we divided the lesions into the following three groups: small, less than one disc area (DA); medium, one to nine DAs; and large, more than nine DAs. Other grayish-white subretinal fibrinous exudates were defined as highly homogenous reflective masses bridging the neurosensory retina and the RPE on OCT images.

Statistical analyses were performed using the Mann-Whitney *U*-test or chi-square test. *P* < 0.05 was considered significant.

## Results

### AMD subtypes

Of the 1,600 patients, 720 (45.0%), 733 (45.8%), and 98 (6.1%) patients were diagnosed with typical AMD, PCV, and RAP, respectively. The remaining 49 (3.1%) patients were diagnosed with combined subtypes, with both typical AMD in one eye and PCV in the other eye (Table 1). The raw data for the patient characteristics are presented in S1 Table.

**Table 1:** Characteristics of neovascular AMD in this study.

Characteristic	Typical AMD	PCV	Combined	RAP
No. patients (n = 1,600)	720	733	49	98
%	45.0	45.8	3.1	6.1
Gender				
Male	550	566	37	46
Female	170	167	12	52
Mean age (mean±SD)				
Total	74.6 ± 9.0	73.0 ± 7.8	75.6 ± 7.5	81.0 ± 7.8
Male	74.8 ± 8.7	73.2 ± 7.5	75.9 ± 7.0	81.2 ± 6.3
Female	73.9 ± 9.9	72.5 ± 8.6	74.8 ± 9.0	80.8 ± 9.0
No. eyes (n = 1,777)	757	798	98	124
%	42.6	44.9	5.5	7.0
LogMAR BCVA (Snellen equivalent)	0.62 (20/83)	0.50 (20/63)	0.62 (20/83)	0.64 (20/86)
Lesion size (DA)	5.6	7.0	NA	4.8

AMD, age-related macular degeneration; PCV, polypoidal choroidal vasculopathy; RAP, retinal angiomatous proliferation; SD, standard deviation; logMAR BCVA, logarithm of the minimum angle of resolution best-corrected visual acuity; DA, Macular Photocoagulation Study disc areas.

**Fig 1:**
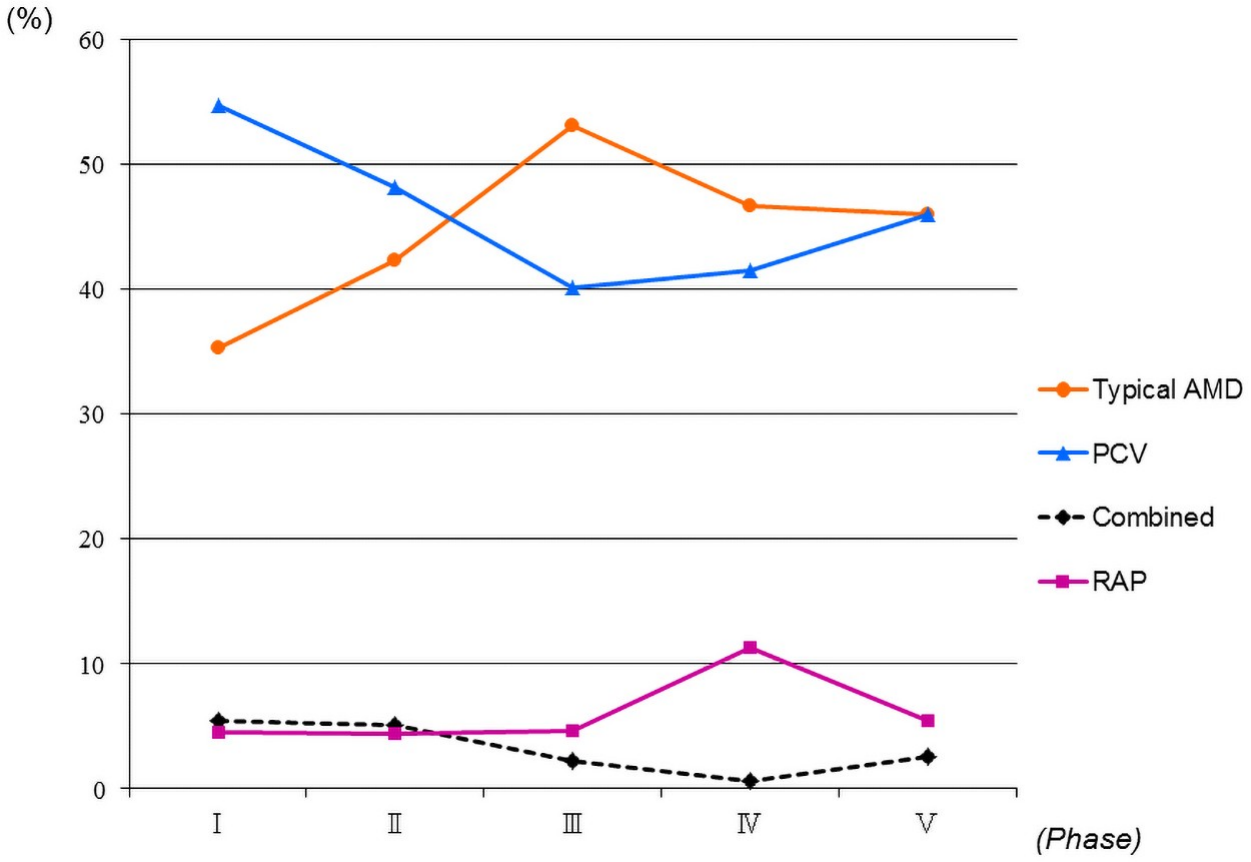
Changes in the prevalence rates of age-related macular degeneration (AMD) subtypes in 1,600 patients. Typical AMD increased from 35.3% in phase I to 46.0% in phase V and polypoidal choroidal vasculopathy (PCV) decreased from 54.7% in phase I to 46.0% in phase V. Phases I, II, III, IV, and V spanned, respectively, March 2003 to February 2005, March 2005 to February 2007, March 2007 to February 2009, March 2009 to February 2011 and March 2011 to February 2013. RAP = retinal angiomatous proliferation; combined = combined subtypes.

Table 2 shows the changes in the prevalence rates of the AMD subtypes by the phases. The prevalence of PCV decreased from 54.7% in phase I to 46.0% at phase V. However, the prevalence of typical AMD increased from 35.3% in phase I to 46.0% in phase V (Table 2, Fig 1). The raw data for the AMD subtypes are presented in S1 Table.

**Table 2:** Prevalence rates of AMD subtypes.

AMD Subtype	Phase					Total
	I	II	III	IV	V	
Typical AMD	102 (35.3%)	115 (42.3%)	172 (53.1%)	153 (46.6%)	178 (46.0%)	720 (45.0%)
PCV	158 (54.7%)	131 (48.2%)	130 (40.1%)	136 (41.5%)	178 (46.0%)	733 (45.8%)
Combined	16 (5.5%)	14 (5.1%)	7 (2.2%)	2 (0.6%)	10 (2.6%)	49 (3.1%)
RAP	13 (4.5%)	12 (4.4%)	15 (4.6%)	37 (11.3%)	21 (5.4%)	98 (6.1%)
Total	289	272	324	328	387	1,600

AMD, age-related macular degeneration; PCV, polypoidal choroidal vasculopathy; RAP, retinal angiomatous proliferation.

Of 806 eyes with typical AMD (757 eyes of 720 patients with typical AMD and 49 eyes of 49 patients with combined subtypes), the lesions were classified as predominantly classic CNV in 118 (14.6%) eyes, minimally classic CNV in 234 (29.0%) eyes, and occult with no classic CNV in 454 (56.3%) eyes using FA (Table 3). The raw data for the CNV subtypes are presented in S2 Table.

**Table 3:** Subtypes of CNV detected by fluorescein angiography in typical AMD.

CNV Subtype	Phase (Eyes)					Total (806)
	I (124)	II (138)	III (195)	IV (157)	V (192)	
Predominantly classic CNV; eyes (%)	27 (21.8)	16 (11.6)	26 (13.3)	24 (15.3)	25 (13.0)	118 (14.6)
Minimally classic CNV; eyes (%)	46 (37.1)	46 (33.3)	56 (28.7)	44 (28.0)	42 (21.9)	234 (29.0)
Occult with no classic CNV; eyes (%)	51 (41.1)	76 (55.1)	113 (57.9)	89 (56.7)	125 (65.1)	454 (56.3)

AMD, age-related macular degeneration; CNV, choroidal neovascularization; Phases I, II, III, IV and V, March 2003 to February 2005, March 2005 to February 2007, March 2007 to February 2009, March 2009 to February 2011 and March 2011 to February 2013, respectively.

The prevalence of predominantly and minimally classic CNV decreased from 21.8% and 37.1% in phase I to 13.0% and 21.9% in phase V, respectively. However, the prevalence of occult with no classic CNV increased from 41.1% in phase I to 65.1% in phase V.

### Visual acuity

**Fig 2:**
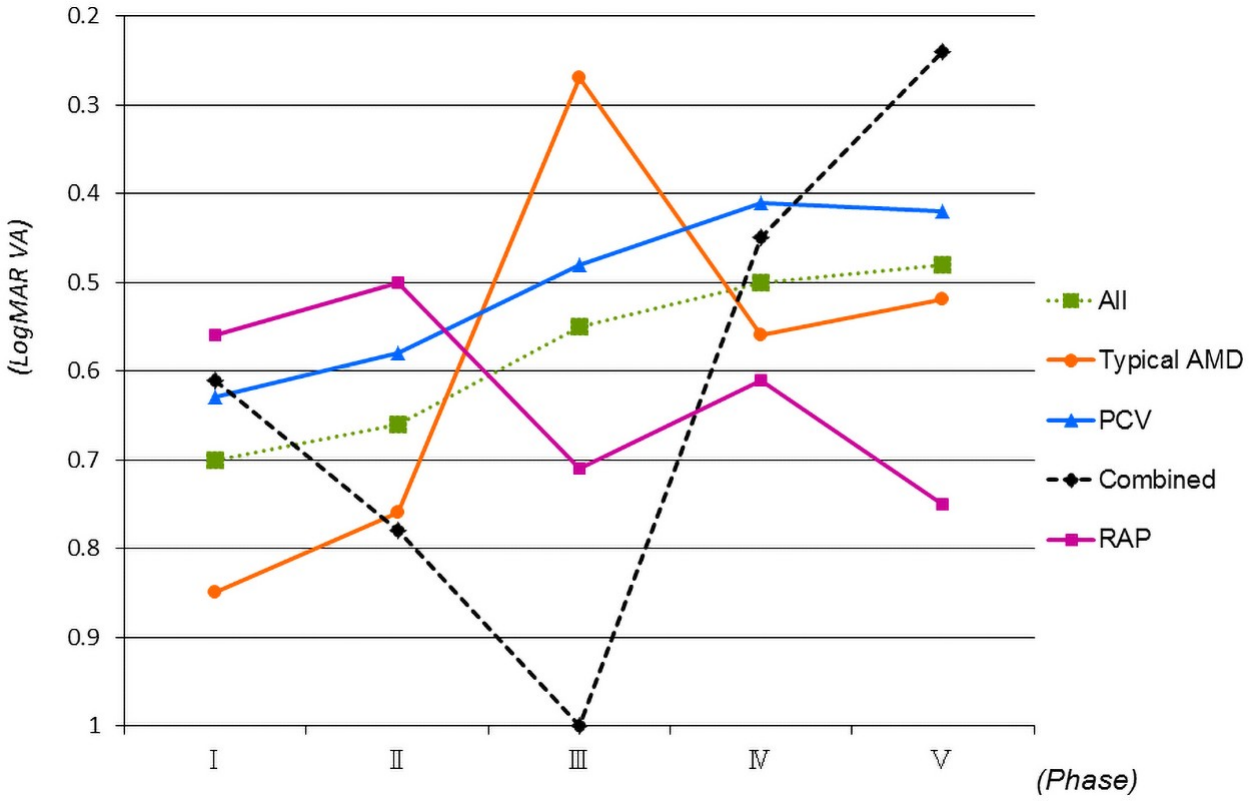
Changes in the mean logarithm of the minimum angle of resolution best-corrected visual acuity (logMAR BCVA) by age-related macular degeneration (AMD) subtypes for the 1,777 eyes. Significantly (*P* = 0.0012, *P*<0.0001, *P*<0.0001, respectively, Mann-Whitney *U*-test) better baseline VAs are seen in phases III, IV, and V compared with phase I. Phases I, II, III, IV, and V spanned, respectively, March 2003 to February 2005, March 2005 to February 2007, March 2007 to February 2009, March 2009 to February 2011 and March 2011 to February 2013. RAP = retinal angiomatous proliferation; combined = combined subtypes; PCV = polypoidal choroidal vasculopathy.

Of the 1,777 study eyes, the mean baseline logMAR BCVA (Snellen equivalent) was 0.57 (20/74). Typical AMD (757 eyes), PCV (798 eyes), RAP (124 eyes) and combined subtypes (98 eyes) had baseline VAs of 0.62 (20/83), 0.50 (20/63), 0.64 (20/86), and 0.62 (20/83), respectively (Table 1). The mean baseline logMAR BCVAs in phases I (344 eyes), II (320 eyes), III (365 eyes), IV (338 eyes), and V (410 eyes) were 0.70 (20/100), 0.66 (20/91), 0.55 (20/71), 0.50 (20/65), and 0.48 (20/60), respectively. A significantly better baseline VAs were seen in phases III, IV, and V compared with phase I (*P* = 0.0012, *P*<0.0001, and *P*<0.0001, respectively, Mann-Whitney *U*-test) (Fig 2).

**Fig 3:**
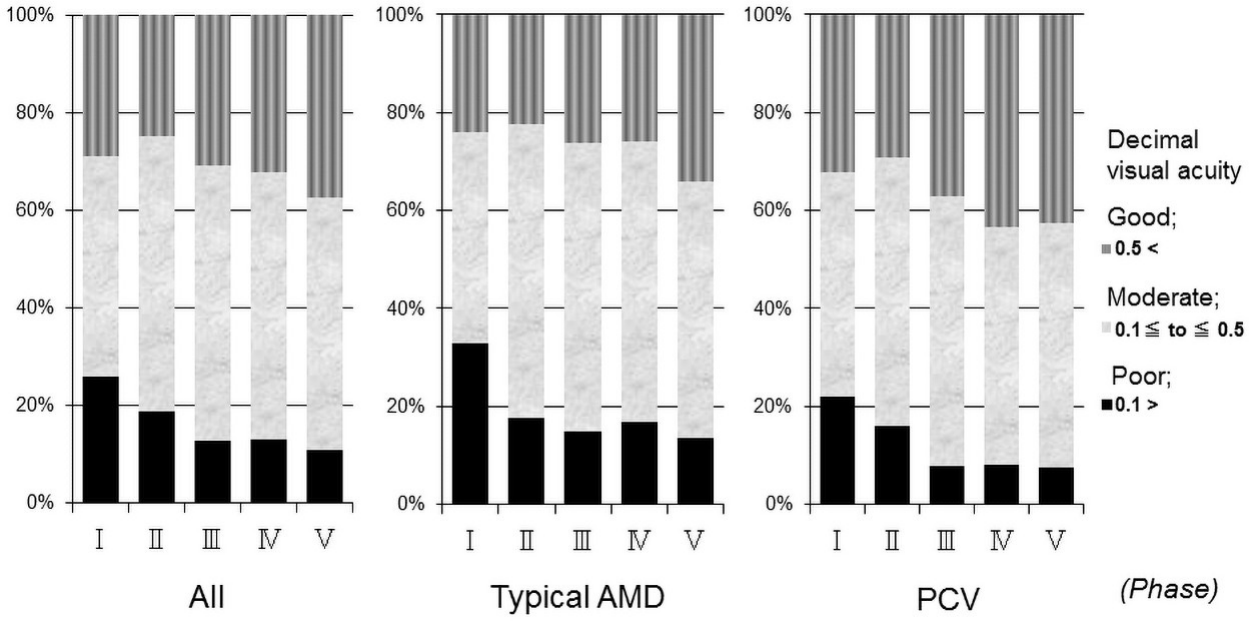
Changes of the distribution in decimal visual acuity (VA) in the 1,777 eyes. The proportion of good baseline VAs increased significantly (*P*<0.05, chi-square test) in phase V (37.4%) compared with phase I (29.1%). The prevalence rates of poor baseline VA in phases I, II, III, IV, and V were 26.0%, 18.8%, 13.0%, 13.0% and 11.0%, respectively, which differed significantly (*P*<0.0001, for all comparisons chi-square test) in phases III, IV, and V compared with phase I. Phases I, II, III, IV, and V spanned, respectively, March 2003 to February 2005, March 2005 to February 2007, March 2007 to February 2009, March 2009 to February 2011 and March 2011 to February 2013. PCV = polypoidal choroidal vasculopathy; AMD = age-related macular degeneration.

The prevalence rates of good baseline VAs in all eyes in phases I, II, III, IV, and V were 29.1%, 24.6%, 29.9%, 32.2% and 37.4% respectively (Fig 3). The percentages of good baseline VAs increased significantly (*P*<0.05, chi-square test) in phase V (37.4%) compared with phase I (29.1%). The prevalence rates of poor baseline VAs in phases I, II, III, IV, and V were 26.0%, 18.8%, 13.0%, 13.0%, and 11.0%, respectively (Fig 3), with significant differences in phases III, IV, and V compared with phase I (*P*<0.0001 for all comparisons by the chi-square test).

Of the 757 eyes with typical AMD, the proportion of good baseline VAs increased significantly in phase V (34.1%) compared with phase I (23.5%) (*P*<0.05, chi-square test). The proportion of poor baseline VAs decreased significantly (*P*<0.001, chi-square test) in phase V (13.7%) compared with phase I (33.1%). Of the 798 eyes with PCV, although there was no significant (*P* = 0.058) difference in the percentages of good baseline VAs between phases I and V, the percentage of poor baseline VA significantly (*P*<0.01, chi-square test) decreased in phase V (7.6%) compared with phase I (21.5%).

### Lesion size

**Fig 4:**
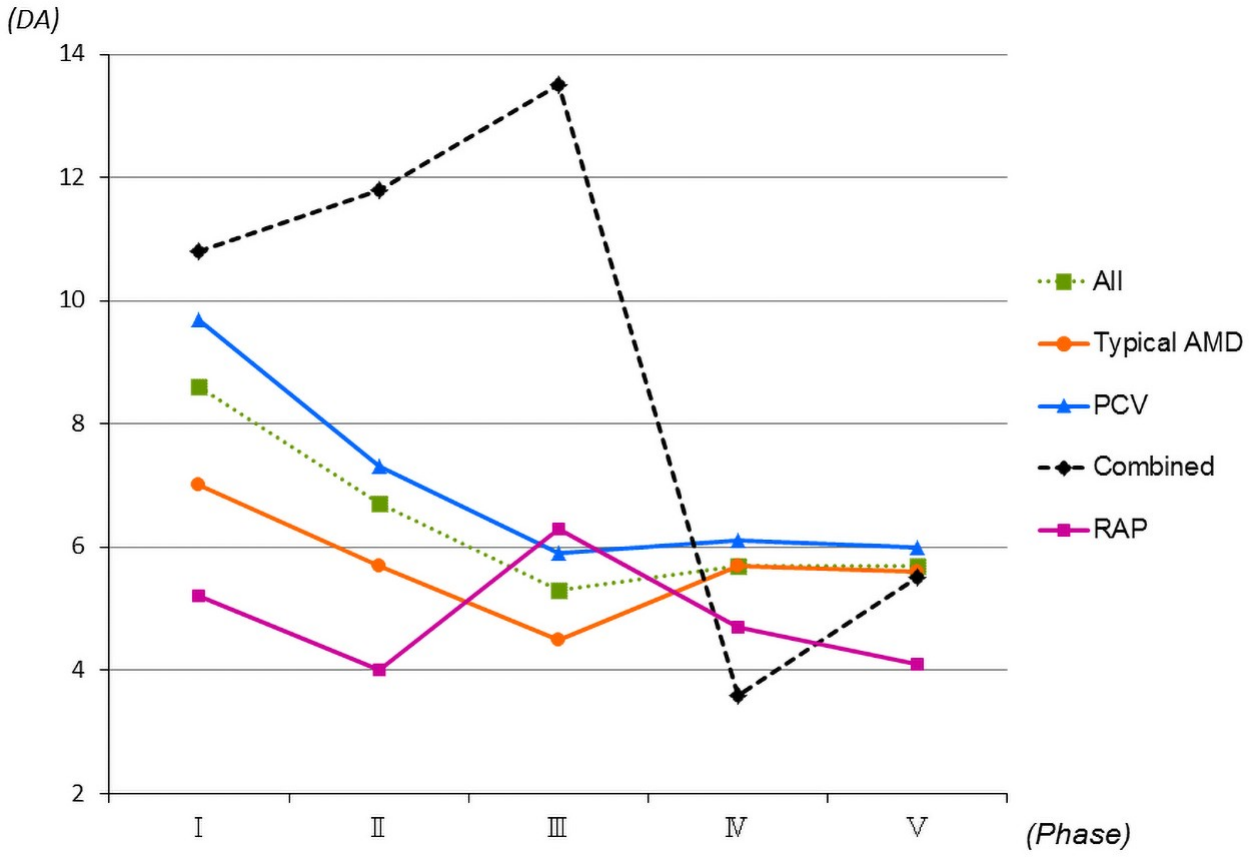
Changes in the mean lesion sizes by age-related macular degeneration (AMD) subtypes in 1,777 eyes. The mean lesion sizes of all eyes are significantly (*P*<0.0001, for all comparisons Mann-Whitney *U*-test) smaller in phases III, IV, and V compared with phase I. Phases I, II, III, IV, and V span, respectively, March 2003 to February 2005, March 2005 to February 2007, March 2007 to February 2009, March 2009 to February 2011 and March 2011 to February 2013. DA = disc area; RAP = retinal angiomatous proliferation; combined = combined subtypes; PCV = polypoidal choroidal vasculopathy.

Of the 1,777 study eyes, the mean lesion size measured by FA was 6.2 DA. The mean lesion sizes in typical AMD (757 eyes), PCV (798 eyes), and RAP (124 eyes) were 5.6, 7.0, and 4.8 DA, respectively (Table 1). No significant differences were seen between typical AMD and PCV (*P* = 0.11, Mann-Whitney *U*-test) and RAP and PCV (*P* = 0.059, Mann-Whitney *U*-test). The mean lesion sizes in phases I (344 eyes), II (320 eyes), III (365 eyes), IV (338 eyes), and V (410 eyes) were 8.6, 6.7, 5.3, 5.7, and 5.7 DA, respectively. The sizes were significantly (*P*<0.0001, for all comparisons, by the Mann-Whitney *U*-test) smaller in phases III, IV, and V compared with phase I (Fig 4).

**Fig 5:**
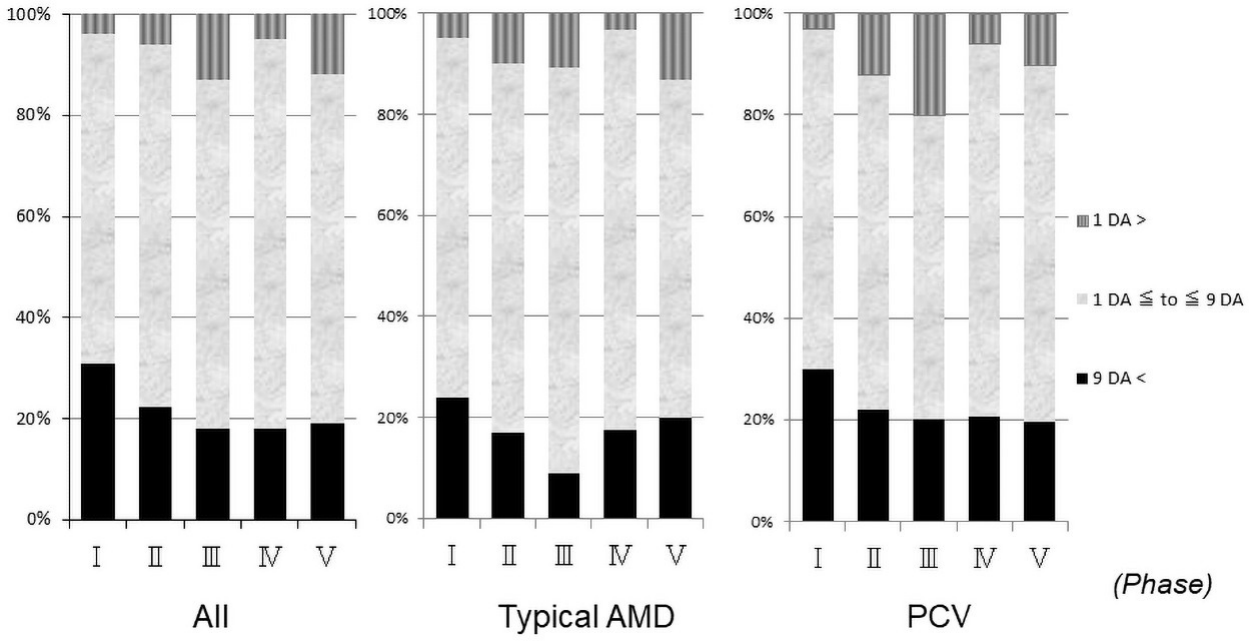
Changes of the distribution in the lesion sizes for 1,777 eyes. The prevalence rates of large lesions in phases II, III, IV, and V are significantly (*P* = 0.022, *P*<0.001, *P*<0.001, *P*<0.001, respectively, chi-square test) smaller compared with phase I. Phases I, II, III, IV, and V spanned, respectively, March 2003 to February 2005, March 2005 to February 2007, March 2007 to February 2009, March 2009 to February 2011 and March 2011 to February 2013. DA = disc area; PCV = polypoidal choroidal vasculopathy; AMD = age-related macular degeneration.

The prevalence rates of large lesions in phases I, II, III, IV, and V were 30.8%, 22.1%, 17.9%, 18.0%, and 19.2%, respectively (Fig 5), with significant (*P* = 0.022, *P*<0.001, *P*<0.001, *P*<0.001, respectively, chi-square test) differences seen in phases II, III, IV, and V compared with phase I.

### Other subjects

Serous or hemorrhagic pigment epithelial detachments (PEDs) was seen in 133 patients (18.5%) with typical AMD, 237 patients (32.3%) with PCV, 21 patients (42.9%) with combined subtypes, and 58 patients (59.2%) with RAP (Table 4). The raw data for the presence of PEDs are presented in S1 Table.

**Table 4:** Clinical characteristics of patients with PED in each AMD subtype.

Characteristic	Typical AMD (n = 720)	PCV (n = 733)	Combined (n = 49)	RAP (n = 98)
Patients with PED (%)	133 (18.5%)	237 (32.3%)	21 (42.9%)	58 (59.2%)
Affected eye (%)				
Bilateral	2 (1.5%)	10 (4.2%)	3 (14.3%)	8 (13.8%)
Unilateral	131(98.5%)	227 (95.8%)	18 (85.7%)	50 (86.2%)

PED, pigment epithelial detachment; AMD, age-related macular degeneration; PCV, polypoidal choroidal vasculopathy; RAP, retinal angiomatous proliferation.

The percentages of PEDs were significantly (*P*<0.0001, for all comparisons by the chi-square test) higher in PCV, combined subtypes, and RAP compared with typical AMD. Although there was no significant (*P* = 0.079) difference for the percentages of PEDs between RAP and combined subtypes, eyes with RAP had a significantly (*P*<0.0001, for both comparisons by the chi-square test) higher tendency of having a PED compared with typical AMD and PCV.

**Fig 6:**
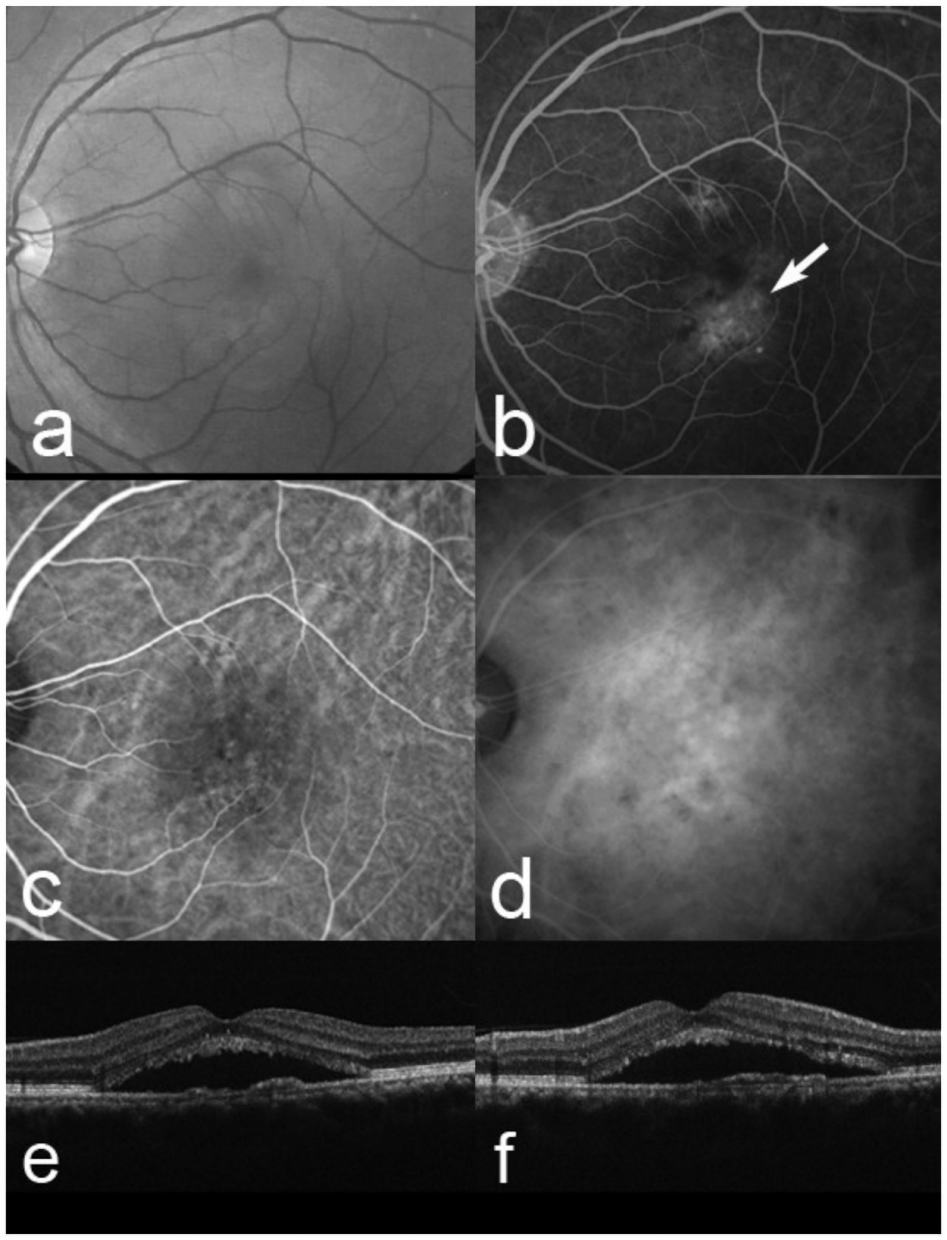
A 56-year-old man whose diagnosis changed from typical age-related macular degeneration (AMD) to polypoidal choroidal vasculopathy. At baseline, the best-corrected visual acuity (VA) is 0.7 decimal VA (Snellen equivalent, 20/28) in the left eye. (a) A red-free fundus photograph shows a serous retinal detachment (SRD). (b) Fluorescein angiography shows occult with no classic choroidal neovascularization (CNV) (arrow). Early-phase (c) and late-phase (d) indocyanine green angiography images show no polypoidal lesions. Horizontal (e) and a vertical (f) optical coherence tomography images show a SRD. This patient was diagnoses with typical AMD associated with occult with no classic CNV.

**Fig 7:**
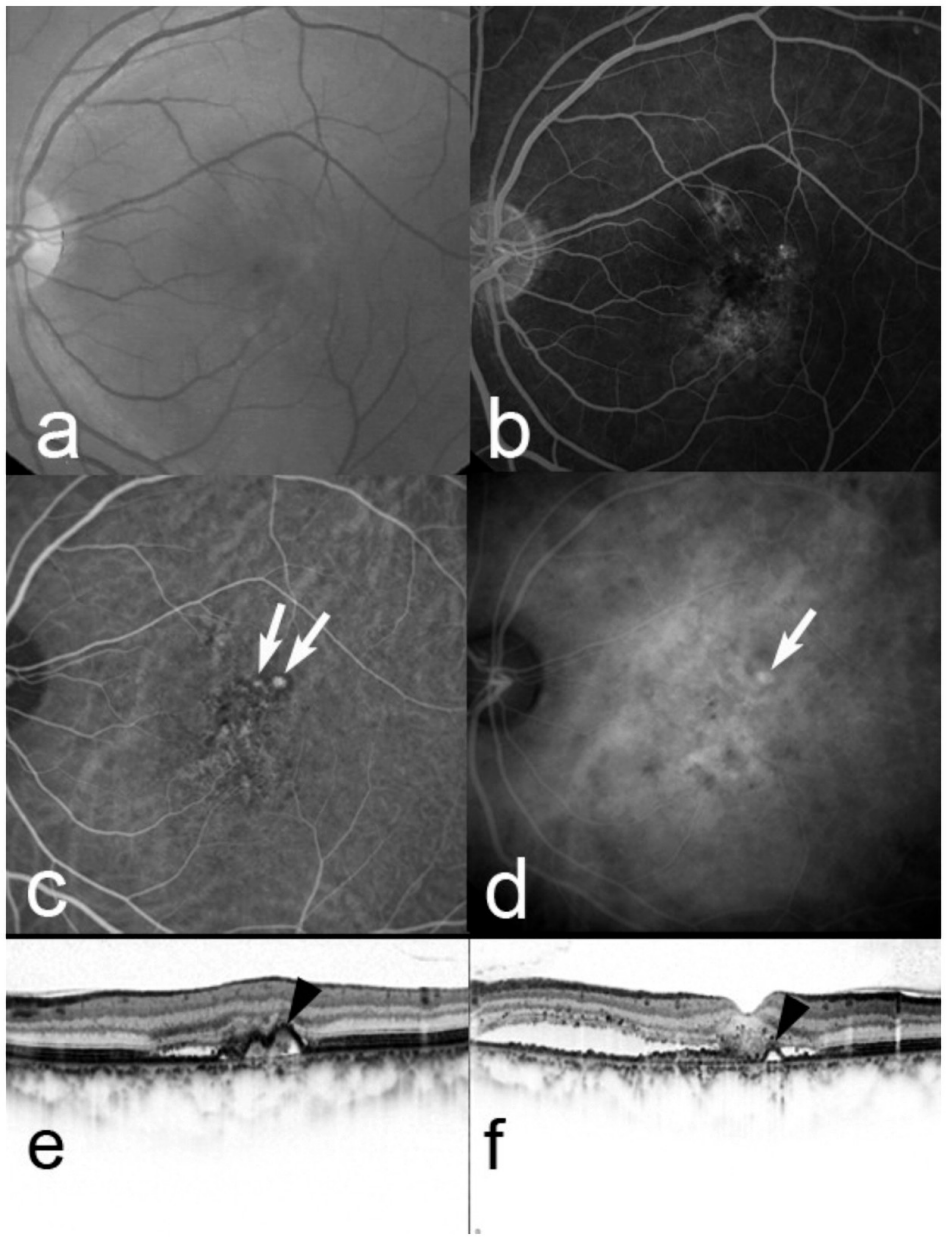
Five months after baseline in the same case as in Fig 6. The decimal visual acuity (VA) remains 0.7. A red-free fundus photograph (a) and fluorescein angiography (b) show a serous retinal detachment (SRD) remaining and occult with no classic choroidal neovascularization. Early-phase (c) and late-phase (d) indocyanine green angiography (ICGA) images clearly show branching choroidal vascular networks and polypoidal lesions (arrows). Horizontal (e) and vertical (f) optical coherence tomography images show a SRD and anterior protrusion of the retinal pigment epithelial line (arrowheads), which corresponds to the polypoidal lesions on ICGA. The diagnosis should be changed to polypoidal choroidal vasculopathy.

Of the 757 eyes with typical AMD, 10 (1.3%) eyes changed to PCV a mean of 22.8 months after baseline (Figs 6 and 7). All 10 eyes had occult CNV at baseline (Fig 6).

**Fig 8:**
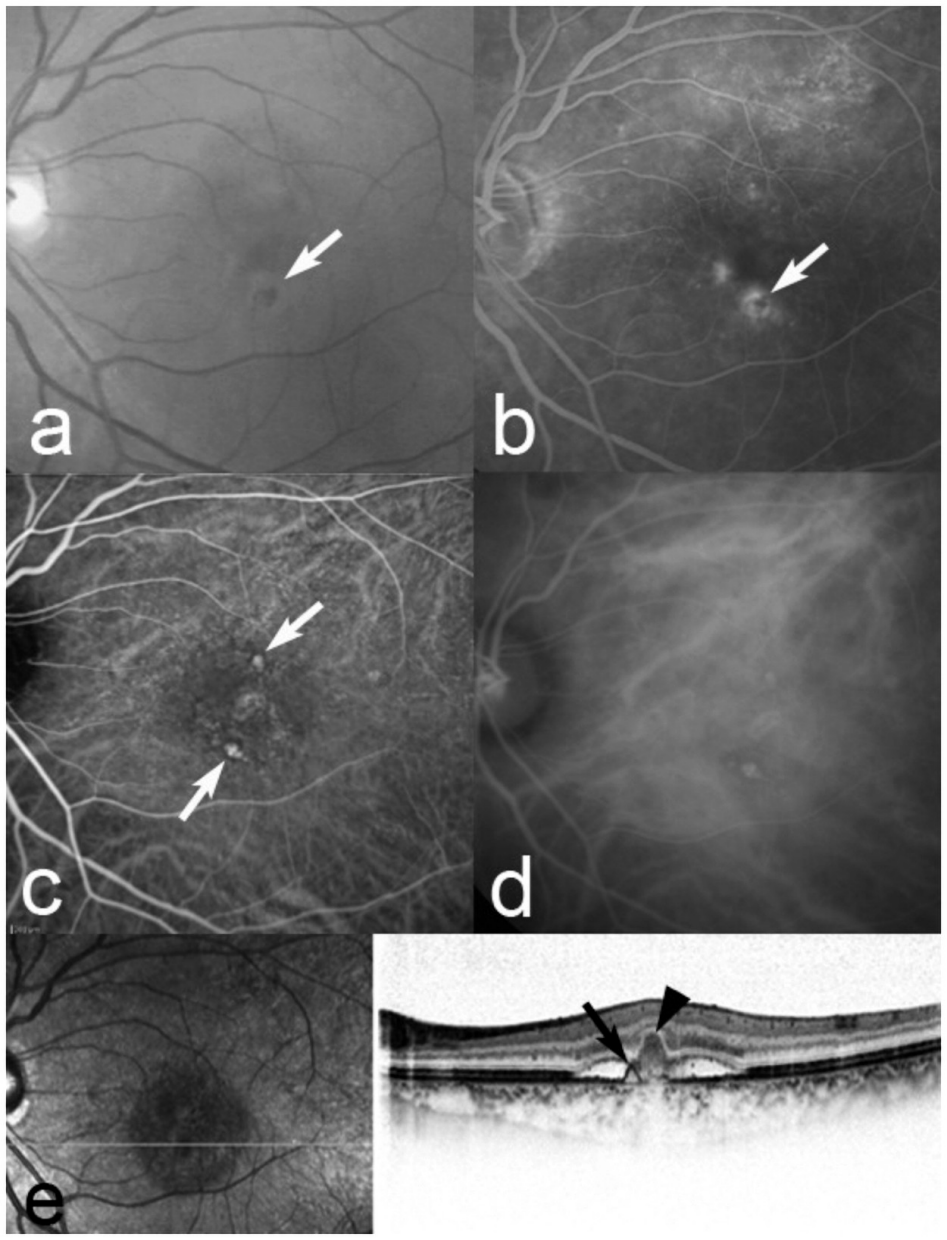
A 69-year-old man with fibrin associated with polypoidal choroidal vasculopathy. At baseline, the best-corrected visual acuity (BCVA) is 1.0 decimal VA (Snellen equivalent, 20/20) in the left eye. (a) A red-free fundus photograph shows an orange-red lesion surrounded by grayish-white fibrinous exudates (arrow). (b) Fluorescein angiography (FA) shows leakage from fibrin (arrow). Early-phase (c) and late-phase (d) indocyanine green angiography (ICGA) images clearly show polypoidal lesions (arrows). A horizontal (e) optical coherence tomography image shows a serous retinal detachment and anterior protrusion of a highly reflective retinal pigment epithelium (RPE) line (arrow), which corresponds to the polypoidal lesions on ICGA. A B-scan image also shows grayish-white fibrinous exudates as highly homogenous reflective masses bridging the neurosensory retina and the RPE (arrowhead), which corresponds to fibrinous exudates on biomicroscopy and FA.

Of the 798 eyes with PCV, 50 (6.3%) eyes had polypoidal lesions with fibrin (Fig 8). Thirty-three (4.1%) eyes had both polypoidal lesions and type 2 CNV (diagnosed by FA and OCT). The mean lesion sizes in eyes with PCV with fibrin and type 2 CNV were 2.3 and 5.6 DAs, which differed significantly (*P*<0.0001, Mann-Whitney *U* test). In the PCV eyes with fibrin, the lesion size was also significantly (*P*<0.0001, Mann-Whitney *U*-test) smaller than that in the PCV eyes without fibrin.

## Discussion

The current study identified the clinical characteristics of AMD in Japanese patients over 10 years, changes in the percentages of the AMD subtypes, BCVA levels, and lesion sizes at baseline.

PCV is highly prevalent in Japanese AMD, since we reported that PCV was found in 54.7% of patients with neovascular AMD.^7^ In other Asian countries, PCV was diagnosed in 37 (22.3%) of 166 Chinese patients with AMD and 79 (24.6%) of 321 patients with AMD in Korea [18,19]. In the current study, PCV was diagnosed in 733 (45.8%) of the 1,600 patients. In contrast, Yannuzzi and associates reported that PCV was diagnosed in 13 (7.8%) of 167 Caucasian patients [20]; in other Caucasian populations, PCV was diagnosed in 14 (4%) of 374 eyes with occult CNV in Germany [21], 19 (9.8%) of 194 patients with AMD in Italy [22], and 22 (8.2%) of 268 patients with AMD in Greece [23].

The current study evaluated the changes in the prevalence rates of AMD subtypes over 10 years by dividing that time period into five phases of 2 years each to compare the previous report in phase I [7]. The prevalence rates of PCV and typical AMD, respectively, decreased from 54.7% in phase I to 46.0% in phase V and increased from 35.3% in phase I to 46.0% in phase V. To our knowledge, the current study is the first to demonstrate changes in the prevalence rates of AMD subtypes. High-resolution HRA2 can help accurately diagnose PCV [9]. We used HRA2 from August 2005, which might explain the increased prevalence of PCV. However, the current results showed that the proportion of PCV decreased after the HRA2 was introduced. Although the reason for that decrease is unclear, it might not depend on development of the high-resolution ICGA instrument.

Anti-VEGF treatment using ranibizumab or aflibercept has become standard therapy for AMD [4–6]. Moreover, a new anti-VEGF drug, brolucizumab was reported to be non-inferior to aflibercept in major clinical trials [10,11]. In contrast, the 7-year results with ranibizumab showed a mean 8.6-letter decrease in the BCVA [24]. Several studies have reported baseline predictors of visual outcomes with anti-VEGF drugs, i.e., the baseline VA was associated with better VA at 1 year and less gain in VA at 1 and 2 years [25,26]. We also reported the efficacy of treating patients with AMD with good baseline VA exceeding 20/40 [27]. Significantly better baseline VAs in phases III, IV, and V compared with phase I (Fig 2) might be related to increased interests for AMD because of reports for newly or better treatment modality. However, no studies have reported changes in the percentages of the baseline VAs. The current study found that the percentages of good baseline VAs increased significantly in phase V (37.4%) compared with phase I (29.1%). The prevalence of poor baseline VAs in phases III, IV, and V were significantly smaller compared with phase I. There also was the same tendency in typical AMD and PCV except for the proportion of good baseline VA associated with PCV between phases I and V. These results were correlated with the history of treatments for AMD, increasing concerns about AMD annually, and earlier medical examinations in Japan, which might be important for thinking about better treatments for patients with AMD in the future.

Lesion size has been an important factor in making treatment decisions for patients with AMD in clinical trials and individual clinics. Most clinical trials had inclusion criteria that limited lesion sizes, e.g., less than 5,400 microns as in the ANCHOR study and less than 12 disc areas in the MARINA and VIEW studies [4–6]. However, no studies have evaluated the percentages of the lesion sizes at baseline or changes in those percentages. In the current study, the mean lesion sizes were 8.6, 6.7, 5.3, 5.7, and 5.7 DA, respectively, in phases I, II, III, IV, and V, which showed that the sizes were significantly smaller in phases III, IV, and V compared with phase I. Moreover, the prevalence rates of large lesions were 30.8%, 22.1%, 17.9%, 18.0%, and 19.2% in phases I, II, III, IV, and V, respectively, which decreased significantly in phases II, III, IV, and V compared with phase I. These results might be related to higher concern about AMD annually and reflect new information about the disease.

The presence of a serous or hemorrhagic PED was considered a risk factor for development of RPE tears and lower VA gains after anti-VEGF therapy [26,28–30]. Table 4 shows the prevalence rates of serous and hemorrhagic PEDs by AMD subtype. The current study showed that RAP is associated significantly with a higher tendency for development of a PED compared with typical AMD and PCV. Moreover, PEDs were highly associated with PCV compared with typical AMD. These results might help accurately diagnose and devise strategies for better treatments for patients with AMD.

Some patients have uncertain diagnoses regardless of whether they have PCV or typical AMD. We reported a case that was ultimately diagnosed with PCV 2.5 years after baseline because the lesion masqueraded as typical AMD [14]. Davis et al. also reported that 16 (59%) of 27 eyes had PCV that masqueraded as typical AMD at baseline and the mean time to PCV diagnosis was 17.5 months [31]. In the current study, 10 (1.3%) of the 757 eyes with typical AMD were changed to a diagnosis of PCV a mean of 22.8 months after baseline. A difference was seen in the proportion of PCV masquerading as typical AMD between the study of Davis et al. [31] and the current study due to differences in inclusion criteria, case, and routine examination with ICGA. Caution should be taken when considering any additional anti-VEGF injection for typical AMD with a poor response to past injections, because these cases might have PCV.

PCV sometimes has subretinal fibrin, which might be misdiagnosed as classic CNV [32]. We should distinguish between subretinal fibrin and type 2 CNV because the visual prognoses in eyes with type 2 CNV might differ according to past reports. In the current study, 50 (6.3%) of the 798 eyes with PCV were diagnosed with subretinal fibrinous exudates based on additional OCT findings.

Type 2 CNV can be seen in eyes with PCV. In the current study, 33 (4.1%) of the 798 eyes with PCV had both polypoidal lesions and type 2 CNV. The reason for the presence of polypoidal lesions and type 2 CNV, defined as PCV plus type 2 CNV, in the same eye is unclear. Vascular abnormalities in eyes with PCV seem to be variants of CNV and are referred to as polypoidal CNV. Moreover, PCV and typical AMD are similar in some pathophysiologic aspects according to genetic reports [8]. These results can help with an understanding of the presence of PCV plus type 2 CNV. In addition, we reported that eyes with PCV associated with type 2 CNV needed significantly (*P*<0.001) more treatments than eyes without it over 3 years [33]. Therefore, accurate diagnosis of PCV whether type 2 CNV is present or not is needed to assure that appropriate treatment is administered.

Although the prevalence of RAP has been reported to be low i.e., 10–15% in Caucasian patients and 4.5% in Japanese patients [7,34], it was found to be higher in the phase IV (11.3%). It might be related to beginning and increased interests for RAP after reports for good visual outcomes using combined therapy anti-VEGF and PDT [35,36]. Moreover, newly methods using fundus autofluorescence imaging may be helpful for detected before the onset of RAP lesions, which may affect the prevalence of RAP [37].

Pachychoroid neovasculopathy (PNV) has been reported as a clinical entity related to CNV, characterized by thick choroid without drusen, in 2014 [38]. Although, PNV often masquerades as typical AMD, OCT angiography can detect easily PNV with non-invasive [39]. We should continue to study latest data after phase V using OCT angiography.

A newly anti-VEGF drug brolucizumab is the smallest molecule weight of a 28 kDa humanized single-chain antibody fragment and has been approved to use unprecedented 12-week dosing schedule, which can be able to achieve dry macular and prevent frequent visits for AMD patients [10,11]. However, IOI after injection of brolucizumab has been reported to be incidence of 4.7 to 19% [10,40,41]. Therefore, it should be needed that the different proper use of anti-VEGF drugs using ranibizumab, aflibercept and brolucizumab according to CNV subtypes. It also important to recognize securely the clinical characteristics of AMD, changes in the percentages of the AMD subtypes, BCVA levels, and lesion sizes for long-term.

The present study was associated with some limitations. The current study was a single-center, retrospective, nonrandomized trial and lack of latest data after phase V. Further long-term prospective, randomized studies with larger cohorts are needed to determine the clinical characteristics of AMD.

In conclusion, although the prevalence of PCV decreased from 54.7% in phase I to 46.0% at phase V, the current study showed highly prevalent of PCV in Japanese patients with AMD compared with Caucasian patients as in our previous report. Moreover, the better baseline VAs and smaller lesion sizes might be related to development of improved treatments.

## Supporting information

S1 Table Raw data for the patient characteristics. (DOCX)

S2 Table Raw data for the CNV subtypes detected by fluorescein angiography in typical AMD. (DOCX)
