# Supplementary material for: Long-term characteristics of exudative age-related macular degeneration in Japanese patients
Source: PLoS One. 2021 Dec 14;16(12):e0261320. doi: 10.1371/journal.pone.0261320 (PMC8670714; doi:10.1371/journal.pone.0261320)
Supplement: S1 Table — (DOCX) [file pone.0261320.s001.docx]

Table S1: Raw data of Patient Characteristics

| **Phase I** |  |  |  |  |  |  |
| --- | --- | --- | --- | --- | --- | --- |
| patient No. | age | sex | AMD Subtype | LogMAR BCVA | PED | Lesion size (DA) |
| 1 | 56 | Ｆ | tAMD | 0.70 | no | 3 |
| 2 | 75 | Ｆ | tAMD | 1.22 | no | 5 |
| 3 | 81 | M | tAMD | 1.00 | no | 11 |
| 4 | 85 | M | tAMD | 1.52 | no | 14 |
| 5 | 70 | M | tAMD | 1.15 | no | 9 |
| 6 | 62 | M | tAMD | 0.52 | yes | 10 |
| 7 | 55 | Ｆ | tAMD | 0.40 | no | 3 |
| 8 | 75 | Ｆ | tAMD | 1.00 | no | 8 |
| 9 | 72 | Ｆ | tAMD | 0.70 | no | 3 |
| 10 | 55 | Ｆ | tAMD | 1.00 | no | 4 |
| 11 | 63 | Ｆ | tAMD | 0.40 | no | 3 |
| 12 | 53 | M | tAMD | -0.18 | yes | 8 |
| 13 | 65 | M | tAMD | 0.70 | no | 3 |
| 14 | 82 | Ｆ | tAMD | 2.00 | yes | 23 |
| 15 | 71 | M | tAMD | -0.08 | no | 2 |
| 16 | 68 | Ｆ | tAMD | 1.70 | no | 4 |
| 17 | 76 | M | tAMD | -0.08 | no | 14 |
|  |  |  | tAMD | 1.10 | no | 11 |
| 18 | 73 | M | tAMD | 0.52 | no | 4 |
| 19 | 75 | M | tAMD | -0.08 | no | 7 |
|  |  |  | tAMD | 1.15 | no | 3 |
| 20 | 81 | M | tAMD | 0.22 | yes | 24 |
| 21 | 83 | M | tAMD | -0.08 | no | 2 |
|  |  |  | tAMD | 1.05 | no | 17 |
| 22 | 77 | M | tAMD | 1.15 | yes | 7 |
| 23 | 77 | M | tAMD | 1.22 | no | 25 |
| 24 | 50 | M | tAMD | 0.70 | no | 1 |
| 25 | 61 | M | tAMD | 0.00 | no | 2 |
| 26 | 66 | M | tAMD | 1.00 | no | 6 |
| 27 | 72 | M | tAMD | 0.10 | no | 3 |
| 28 | 76 | M | tAMD | 1.70 | no | 12 |
| 29 | 68 | Ｆ | tAMD | 1.10 | no | 19 |
| 30 | 78 | F | tAMD | 0.70 | no | 2 |
| 31 | 79 | Ｆ | tAMD | 0.40 | no | 1 |
| 32 | 55 | F | tAMD | 0.70 | yes | 3 |
| 33 | 85 | Ｆ | tAMD | 1.00 | yes | 8 |
| 34 | 80 | M | tAMD | 0.30 | no | 5 |
| 35 | 78 | Ｆ | tAMD | 2.00 | no | 9 |
| 36 | 91 | M | tAMD | 1.10 | no | 3 |
| 37 | 64 | Ｆ | tAMD | 1.00 | no | 11 |
| 38 | 82 | M | tAMD | 0.40 | yes | 14 |
| 39 | 51 | M | tAMD | 1.10 | no | 3 |
| 40 | 78 | M | tAMD | 0.70 | yes | 3 |
| 41 | 75 | Ｆ | tAMD | 0.00 | yes | 1 |
| 42 | 67 | M | tAMD | 0.05 | no | 1 |
| 43 | 85 | M | tAMD | 1.30 | no | 7 |
| 44 | 84 | M | tAMD | 1.40 | no | 19 |
| 45 | 56 | M | tAMD | 0.00 | no | 1 |
| 46 | 86 | M | tAMD | 0.70 | yes | 5 |
| 47 | 74 | M | tAMD | 1.52 | no | 12 |
| 48 | 85 | Ｆ | tAMD | 1.00 | no | 2 |
| 49 | 74 | M | tAMD | 1.00 | no | 7 |
| 50 | 73 | M | tAMD | 0.22 | yes | 3 |
| 51 | 78 | Ｆ | tAMD | 1.00 | no | 3 |
| 52 | 58 | M | tAMD | 3.00 | no | 12 |
| 53 | 76 | Ｆ | tAMD | 1.40 | no | 3 |
| 54 | 60 | F | tAMD | 0.70 | no | 12 |
|  |  |  | tAMD | 1.00 | no | 5 |
| 55 | 84 | M | tAMD | 1.00 | no | 18 |
| 56 | 70 | M | tAMD | 1.00 | no | 8 |
|  | 71 | M | tAMD | 0.70 | no | 8 |
| 57 | 88 | Ｆ | tAMD | 0.05 | no | 1 |
| 58 | 65 | Ｆ | tAMD | 0.15 | no | 1 |
| 59 | 69 | M | tAMD | 0.00 | no | 2 |
| 60 | 83 | F | tAMD | 1.70 | yes | 18 |
| 61 | 77 | M | tAMD | 0.05 | yes | 4 |
| 62 | 81 | M | tAMD | 1.05 | no | 9 |
| 63 | 84 | M | tAMD | 1.22 | no | 5 |
| 64 | 79 | M | tAMD | 0.52 | no | 2 |
| 65 | 78 | M | tAMD | 1.10 | no | 14 |
| 66 | 53 | M | tAMD | 0.52 | no | 4 |
| 67 | 75 | F | tAMD | 0.70 | no | 6 |
| 68 | 89 | M | tAMD | 0.70 | no | 8 |
| 69 | 55 | M | tAMD | 1.00 | no | 4 |
| 70 | 79 | M | tAMD | 1.22 | no | 9 |
| 71 | 76 | M | tAMD | 0.30 | no | 4 |
| 72 | 83 | M | tAMD | 1.00 | no | 6 |
| 73 | 79 | M | tAMD | 1.40 | no | 10 |
| 74 | 70 | M | tAMD | 0.05 | no | 3 |
| 75 | 76 | M | tAMD | 1.00 | yes | 32 |
| 76 | 82 | M | tAMD | 0.52 | no | 6 |
| 77 | 62 | M | tAMD | 0.40 | yes | 33 |
| 78 | 81 | M | tAMD | 1.00 | no | 3 |
| 79 | 61 | M | tAMD | 1.00 | yes | 3 |
| 80 | 73 | M | tAMD | 1.00 | no | 3 |
| 81 | 68 | F | tAMD | 1.10 | no | 6 |
| 82 | 77 | M | tAMD | 0.15 | no | 3 |
| 83 | 80 | M | tAMD | 1.05 | no | 10 |
| 84 | 66 | M | tAMD | 1.15 | no | 5 |
| 85 | 76 | M | tAMD | 0.52 | yes | 3 |
| 86 | 74 | M | tAMD | 0.15 | no | 2 |
| 87 | 74 | Ｆ | tAMD | 0.00 | yes | 4 |
| 88 | 82 | M | tAMD | 2.00 | no | 9 |
| 89 | 79 | M | tAMD | 1.00 | no | 3 |
|  |  |  | tAMD | 0.15 | no | 3 |
| 90 | 73 | M | tAMD | 0.05 | no | 3 |
| 91 | 77 | M | tAMD | 0.30 | yes | 8 |
| 92 | 78 | M | tAMD | 4.00 | yes | 15 |
| 93 | 73 | M | tAMD | 1.10 | no | 6 |
| 94 | 85 | Ｆ | tAMD | 1.15 | no | 3 |
| 95 | 83 | M | tAMD | 1.10 | no | 6 |
| 96 | 66 | M | tAMD | 1.00 | no | 2 |
| 97 | 77 | M | tAMD | 1.10 | no | 9 |
| 98 | 78 | M | tAMD | 1.40 | no | 10 |
| 99 | 73 | M | tAMD | 1.00 | no | 13 |
| 100 | 64 | Ｆ | tAMD | 1.15 | no | 4 |
| 101 | 76 | M | tAMD | 0.40 | no | 4 |
| 102 | 77 | M | tAMD | 0.22 | yes | 4 |
| 103 | 68 | M | PCV | 1.00 | no | 12 |
|  |  |  | PCV | -0.18 | yes | 4 |
| 104 | 76 | M | PCV | 1.15 | no | 38 |
| 105 | 81 | M | PCV | 0.40 | no | 25 |
| 106 | 89 | M | PCV | 0.52 | no | 3 |
| 107 | 72 | M | PCV | 0.22 | yes | 5 |
| 108 | 68 | M | PCV | 0.52 | yes | 11 |
| 109 | 67 | Ｆ | PCV | -0.08 | no | 4 |
| 110 | 61 | M | PCV | 0.82 | no | 3 |
| 111 | 70 | M | PCV | 0.40 | yes | 6 |
| 112 | 85 | M | PCV | 1.00 | no | 3 |
|  |  |  | PCV | 1.52 | yes | 12 |
| 113 | 68 | Ｆ | PCV | 1.70 | no | 9 |
| 114 | 71 | M | PCV | 0.70 | yes | 13 |
| 115 | 77 | M | PCV | 1.52 | yes | 47 |
|  |  | M | PCV | 0.30 | yes | 14 |
| 116 | 79 | M | PCV | 1.10 | yes | 9 |
|  |  | M | PCV | 1.00 | yes | 16 |
| 117 | 78 | M | PCV | 1.00 | yes | 41 |
| 118 | 76 | M | PCV | 1.00 | no | 3 |
| 119 | 80 | Ｆ | PCV | 1.70 | yes | 22 |
| 120 | 74 | M | PCV | 0.10 | no | 2 |
| 121 | 79 | M | PCV | 0.40 | no | 41 |
| 122 | 73 | M | PCV | 0.15 | yes | 5 |
|  |  |  | PCV | 0.10 | yes | 10 |
| 123 | 87 | M | PCV | 2.00 | no | 25 |
|  |  |  | PCV | 1.52 | no | 25 |
| 124 | 73 | M | PCV | 1.00 | no | 24 |
| 125 | 77 | M | PCV | 0.70 | no | 10 |
|  |  | M | PCV | 0.22 | no | 10 |
| 126 | 72 | M | PCV | 0.22 | no | 2 |
| 127 | 74 | M | PCV | 1.00 | yes | 12 |
|  |  |  | PCV | 1.00 | yes | 14 |
| 128 | 71 | M | PCV | 0.22 | no | 2 |
| 129 | 72 | M | PCV | 1.05 | no | 27 |
| 130 | 60 | M | PCV | 0.70 | yes | 20 |
| 131 | 71 | Ｆ | PCV | 0.22 | no | 3 |
| 132 | 87 | Ｆ | PCV | 1.15 | no | 6 |
| 133 | 84 | M | PCV | 3.00 | no | 44 |
| 134 | 73 | M | PCV | 1.70 | yes | 14 |
| 135 | 77 | Ｆ | PCV | 0.22 | no | 4 |
| 136 | 53 | Ｆ | PCV | 0.00 | no | 3 |
| 137 | 89 | M | PCV | 0.30 | no | 6 |
| 138 | 69 | M | PCV | 0.30 | no | 2 |
|  |  |  | PCV | 0.70 | no | 5 |
| 139 | 66 | M | PCV | 0.82 | no | 2 |
| 140 | 91 | M | PCV | 0.40 | no | 5 |
| 141 | 61 | M | PCV | 0.70 | yes | 6 |
| 142 | 69 | M | PCV | 0.05 | yes | 7 |
| 143 | 78 | M | PCV | 0.70 | yes | 9 |
| 144 | 72 | M | PCV | 0.52 | yes | 19 |
| 145 | 76 | M | PCV | 0.15 | no | 4 |
| 146 | 76 | M | PCV | 0.52 | yes | 14 |
| 147 | 65 | M | PCV | 0.00 | no | 4 |
|  |  |  | PCV | 0.22 | no | 18 |
| 148 | 77 | F | PCV | 0.10 | no | 1 |
| 149 | 88 | M | PCV | 1.00 | yes | 9 |
| 150 | 62 | M | PCV | -0.08 | yes | 7 |
| 151 | 74 | M | PCV | -0.08 | yes | 11 |
| 152 | 64 | M | PCV | 1.15 | no | 11 |
| 153 | 53 | F | PCV | 0.10 | yes | 24 |
| 154 | 78 | Ｆ | PCV | 1.52 | no | 2 |
| 155 | 71 | M | PCV | 0.40 | yes | 3 |
| 156 | 61 | M | PCV | 0.40 | no | 3 |
| 157 | 73 | M | PCV | 0.15 | no | 1 |
| 158 | 60 | Ｆ | PCV | 1.05 | no | 1 |
| 159 | 66 | M | PCV | 0.40 | no | 7 |
|  |  |  | PCV | 0.30 | no | 6 |
| 160 | 66 | M | PCV | 0.30 | yes | 6 |
| 161 | 74 | M | PCV | 1.00 | no | 8 |
|  |  |  | PCV | 0.70 | no | 6 |
| 162 | 79 | F | PCV | 0.30 | no | 2 |
| 163 | 74 | M | PCV | 1.40 | yes | 25 |
| 164 | 76 | M | PCV | 1.10 | no | 7 |
| 165 | 76 | M | PCV | 1.05 | no | 3 |
| 166 | 81 | M | PCV | 1.40 | no | 13 |
|  |  |  | PCV | 0.00 | no | 2 |
| 167 | 75 | M | PCV | 0.30 | no | 1 |
| 168 | 79 | M | PCV | 0.40 | yes | 2 |
|  |  |  | PCV | 0.52 | yes | 6 |
| 169 | 78 | F | PCV | 1.05 | no | 7 |
| 170 | 78 | M | PCV | 1.70 | yes | 19 |
| 171 | 68 | M | PCV | 1.05 | yes | 17 |
| 172 | 76 | M | PCV | 0.22 | no | 3 |
| 173 | 78 | Ｆ | PCV | 0.40 | no | 3 |
| 174 | 80 | Ｆ | PCV | 0.70 | no | 9 |
|  |  |  | PCV | 1.00 | no | 10 |
| 175 | 67 | M | PCV | 0.10 | no | 1 |
| 176 | 88 | M | PCV | 0.52 | no | 3 |
| 177 | 63 | M | PCV | 0.30 | yes | 8 |
| 178 | 77 | Ｆ | PCV | -0.08 | no | 2 |
| 179 | 59 | M | PCV | 2.00 | no | 14 |
| 180 | 83 | M | PCV | 3.00 | yes | 15 |
| 181 | 76 | M | PCV | 1.00 | no | 12 |
|  |  |  | PCV | 0.70 | no | 17 |
| 182 | 66 | M | PCV | 0.00 | no | 2 |
|  |  |  | PCV | 1.10 | no | 6 |
| 183 | 67 | M | PCV | 1.00 | no | 1 |
| 184 | 68 | M | PCV | -0.08 | yes | 34 |
| 185 | 73 | Ｆ | PCV | 0.52 | yes | 13 |
| 186 | 81 | M | PCV | 1.10 | yes | 23 |
| 187 | 79 | Ｆ | PCV | 1.52 | yes | 9 |
| 188 | 77 | M | PCV | 1.00 | yes | 21 |
| 189 | 79 | M | PCV | 0.30 | no | 5 |
| 190 | 75 | M | PCV | 0.70 | no | 9 |
| 191 | 62 | F | PCV | 0.00 | no | 2 |
|  |  |  | PCV | -0.08 | no | 1 |
| 192 | 81 | M | PCV | 0.00 | no | 1 |
|  |  |  | PCV | 0.40 | no | 3 |
| 193 | 75 | M | PCV | 0.52 | no | 5 |
| 194 | 64 | M | PCV | 0.52 | no | 21 |
| 195 | 52 | M | PCV | -0.18 | yes | 13 |
| 196 | 83 | Ｆ | PCV | 0.22 | no | 5 |
|  |  |  | PCV | 0.40 | no | 7 |
| 197 | 75 | M | PCV | 1.00 | no | 3 |
| 198 | 82 | Ｆ | PCV | 0.15 | no | 3 |
| 199 | 86 | M | PCV | 1.40 | no | 13 |
| 200 | 77 | Ｆ | PCV | 0.70 | no | 3 |
| 201 | 75 | M | PCV | 1.00 | no | 3 |
| 202 | 71 | M | PCV | 1.05 | no | 7 |
|  |  |  | PCV | 0.52 | yes | 12 |
| 203 | 78 | M | PCV | 0.00 | yes | 12 |
| 204 | 73 | M | PCV | 0.70 | yes | 15 |
|  |  |  | PCV | 1.00 | no | 5 |
| 205 | 73 | F | PCV | 0.52 | yes | 4 |
| 206 | 80 | M | PCV | 0.22 | no | 7 |
| 207 | 80 | M | PCV | 0.15 | yes | 4 |
|  |  |  | PCV | -0.08 | no | 2 |
| 208 | 78 | M | PCV | 1.00 | no | 3 |
| 209 | 71 | M | PCV | -0.08 | yes | 2 |
| 210 | 67 | M | PCV | 0.10 | no | 2 |
| 211 | 69 | M | PCV | 1.40 | yes | 6 |
| 212 | 68 | Ｆ | PCV | 1.52 | no | 4 |
|  |  |  | PCV | -0.18 | no | 0 |
| 213 | 71 | M | PCV | -0.08 | yes | 2 |
| 214 | 60 | M | PCV | 1.00 | no | 5 |
| 215 | 71 | M | PCV | 1.05 | yes | 41 |
| 216 | 65 | M | PCV | 0.70 | yes | 50 |
| 217 | 80 | M | PCV | 0.10 | no | 29 |
| 218 | 66 | M | PCV | 0.10 | yes | 2 |
| 219 | 69 | M | PCV | 0.10 | no | 2 |
| 220 | 61 | M | PCV | 1.30 | no | 1 |
| 221 | 82 | M | PCV | 0.40 | no | 3 |
| 222 | 67 | Ｆ | PCV | 1.00 | no | 21 |
|  |  |  | PCV | 1.00 | no | 15 |
| 223 | 64 | M | PCV | 0.05 | yes | 13 |
| 224 | 81 | M | PCV | 0.40 | no | 13 |
| 225 | 75 | M | PCV | 0.70 | yes | 7 |
|  |  |  | PCV | 0.15 | yes | 12 |
| 226 | 72 | M | PCV | 0.52 | yes | 9 |
| 227 | 50 | Ｆ | PCV | 0.00 | no | 1 |
| 228 | 62 | Ｆ | PCV | -0.18 | yes | 13 |
|  |  |  | PCV | 1.22 | yes | 35 |
| 229 | 67 | M | PCV | 0.82 | no | 3 |
| 230 | 83 | M | PCV | 0.40 | yes | 7 |
| 231 | 76 | M | PCV | 1.15 | no | 8 |
| 232 | 57 | M | PCV | 0.00 | no | 4 |
|  |  |  | PCV | 1.00 | no | 13 |
| 233 | 72 | Ｆ | PCV | 0.30 | no | 3 |
| 234 | 69 | M | PCV | 0.52 | no | 3 |
| 235 | 78 | Ｆ | PCV | 1.30 | yes | 27 |
| 236 | 77 | M | PCV | 0.15 | no | 7 |
| 237 | 85 | M | PCV | 0.70 | yes | 49 |
| 238 | 68 | M | PCV | 0.70 | no | 2 |
| 239 | 78 | M | PCV | 1.00 | no | 6 |
|  |  |  | PCV | 1.40 | no | 9 |
| 240 | 82 | Ｆ | PCV | 0.05 | no | 3 |
| 241 | 81 | F | PCV | 0.70 | yes | 5 |
| 242 | 72 | M | PCV | 0.30 | no | 2 |
| 243 | 74 | M | PCV | 0.40 | no | 7 |
| 244 | 67 | Ｆ | PCV | 0.10 | yes | 3 |
| 245 | 73 | M | PCV | 0.70 | no | 2 |
| 246 | 66 | F | PCV | 0.70 | yes | 9 |
| 247 | 70 | M | PCV | 1.00 | no | 10 |
| 248 | 69 | M | PCV | 0.10 | no | 2 |
| 249 | 67 | M | PCV | 1.40 | yes | 15 |
| 250 | 77 | M | PCV | 0.05 | no | 1 |
| 251 | 74 | M | PCV | 0.22 | no | 7 |
| 252 | 73 | F | PCV | 0.52 | yes | 4 |
| 253 | 75 | M | PCV | 0.05 | yes | 1 |
| 254 | 71 | M | PCV | 1.00 | no | 5 |
| 255 | 83 | M | PCV | 1.05 | yes | 12 |
| 256 | 73 | M | PCV | 0.00 | no | 5 |
| 257 | 58 | M | PCV | 0.00 | yes | 2 |
| 258 | 71 | M | PCV | 1.15 | no | 6 |
| 259 | 83 | M | PCV | 0.70 | no | 3 |
| 260 | 56 | Ｆ | PCV | -0.18 | yes | 1 |
| 261 | 60 | Ｆ | RAP | 0.05 | yes | 4 |
| 262 | 75 | Ｆ | RAP | 0.00 | yes | 1 |
| 263 | 81 | Ｆ | RAP | 1.05 | yes | 4 |
| 264 | 82 | Ｆ | RAP | 1.10 | yes | 5 |
|  |  |  | RAP | 0.52 | yes | 3 |
| 265 | 80 | M | RAP | 1.22 | yes | 17 |
|  |  |  | RAP | 0.30 | yes | 2 |
| 266 | 80 | M | RAP | 1.00 | yes | 1 |
|  |  |  | RAP | 2.00 | yes | 11 |
| 267 | 61 | Ｆ | RAP | 0.00 | yes | 3 |
| 268 | 63 | Ｆ | RAP | 0.05 | no | 2 |
| 269 | 79 | Ｆ | RAP | 0.22 | yes | 2 |
| 270 | 87 | F | RAP | 0.70 | no | 30 |
|  |  |  | RAP | 2.00 | yes | 6 |
| 271 | 89 | Ｆ | RAP | 0.52 | yes | 7 |
| 272 | 76 | M | RAP | 0.70 | yes | 4 |
| 273 | 85 | M | RAP | 0.30 | yes | 3 |
| 274 | 75 | M | combined | 0.70 | no | NA |
|  |  |  | combined | -0.08 | no | NA |
| 275 | 76 | M | combined | 0.52 | yes | NA |
|  |  |  | combined | 1.15 | no | NA |
| 276 | 84 | M | combined | 1.70 | yes | NA |
|  |  |  | combined | 1.00 | no | NA |
| 277 | 79 | M | combined | 0.52 | yes | NA |
|  |  |  | combined | -0.08 | yes | NA |
| 278 | 75 | M | combined | 0.05 | no | NA |
|  |  |  | combined | 0.30 | no | NA |
| 279 | 53 | Ｆ | combined | -0.08 | yes | NA |
|  |  |  | combined | -0.18 | yes | NA |
| 280 | 81 | Ｆ | combined | 0.15 | no | NA |
|  |  |  | combined | 1.00 | yes | NA |
| 281 | 70 | M | combined | 1.70 | no | NA |
|  |  |  | combined | 0.82 | no | NA |
| 282 | 82 | M | combined | 0.15 | yes | NA |
|  |  |  | combined | 1.40 | no | NA |
| 283 | 82 | M | combined | 1.40 | no | NA |
|  |  |  | combined | 1.05 | no | NA |
| 284 | 75 | M | combined | 0.40 | no | NA |
|  |  |  | combined | 1.00 | yes | NA |
| 285 | 82 | M | combined | -0.08 | yes | NA |
|  |  |  | combined | 1.30 | yes | NA |
| 286 | 62 | M | combined | 0.30 | no | NA |
|  |  |  | combined | 0.30 | yes | NA |
| 287 | 68 | M | combined | 1.00 | yes | NA |
|  |  |  | combined | 0.10 | no | NA |
| 288 | 67 | F | combined | 1.00 | no | NA |
|  |  |  | combined | 1.22 | no | NA |
| 289 | 66 | F | combined | 0.00 | no | NA |
|  |  |  | combined | -0.08 | no | NA |
| Phase II |  |  |  |  |  |  |
| patient No. | age | sex | AMD Subtype | LogMAR BCVA | PED | Lesion size (DA) |
| 290 | 81 | M | tAMD | 0 | yes | 34.8 |
| 291 | 87 | F | tAMD | 1 | no | 11.7 |
| 292 | 78 | F | tAMD | 2 | no | 2.8 |
| 293 | 85 | F | tAMD | 1 | no | 0.9 |
| 294 | 79 | M | tAMD | 1 | yes | 7.1 |
| 295 | 77 | M | tAMD | 0 | yes | 15.1 |
| 296 | 69 | M | tAMD | 1 | no | 10.0 |
| 297 | 64 | M | tAMD | 0 | no | 4.4 |
|  |  |  | tAMD | 0 | no | 1.8 |
| 298 | 77 | M | tAMD | 1 | no | 2.4 |
| 299 | 82 | M | tAMD | 1 | no | 6.5 |
| 300 | 76 | M | tAMD | 2 | no | 7.4 |
| 301 | 80 | M | tAMD | 1 | no | 1.9 |
| 302 | 57 | F | tAMD | 1 | no | 0.4 |
| 303 | 80 | M | tAMD | 0 | no | 2.1 |
|  |  |  | tAMD | 1 | no | 2.2 |
| 304 | 82 | M | tAMD | 2 | no | 10.7 |
|  |  |  | tAMD | 1 | no | 10.9 |
| 305 | 77 | M | tAMD | 1 | no | 3.8 |
| 306 | 78 | M | tAMD | 1 | no | 5.6 |
| 307 | 86 | M | tAMD | 0 | no | 10.1 |
| 308 | 66 | F | tAMD | 1 | no | 3.5 |
| 309 | 72 | M | tAMD | 2 | no | 3.5 |
| 310 | 65 | M | tAMD | 0 | no | 1.5 |
| 311 | 73 | F | tAMD | 1 | no | 4.6 |
| 312 | 67 | F | tAMD | 1 | yes | 6.1 |
| 313 | 63 | M | tAMD | 0 | yes | 8.2 |
| 314 | 72 | M | tAMD | 1 | no | 5.7 |
| 315 | 70 | M | tAMD | 2 | no | 8.9 |
| 316 | 72 | M | tAMD | 2 | no | 20.4 |
|  |  |  | tAMD | 1 | yes | 10.2 |
| 317 | 76 | M | tAMD | 0 | no | 2.0 |
| 318 | 55 | F | tAMD | 1 | no | 2.2 |
| 319 | 78 | M | tAMD | 0 | no | 3.3 |
| 320 | 73 | M | tAMD | 1 | yes | 8.7 |
| 321 | 75 | F | tAMD | 0 | no | 1.9 |
| 322 | 63 | M | tAMD | 1 | no | 5.9 |
| 323 | 60 | M | tAMD | 0 | no | 0.8 |
| 324 | 83 | F | tAMD | 1 | no | 4.3 |
| 325 | 63 | M | tAMD | 0 | no | 1.2 |
| 326 | 74 | F | tAMD | 1 | no | 8.4 |
|  |  |  | tAMD | 1 | no | 11.9 |
| 327 | 80 | M | tAMD | 1 | no | 7.2 |
| 328 | 82 | M | tAMD | 1 | no | 2.5 |
| 329 | 73 | M | tAMD | 2 | no | 7.0 |
|  |  |  | tAMD | 1 | no | 11.2 |
| 330 | 60 | M | tAMD | 1 | no | 23.6 |
| 331 | 80 | M | tAMD | 1 | no | 2.0 |
| 332 | 83 | F | tAMD | 1 | no | 10.0 |
| 333 | 51 | F | tAMD | 1 | no | 1.8 |
|  |  |  | tAMD | 0 | no | 1.1 |
| 334 | 79 | M | tAMD | 2 | no | 2.3 |
| 335 | 86 | M | tAMD | 1 | no | 6.0 |
| 336 | 53 | M | tAMD | 0 | no | 3.3 |
| 337 | 75 | F | tAMD | 1 | no | 7.8 |
| 338 | 90 | M | tAMD | 2 | no | 6.9 |
| 339 | 77 | M | tAMD | 1 | yes | 5.4 |
| 340 | 83 | M | tAMD | 2 | no | 5.6 |
| 341 | 79 | F | tAMD | 1 | no | 1.4 |
| 342 | 51 | F | tAMD | 1 | no | 2.4 |
| 343 | 68 | M | tAMD | 0 | no | 2.8 |
| 344 | 72 | M | tAMD | 0 | yes | 3.0 |
| 345 | 87 | M | tAMD | 1 | no | 6.1 |
| 346 | 57 | F | tAMD | 0 | no | 0.8 |
| 347 | 72 | M | tAMD | 2 | yes | 35.9 |
| 348 | 56 | M | tAMD | 1 | no | 4.3 |
| 349 | 65 | M | tAMD | 1 | yes | 15.4 |
| 350 | 77 | M | tAMD | 0 | no | 2.6 |
| 351 | 88 | M | tAMD | 1 | no | 3.8 |
| 352 | 90 | M | tAMD | 1 | no | 17.1 |
|  |  |  | tAMD | 1 | no | 4.4 |
| 353 | 74 | M | tAMD | 0 | no | 1.9 |
| 354 | 78 | M | tAMD | 0 | no | 0.6 |
| 355 | 60 | F | tAMD | 0 | no | 0.3 |
| 356 | 76 | M | tAMD | 0 | yes | 4.5 |
| 357 | 86 | F | tAMD | 0 | yes | 4.9 |
| 358 | 76 | M | tAMD | 1 | no | 2.1 |
| 359 | 80 | M | tAMD | 1 | no | 7.4 |
| 360 | 81 | M | tAMD | 2 | no | 7.6 |
| 361 | 88 | M | tAMD | 1 | no | 12.4 |
| 362 | 70 | M | tAMD | 1 | no | 3.3 |
| 363 | 85 | F | tAMD | 1 | no | 2.3 |
| 364 | 58 | M | tAMD | 0 | no | 3.3 |
| 365 | 77 | M | tAMD | 2 | no | 11.5 |
| 366 | 66 | M | tAMD | 0 | no | 8.5 |
| 367 | 90 | M | tAMD | 1 | no | 3.5 |
| 368 | 78 | F | tAMD | 1 | no | 4.3 |
| 369 | 78 | M | tAMD | 1 | yes | 17.8 |
| 370 | 74 | M | tAMD | 0 | yes | 5.0 |
| 371 | 85 | M | tAMD | 1 | no | 8.0 |
| 372 | 73 | M | tAMD | 1 | no | 2.0 |
| 373 | 84 | M | tAMD | 0 | yes | 4.3 |
| 374 | 71 | M | tAMD | 0 | no | 0.9 |
| 375 | 82 | M | tAMD | 0 | no | 1.1 |
| 376 | 79 | F | tAMD | 1 | no | 4.0 |
| 377 | 78 | F | tAMD | 1 | no | 3.9 |
| 378 | 55 | F | tAMD | 1 | no | 0.9 |
| 379 | 68 | M | tAMD | 1 | yes | 18.0 |
| 380 | 54 | M | tAMD | 1 | no | 1.0 |
| 381 | 79 | M | tAMD | 0 | no | 2.1 |
| 382 | 85 | M | tAMD | 0 | no | 2.5 |
| 383 | 79 | M | tAMD | 1 | no | 12.0 |
| 384 | 72 | M | tAMD | 0 | no | 2.5 |
| 385 | 81 | M | tAMD | 1 | no | 4.5 |
| 386 | 66 | M | tAMD | 0 | no | 1.8 |
|  |  |  | tAMD | 1 | no | 1.5 |
| 387 | 71 | M | tAMD | 1 | no | 3.1 |
| 388 | 74 | F | tAMD | 0 | yes | 8.0 |
| 389 | 76 | M | tAMD | 1 | no | 3.1 |
| 390 | 76 | M | tAMD | 1 | no | 2.0 |
| 391 | 77 | M | tAMD | 0 | no | 1.9 |
| 392 | 62 | F | tAMD | 1 | no | 2.5 |
| 393 | 87 | F | tAMD | 0 | no | 2.9 |
| 394 | 80 | F | tAMD | 1 | no | 2.1 |
| 395 | 77 | M | tAMD | 1 | yes | 10.3 |
| 396 | 55 | F | tAMD | 0 | yes | 6.0 |
| 397 | 63 | F | tAMD | 0 | no | 2.5 |
| 398 | 74 | M | tAMD | 1 | no | 8.8 |
| 399 | 77 | M | tAMD | 1 | no | 4.4 |
| 400 | 81 | F | tAMD | 1 | no | 4.0 |
| 401 | 63 | M | tAMD | 1 | no | 0.5 |
| 402 | 70 | M | tAMD | 0 | no | 1.5 |
| 403 | 74 | M | tAMD | 1 | no | 2.8 |
| 404 | 77 | M | tAMD | 0 | no | 1.3 |
| 405 | 83 | M | PCV | 0 | yes | 7.4 |
|  |  |  | PCV | 1 | no | 19.6 |
| 406 | 67 | F | PCV | 1 | yes | 31.8 |
|  |  |  | PCV | 1 | no | 9.2 |
| 407 | 70 | M | PCV | 0 | no | 4.8 |
| 408 | 66 | M | PCV | 1 | yes | 9.4 |
| 409 | 62 | F | PCV | 0 | no | 2.2 |
|  |  |  | PCV | 0 | no | 2.2 |
| 410 | 52 | F | PCV | 0 | no | 3.9 |
| 411 | 78 | M | PCV | 0 | no | 1.6 |
| 412 | 75 | M | PCV | 2 | yes | 27.9 |
| 413 | 75 | M | PCV | 1 | no | 3.3 |
| 414 | 73 | F | PCV | 1 | yes | 5.3 |
| 415 | 70 | M | PCV | 0 | no | 2.8 |
| 416 | 83 | M | PCV | 1 | yes | 6.8 |
| 417 | 70 | M | PCV | 1 | no | 1.1 |
| 418 | 81 | M | PCV | 0 | no | 2.0 |
| 419 | 74 | F | PCV | 2 | no | 14.4 |
|  |  |  | PCV | 0 | yes | 7.9 |
| 420 | 66 | M | PCV | 1 | no | 2.3 |
| 421 | 70 | M | PCV | 1 | no | 0.5 |
| 422 | 84 | M | PCV | 1 | no | 13.2 |
|  |  |  | PCV | 1 | no | 26.7 |
| 423 | 73 | F | PCV | 1 | yes | 19.2 |
| 424 | 79 | M | PCV | 1 | no | 1.3 |
| 425 | 78 | M | PCV | 0 | yes | 3.4 |
| 426 | 71 | M | PCV | 1 | no | 1.6 |
| 427 | 76 | F | PCV | 1 | no | 9.5 |
| 428 | 79 | M | PCV | 1 | yes | 19.0 |
| 429 | 71 | M | PCV | 1 | yes | 18.6 |
| 430 | 72 | M | PCV | 1 | yes | 5.9 |
| 431 | 70 | M | PCV | 1 | no | 6.4 |
| 432 | 81 | M | PCV | 0 | no | 0.9 |
|  |  |  | PCV | 0 | no | 2.3 |
| 433 | 54 | F | PCV | 0 | no | 1.5 |
|  |  |  | PCV | 0 | no | 4.7 |
| 434 | 72 | M | PCV | 0 | no | 2.7 |
| 435 | 71 | F | PCV | 0 | no | 0.7 |
| 436 | 75 | M | PCV | 0 | no | 1.6 |
| 437 | 78 | M | PCV | 0 | no | 5.3 |
| 438 | 71 | F | PCV | 1 | yes | 10.3 |
| 439 | 71 | M | PCV | 0 | yes | 8.4 |
|  |  |  | PCV | 0 | no | 4.5 |
| 440 | 52 | F | PCV | 0 | yes | 15.9 |
| 441 | 71 | F | PCV | 2 | yes | 26.5 |
| 442 | 84 | M | PCV | 0 | no | 1.4 |
| 443 | 66 | M | PCV | 0 | yes | 43.9 |
| 444 | 79 | M | PCV | 1 | no | 5.9 |
| 445 | 74 | F | PCV | 1 | yes | 3.6 |
| 446 | 63 | M | PCV | 0 | no | 15.2 |
| 447 | 73 | M | PCV | 0 | no | 0.9 |
| 448 | 78 | F | PCV | 2 | no | 2.5 |
| 449 | 56 | M | PCV | 0 | no | 5.3 |
| 450 | 76 | M | PCV | 0 | no | 2.0 |
| 451 | 82 | M | PCV | 0 | no | 1.3 |
| 452 | 81 | F | PCV | 1 | no | 3.7 |
| 453 | 71 | M | PCV | 0 | yes | 2.2 |
|  |  |  | PCV | 1 | no | 4.6 |
| 454 | 70 | F | PCV | 1 | no | 1.9 |
| 455 | 92 | F | PCV | 0 | no | 0.9 |
|  |  |  | PCV | 2 | no | 1.1 |
| 456 | 82 | M | PCV | 0 | yes | 10.8 |
|  |  |  | PCV | 0 | no | 0.3 |
| 457 | 55 | F | PCV | 0 | no | 1.7 |
| 458 | 81 | M | PCV | 0 | yes | 1.3 |
|  |  |  | PCV | 1 | no | 6.2 |
| 459 | 80 | M | PCV | 0 | no | 25.1 |
|  |  |  | PCV | 1 | yes | 16.8 |
| 460 | 73 | M | PCV | 1 | no | 1.7 |
| 461 | 68 | M | PCV | 1 | no | 2.5 |
| 462 | 66 | M | PCV | 1 | no | 3.9 |
| 463 | 68 | M | PCV | 1 | yes | 5.5 |
| 464 | 68 | M | PCV | 0 | no | 2.9 |
| 465 | 70 | M | PCV | 1 | no | 3.5 |
| 466 | 74 | M | PCV | 1 | yes | 21.6 |
| 467 | 68 | F | PCV | 1 | no | 17.1 |
|  |  |  | PCV | 1 | no | 5.1 |
| 468 | 76 | M | PCV | 1 | no | 3.4 |
| 469 | 80 | M | PCV | 1 | no | 5.9 |
| 470 | 86 | F | PCV | 2 | no | 9.6 |
| 471 | 69 | M | PCV | 0 | no | 1.0 |
| 472 | 50 | M | PCV | 1 | no | 11.3 |
| 473 | 71 | M | PCV | 1 | yes | 38.5 |
| 474 | 79 | M | PCV | 1 | no | 7.5 |
| 475 | 75 | M | PCV | 0 | no | 2.9 |
| 476 | 77 | F | PCV | 1 | no | 3.5 |
| 477 | 88 | M | PCV | 1 | no | 5.4 |
| 478 | 75 | F | PCV | 1 | yes | 3.0 |
| 479 | 77 | M | PCV | 1 | yes | 5.9 |
| 480 | 70 | M | PCV | 0 | yes | 2.5 |
| 481 | 72 | M | PCV | 1 | no | 2.8 |
| 482 | 66 | M | PCV | 0 | no | 0.8 |
| 483 | 73 | F | PCV | 0 | yes | 0.8 |
|  |  |  | PCV | 0 | no | 3.8 |
| 484 | 66 | M | PCV | 0 | no | 1.0 |
| 485 | 76 | M | PCV | 1 | no | 3.5 |
| 486 | 76 | M | PCV | 1 | no | 1.3 |
| 487 | 63 | M | PCV | 0 | yes | 11.1 |
| 488 | 77 | M | PCV | 1 | no | 5.2 |
| 489 | 79 | F | PCV | 1 | no | 6.9 |
| 490 | 83 | M | PCV | 1 | yes | 7.7 |
| 491 | 87 | M | PCV | 1 | yes | 11.2 |
| 492 | 75 | F | PCV | 0 | yes | 4.3 |
|  |  |  | PCV | 0 | no | 3.3 |
| 493 | 65 | F | PCV | 0 | yes | 10.0 |
| 494 | 79 | M | PCV | 0 | no | 5.8 |
| 495 | 81 | M | PCV | 0 | no | 1.1 |
| 496 | 62 | M | PCV | 0 | no | 1.6 |
| 497 | 80 | M | PCV | 2 | yes | 39.5 |
| 498 | 76 | M | PCV | 1 | no | 5.8 |
| 499 | 72 | M | PCV | 0 | yes | 9.4 |
| 500 | 66 | F | PCV | 1 | no | 0.5 |
| 501 | 78 | F | PCV | 1 | no | 10.8 |
| 502 | 85 | M | PCV | 0 | no | 3.6 |
| 503 | 70 | M | PCV | 1 | no | 2.4 |
| 504 | 65 | F | PCV | 0 | no | 2.6 |
| 505 | 62 | M | PCV | 1 | yes | 1.7 |
| 506 | 65 | M | PCV | 1 | no | 2.1 |
| 507 | 78 | M | PCV | 1 | yes | 13.1 |
| 508 | 72 | M | PCV | 0 | no | 0.8 |
| 509 | 66 | M | PCV | 0 | no | 6.0 |
| 510 | 74 | M | PCV | 1 | no | 2.0 |
| 511 | 71 | F | PCV | 1 | no | 13.0 |
| 512 | 78 | M | PCV | 0 | yes | 2.8 |
| 513 | 69 | M | PCV | 1 | no | 8.4 |
| 514 | 62 | M | PCV | 0 | no | 1.7 |
| 515 | 67 | F | PCV | 1 | no | 2.7 |
| 516 | 76 | F | PCV | 0 | no | 8.5 |
| 517 | 78 | F | PCV | 0 | yes | 8.1 |
| 518 | 70 | F | PCV | 0 | yes | 17.8 |
|  |  |  | PCV | 0 | no | 1.3 |
| 519 | 64 | F | PCV | 1 | no | 6.3 |
| 520 | 54 | F | PCV | 1 | yes | 4.9 |
| 521 | 68 | M | PCV | 0 | no | 2.1 |
| 522 | 74 | F | PCV | 0 | no | 1.3 |
| 523 | 70 | M | PCV | 0 | no | 2.2 |
| 524 | 61 | M | PCV | 0 | no | 0.8 |
| 525 | 74 | M | PCV | 0 | no | 1.0 |
| 526 | 72 | M | PCV | 0 | no | 0.5 |
| 527 | 67 | F | PCV | 1 | no | 29.8 |
| 528 | 70 | M | PCV | 0 | yes | 39.8 |
| 529 | 74 | M | PCV | 1 | no | 1.3 |
| 530 | 82 | M | PCV | 1 | no | 0.6 |
| 531 | 75 | F | PCV | 1 | no | 3.3 |
| 532 | 67 | F | PCV | 1 | no | 6.6 |
| 533 | 75 | M | PCV | 1 | no | 40.3 |
| 534 | 76 | M | PCV | 1 | yes | 19.9 |
| 535 | 77 | M | PCV | 2 | no | 35.8 |
| 536 | 89 | F | RAP | 1 | no | 2.5 |
|  |  |  | RAP | 0 | no | 2.3 |
| 537 | 75 | F | RAP | 0 | no | 2.3 |
|  |  |  | RAP | 1 | no | 3.0 |
| 538 | 64 | M | RAP | 0 | yes | 3.9 |
|  |  |  | RAP | 1 | no | 3.0 |
| 539 | 84 | M | RAP | 0 | yes | 1.7 |
|  |  |  | RAP | 1 | yes | 13.7 |
| 540 | 76 | F | RAP | 0 | no | 4.9 |
|  |  |  | RAP | 0 | no | 5.6 |
| 541 | 86 | F | RAP | 1 | yes | 4.4 |
| 542 | 77 | M | RAP | 0 | no | 3.8 |
| 543 | 90 | M | RAP | 0 | yes | 6.3 |
|  |  |  | RAP | 1 | yes | 3.4 |
| 544 | 65 | F | RAP | 1 | yes | 9.7 |
|  |  |  | RAP | 0 | no | 0.5 |
| 545 | 91 | F | RAP | 1 | no | 2.7 |
|  |  |  | RAP | 0 | yes | 2.7 |
| 546 | 77 | M | RAP | 0 | no | 1.7 |
| 547 | 63 | M | RAP | 1 | yes | 4.3 |
| 548 | 77 | M | combined | 1 | no | NA |
|  |  |  | combined | 1 | no | NA |
| 549 | 83 | M | combined | 0 | no | NA |
|  |  |  | combined | 1 | no | NA |
| 550 | 78 | M | combined | 1 | yes | NA |
|  |  |  | combined | 1 | no | NA |
| 551 | 80 | M | combined | 1 | yes | NA |
|  |  |  | combined | 0 | no | NA |
| 552 | 65 | M | combined | 0 | no | NA |
|  |  |  | combined | 1 | no | NA |
| 553 | 70 | F | combined | 0 | no | NA |
|  |  |  | combined | 1 | no | NA |
| 554 | 80 | M | combined | 1 | no | NA |
|  |  |  | combined | 0 | no | NA |
| 555 | 76 | F | combined | 1 | no | NA |
|  |  |  | combined | 0 | yes | NA |
| 556 | 63 | M | combined | 0 | no | NA |
|  |  |  | combined | 0 | yes | NA |
| 557 | 77 | M | combined | 3 | no | NA |
|  |  |  | combined | 1 | yes | NA |
| 558 | 81 | F | combined | 0 | no | NA |
|  |  |  | combined | 2 | no | NA |
| 559 | 80 | F | combined | 0 | no | NA |
|  |  |  | combined | 2 | no | NA |
| 560 | 77 | M | combined | 1 | no | NA |
|  |  |  | combined | 1 | yes | NA |
| 561 | 78 | M | combined | 1 | yes | NA |
|  |  |  | combined | 2 | no | NA |
| Phase III |  |  |  |  |  |  |
| patient No. | age | sex | AMD Subtype | LogMAR BCVA | PED | Lesion size (DA) |
| 562 | 71 | M | tAMD | 1 | no | 6.1 |
| 563 | 60 | M | tAMD | 0 | no | 11.7 |
| 564 | 78 | M | tAMD | 0 | no | 1.3 |
| 565 | 71 | M | tAMD | 0 | no | 5.2 |
| 566 | 52 | M | tAMD | 1 | no | 1.1 |
| 567 | 73 | F | tAMD | 0 | no | 5.3 |
| 568 | 57 | M | tAMD | 0 | no | 0.9 |
| 569 | 74 | M | tAMD | 0 | no | 5.5 |
| 570 | 79 | F | tAMD | 1 | no | 4.1 |
| 571 | 66 | F | tAMD | 0 | no | 2.2 |
| 572 | 69 | F | tAMD | 0 | no | 0.9 |
| 573 | 72 | M | tAMD | 1 | no | 8.2 |
| 574 | 85 | F | tAMD | 1 | no | 3.4 |
| 575 | 81 | M | tAMD | 0 | no | 0.8 |
| 576 | 63 | M | tAMD | 0 | no | 1.1 |
| 577 | 84 | M | tAMD | 1 | no | 2.0 |
| 578 | 82 | M | tAMD | 1 | no | 20.0 |
| 579 | 68 | M | tAMD | 1 | no | 2.8 |
| 580 | 82 | M | tAMD | 0 | yes | 4.4 |
| 581 | 54 | F | tAMD | 0 | no | 2.5 |
| 582 | 79 | M | tAMD | 0 | no | 4.4 |
| 583 | 71 | M | tAMD | 1 | no | 2.4 |
| 584 | 60 | M | tAMD | 1 | no | 3.7 |
| 585 | 83 | F | tAMD | 1 | yes | 3.5 |
| 586 | 71 | M | tAMD | 0 | no | 4.8 |
| 587 | 89 | M | tAMD | 1 | no | 2.7 |
| 588 | 64 | M | tAMD | 1 | no | 10.5 |
| 589 | 73 | F | tAMD | 1 | no | 1.0 |
| 590 | 62 | M | tAMD | 0 | no | 1.3 |
| 591 | 80 | F | tAMD | 1 | no | 4.3 |
| 592 | 73 | M | tAMD | 1 | yes | 10.4 |
| 593 | 57 | M | tAMD | 0 | no | 3.2 |
| 594 | 81 | M | tAMD | 1 | yes | 9.6 |
|  |  |  | tAMD | 2 | no | 8.5 |
| 595 | 69 | M | tAMD | 1 | no | 3.2 |
| 596 | 75 | M | tAMD | 0 | no | 1.6 |
| 597 | 87 | M | tAMD | 0 | yes | 3.3 |
| 598 | 55 | M | tAMD | 0 | no | 1.6 |
| 599 | 51 | M | tAMD | 0 | no | 0.3 |
| 600 | 76 | M | tAMD | 1 | no | 3.1 |
| 601 | 79 | F | tAMD | 1 | no | 6.2 |
| 602 | 73 | M | tAMD | 1 | no | 3.5 |
| 603 | 58 | M | tAMD | 1 | no | 2.3 |
| 604 | 72 | F | tAMD | 0 | yes | 4,2 |
| 605 | 82 | M | tAMD | 0 | yes | 3.2 |
| 606 | 79 | M | tAMD | 1 | no | 4.9 |
| 607 | 65 | F | tAMD | 1 | no | 4.7 |
| 608 | 63 | F | tAMD | 1 | no | 1.5 |
| 609 | 72 | M | tAMD | 1 | no | 1.7 |
| 610 | 82 | F | tAMD | 1 | no | 15.7 |
|  |  |  | tAMD | 1 | no | 5.8 |
| 611 | 84 | M | tAMD | 1 | no | 6.7 |
| 612 | 48 | M | tAMD | 1 | no | 4.2 |
| 613 | 79 | M | tAMD | 0 | yes | 14.0 |
| 614 | 77 | M | tAMD | 0 | no | 3.9 |
| 615 | 64 | M | tAMD | 0 | no | 1.2 |
| 616 | 77 | M | tAMD | 1 | no | 5.3 |
| 617 | 75 | M | tAMD | 0 | yes | 3.3 |
| 618 | 77 | M | tAMD | 1 | no | 3.2 |
| 619 | 78 | F | tAMD | 1 | no | 5.6 |
| 620 | 70 | F | tAMD | 1 | no | 2.0 |
| 621 | 87 | M | tAMD | 1 | no | 2.9 |
| 622 | 77 | M | tAMD | 1 | no | 4.0 |
| 623 | 71 | F | tAMD | 1 | no | 3.6 |
| 624 | 82 | M | tAMD | 1 | no | 15.1 |
|  |  |  | tAMD | 1 | no | 17.6 |
| 625 | 84 | M | tAMD | 2 | no | 16.7 |
|  |  |  | tAMD | 1 | no | 16.8 |
| 626 | 78 | M | tAMD | 0 | no | 13.2 |
|  |  |  | tAMD | 1 | no | 12.7 |
| 627 | 69 | M | tAMD | 1 | yes | 8.1 |
| 628 | 70 | M | tAMD | 0 | no | 0.6 |
| 629 | 65 | M | tAMD | 0 | yes | 1.4 |
| 630 | 75 | M | tAMD | 0 | yes | 10.3 |
| 631 | 78 | M | tAMD | 2 | no | 1.9 |
| 632 | 77 | M | tAMD | 1 | no | 4.5 |
| 633 | 71 | M | tAMD | 0 | no | 1.1 |
| 634 | 50 | M | tAMD | 0 | no | 2.2 |
| 635 | 77 | M | tAMD | 0 | yes | 2.8 |
| 636 | 82 | M | tAMD | 0 | no | 2.8 |
| 637 | 78 | M | tAMD | 0 | no | 0.8 |
|  |  |  | tAMD | 0 | no | 0.2 |
| 638 | 82 | M | tAMD | 1 | yes | 11.3 |
| 639 | 64 | M | tAMD | 0 | no | 5.3 |
| 640 | 81 | M | tAMD | 0 | no | 4.3 |
| 641 | 59 | F | tAMD | 1 | yes | 10.3 |
| 642 | 73 | M | tAMD | 1 | yes | 6.4 |
| 643 | 76 | M | tAMD | 1 | no | 4.2 |
| 644 | 54 | M | tAMD | 1 | no | 5.2 |
| 645 | 76 | M | tAMD | 0 | yes | 18.5 |
| 646 | 82 | F | tAMD | 1 | no | 4.5 |
| 647 | 70 | M | tAMD | 1 | no | 7.7 |
| 648 | 77 | M | tAMD | 0 | yes | 8.3 |
| 649 | 74 | M | tAMD | 0 | no | 2.7 |
| 650 | 59 | M | tAMD | 1 | no | 2.2 |
| 651 | 85 | M | tAMD | 1 | no | 2.5 |
| 652 | 76 | M | tAMD | 1 | yes | 4.1 |
| 653 | 81 | M | tAMD | 1 | yes | 4.9 |
|  |  |  | tAMD | 1 | no | 7.7 |
| 654 | 75 | F | tAMD | 0 | no | 2.1 |
| 655 | 74 | M | tAMD | 0 | no | 1.4 |
| 656 | 79 | M | tAMD | 1 | no | 7.5 |
| 657 | 75 | M | tAMD | 1 | no | 3.2 |
| 658 | 79 | F | tAMD | 0 | no | 3.9 |
| 659 | 76 | M | tAMD | 1 | no | 2.3 |
| 660 | 76 | M | tAMD | 0 | no | 2.7 |
|  |  |  | tAMD | 0 | no | 3.4 |
| 661 | 74 | M | tAMD | 1 | no | 1.6 |
| 662 | 88 | M | tAMD | 1 | no | 1.3 |
| 663 | 77 | M | tAMD | 0 | yes | 4.3 |
| 664 | 73 | M | tAMD | 1 | no | 2.5 |
| 665 | 74 | M | tAMD | 0 | yes | 10.2 |
| 666 | 61 | M | tAMD | 0 | yes | 6.4 |
| 667 | 66 | M | tAMD | 0 | no | 5.2 |
| 668 | 85 | M | tAMD | 0 | no | 9.2 |
| 669 | 62 | F | tAMD | 0 | no | 4.2 |
| 670 | 76 | M | tAMD | 1 | no | 1.9 |
| 671 | 72 | F | tAMD | 0 | no | 0.8 |
| 672 | 83 | M | tAMD | 1 | no | 3.2 |
| 673 | 73 | F | tAMD | 0 | no | 4.7 |
| 674 | 66 | M | tAMD | 0 | no | 9.2 |
| 675 | 79 | M | tAMD | 0 | no | 2.0 |
| 676 | 83 | F | tAMD | 1 | no | 1.4 |
| 677 | 47 | M | tAMD | 0 | yes | 4.0 |
| 678 | 67 | M | tAMD | 0 | no | 1.7 |
| 679 | 62 | M | tAMD | 1 | no | 3.7 |
| 680 | 68 | M | tAMD | 0 | no | 0.3 |
|  |  |  | tAMD | 0 | no | 2.3 |
| 681 | 88 | M | tAMD | 1 | no | 7.9 |
| 682 | 76 | M | tAMD | 1 | no | 0.6 |
| 683 | 76 | M | tAMD | 0 | no | 6.2 |
| 684 | 73 | M | tAMD | 0 | no | 1.5 |
| 685 | 84 | M | tAMD | 0 | no | 4.3 |
| 686 | 81 | M | tAMD | 1 | no | 1.8 |
| 687 | 76 | M | tAMD | 1 | no | 3.6 |
| 688 | 56 | M | tAMD | 0 | no | 1.8 |
| 689 | 80 | M | tAMD | 0 | no | 2.4 |
|  |  |  | tAMD | 0 | no | 7.5 |
| 690 | 78 | M | tAMD | 1 | no | 5.3 |
| 691 | 75 | F | tAMD | 1 | no | 3.0 |
|  |  |  | tAMD | 1 | no | 2.6 |
| 692 | 78 | M | tAMD | 1 | yes | 18.5 |
| 693 | 94 | M | tAMD | 0 | yes | 15.3 |
| 694 | 69 | M | tAMD | 0 | yes | 2.7 |
| 695 | 79 | M | tAMD | 1 | no | 7.4 |
| 696 | 73 | M | tAMD | 0 | no | 1.6 |
| 697 | 59 | M | tAMD | 0 | no | 0.7 |
| 698 | 76 | M | tAMD | 1 | no | 2.5 |
| 699 | 71 | M | tAMD | 0 | no | 0.5 |
| 700 | 66 | M | tAMD | 0 | no | 1.0 |
|  |  |  | tAMD | 0 | no | 2.3 |
| 701 | 79 | M | tAMD | 0 | yes | 7.3 |
| 702 | 88 | M | tAMD | 1 | no | 1.5 |
| 703 | 71 | M | tAMD | 0 | no | 1.8 |
| 704 | 77 | F | tAMD | 1 | no | 3.7 |
| 705 | 74 | M | tAMD | 0 | yes | 3.3 |
| 706 | 63 | M | tAMD | 0 | no | 0.9 |
| 707 | 85 | M | tAMD | 0 | no | 10.2 |
| 708 | 83 | M | tAMD | 1 | no | 0.6 |
| 709 | 85 | F | tAMD | 1 | no | 6.9 |
| 710 | 81 | F | tAMD | 1 | no | 3.5 |
| 711 | 78 | M | tAMD | 0 | no | 0.9 |
| 712 | 75 | F | tAMD | 0 | no | 2.4 |
| 713 | 87 | F | tAMD | 0 | no | 3.2 |
|  |  |  | tAMD | 1 | no | 9.1 |
| 714 | 81 | M | tAMD | 0 | no | 1.8 |
| 715 | 84 | M | tAMD | 0 | no | 0.6 |
| 716 | 53 | M | tAMD | 0 | no | 2.2 |
| 717 | 75 | M | tAMD | 0 | no | 1.0 |
| 718 | 80 | M | tAMD | 0 | no | 5.7 |
|  |  |  | tAMD | 0 | no | 5.0 |
| 719 | 78 | M | tAMD | 2 | no | 9.2 |
| 720 | 71 | M | tAMD | 0 | no | 1.2 |
|  |  |  | tAMD | 0 | yes | 3.4 |
| 721 | 58 | M | tAMD | 1 | no | 8.7 |
| 722 | 82 | M | tAMD | 0 | yes | 5.7 |
| 723 | 90 | M | tAMD | 2 | no | 4.8 |
| 724 | 64 | M | tAMD | 1 | yes | 9.8 |
| 725 | 89 | M | tAMD | 1 | no | 3.4 |
| 726 | 60 | F | tAMD | 0 | no | 2.4 |
|  |  |  | tAMD | 1 | no | 2.0 |
| 727 | 55 | M | tAMD | 1 | no | 0.4 |
| 728 | 50 | M | tAMD | 0 | no | 0.7 |
| 729 | 56 | M | tAMD | 0 | no | 0.8 |
| 730 | 78 | F | tAMD | 1 | no | 4.0 |
| 731 | 65 | M | tAMD | 1 | no | 4.5 |
| 732 | 73 | M | tAMD | 1 | no | 0.6 |
| 733 | 81 | M | tAMD | 1 | no | 1.7 |
| 734 | 77 | M | PCV | 1 | no | 2.1 |
| 735 | 84 | F | PCV | 2 | yes | 30.0 |
|  |  |  | PCV | 1 | no | 30.5 |
| 736 | 80 | M | PCV | 0 | no | 3.6 |
| 737 | 67 | M | PCV | 1 | no | 4.9 |
| 738 | 73 | M | PCV | 0 | no | 4.6 |
| 739 | 80 | M | PCV | 0 | no | 4.8 |
| 740 | 74 | M | PCV | 1 | no | 10.2 |
| 741 | 63 | F | PCV | 0 | yes | 3.7 |
| 742 | 57 | M | PCV | 0 | no | 0.8 |
| 743 | 79 | M | PCV | 0 | yes | 3.1 |
| 744 | 76 | M | PCV | 1 | no | 11.3 |
| 745 | 71 | F | PCV | 0 | yes | 6.5 |
| 746 | 83 | F | PCV | 0 | no | 1.3 |
| 747 | 72 | F | PCV | 0 | no | 0.5 |
| 748 | 77 | M | PCV | 1 | yes | 4.2 |
| 749 | 77 | M | PCV | 1 | no | 6.2 |
| 750 | 75 | M | PCV | 0 | no | 0.6 |
| 751 | 77 | M | PCV | 0 | no | 2.3 |
| 752 | 78 | M | PCV | 0 | yes | 30.0 |
| 753 | 66 | M | PCV | 1 | no | 6.0 |
| 754 | 74 | M | PCV | 1 | no | 2.4 |
| 755 | 77 | M | PCV | 1 | no | 1.7 |
| 756 | 79 | M | PCV | 1 | no | 1.4 |
| 757 | 60 | M | PCV | 0 | no | 2.8 |
| 758 | 71 | F | PCV | 0 | no | 1.5 |
| 759 | 70 | F | PCV | 0 | no | 3.1 |
|  |  |  | PCV | 0 | no | 6.6 |
| 760 | 56 | M | PCV | 0 | no | 1.3 |
| 761 | 67 | F | PCV | 1 | no | 2.2 |
| 762 | 75 | F | PCV | 0 | no | 0.9 |
| 763 | 76 | M | PCV | 1 | yes | 16.8 |
| 764 | 81 | M | PCV | 1 | no | 2.6 |
| 765 | 55 | F | PCV | 0 | no | 0.5 |
|  |  |  | PCV | 1 | no | 1.7 |
| 766 | 86 | F | PCV | 1 | no | 12.3 |
| 767 | 64 | M | PCV | 0 | no | 5.1 |
| 768 | 75 | M | PCV | 1 | no | 2.0 |
| 769 | 81 | M | PCV | 1 | yes | 11.5 |
|  |  |  | PCV | 1 | yes | 11.8 |
| 770 | 77 | M | PCV | 0 | no | 0.8 |
| 771 | 79 | M | PCV | 0 | yes | 2.7 |
| 772 | 78 | M | PCV | 1 | no | 4.1 |
| 773 | 71 | M | PCV | 0 | no | 1.9 |
| 774 | 70 | M | PCV | 1 | no | 1.4 |
| 775 | 86 | M | PCV | 0 | no | 0.5 |
| 776 | 63 | M | PCV | 0 | no | 0.9 |
| 777 | 72 | F | PCV | 2 | no | 17.6 |
| 778 | 80 | M | PCV | 0 | no | 3.8 |
| 779 | 46 | M | PCV | 1 | yes | 39.9 |
| 780 | 83 | M | PCV | 0 | no | 3.8 |
|  |  |  | PCV | 0 | no | 1.3 |
| 781 | 74 | F | PCV | 0 | no | 4.7 |
| 782 | 73 | M | PCV | 1 | yes | 10.8 |
| 783 | 59 | F | PCV | 0 | yes | 1.1 |
| 784 | 74 | M | PCV | 0 | yes | 5.7 |
| 785 | 59 | M | PCV | 0 | yes | 8.5 |
| 786 | 78 | M | PCV | 1 | no | 5.0 |
| 787 | 73 | M | PCV | 0 | yes | 4.1 |
| 788 | 79 | F | PCV | 1 | yes | 15.2 |
| 789 | 77 | M | PCV | 1 | no | 0.9 |
| 790 | 76 | M | PCV | 0 | yes | 10.5 |
| 791 | 69 | F | PCV | 0 | no | 0.8 |
| 792 | 74 | M | PCV | 0 | no | 5.7 |
| 793 | 64 | M | PCV | 1 | no | 4.9 |
| 794 | 70 | M | PCV | 1 | no | 16.6 |
|  |  |  | PCV | 0 | no | 10.6 |
| 795 | 77 | F | PCV | 0 | no | 4.0 |
| 796 | 85 | M | PCV | 0 | yes | 15.6 |
|  |  |  | PCV | 1 | no | 14.4 |
| 797 | 68 | M | PCV | 0 | yes | 9.9 |
|  |  |  | PCV | 0 | no | 6.3 |
| 798 | 76 | M | PCV | 0 | no | 2.6 |
| 799 | 77 | F | PCV | 1 | yes | 11.1 |
| 800 | 70 | M | PCV | 0 | yes | 1.5 |
| 801 | 84 | M | PCV | 1 | yes | 4.9 |
| 802 | 73 | M | PCV | 0 | yes | 1.1 |
| 803 | 82 | M | PCV | 0 | yes | 2.4 |
|  |  |  | PCV | 0 | no | 6.3 |
| 804 | 74 | M | PCV | 0 | yes | 8.4 |
| 805 | 77 | M | PCV | 1 | yes | 15.9 |
| 806 | 72 | M | PCV | 1 | no | 9.1 |
|  |  |  | PCV | 1 | no | 10.3 |
| 807 | 55 | M | PCV | 1 | no | 3.2 |
| 808 | 74 | M | PCV | 0 | no | 0.3 |
| 809 | 77 | M | PCV | 1 | no | 4.1 |
| 810 | 78 | M | PCV | 0 | no | 0.9 |
| 811 | 70 | M | PCV | 0 | yes | 0.3 |
| 812 | 74 | F | PCV | 1 | no | 3.8 |
| 813 | 65 | M | PCV | 0 | no | 0.8 |
| 814 | 80 | M | PCV | 0 | no | 9.7 |
| 815 | 76 | M | PCV | 0 | no | 11.9 |
| 816 | 68 | M | PCV | 1 | no | 6.4 |
| 817 | 79 | M | PCV | 1 | no | 1.4 |
| 818 | 76 | M | PCV | 0 | no | 0.5 |
| 819 | 73 | M | PCV | 1 | no | 0.4 |
| 820 | 72 | M | PCV | 0 | no | 0.8 |
| 821 | 70 | M | PCV | 0 | no | 3.5 |
| 822 | 79 | M | PCV | 0 | yes | 10.3 |
| 823 | 63 | M | PCV | 1 | no | 1.3 |
| 824 | 75 | M | PCV | 0 | no | 1.9 |
|  |  |  | PCV | 0 | no | 0.2 |
| 825 | 80 | M | PCV | 0 | no | 2.1 |
| 826 | 76 | M | PCV | 1 | yes | 12.6 |
| 827 | 84 | M | PCV | 0 | no | 1.5 |
| 828 | 60 | M | PCV | 0 | no | 0.7 |
| 829 | 77 | F | PCV | 0 | no | 0.7 |
| 830 | 52 | M | PCV | 1 | yes | 13.3 |
| 831 | 75 | M | PCV | 0 | no | 4.1 |
| 832 | 75 | M | PCV | 0 | no | 2.8 |
| 833 | 81 | M | PCV | 0 | no | 3.1 |
| 834 | 75 | F | PCV | 0 | no | 0.8 |
| 835 | 68 | M | PCV | 1 | no | 1.3 |
| 836 | 85 | M | PCV | 0 | no | 1.0 |
| 837 | 73 | M | PCV | 1 | no | 1.5 |
| 838 | 75 | M | PCV | 0 | no | 0.7 |
| 839 | 78 | M | PCV | 1 | yes | 32.7 |
| 840 | 72 | M | PCV | 1 | no | 11.9 |
| 841 | 70 | M | PCV | 0 | no | 1.6 |
| 842 | 79 | F | PCV | 1 | no | 11.2 |
|  |  |  | PCV | 1 | no | 22.9 |
| 843 | 59 | M | PCV | 0 | yes | 0.3 |
| 844 | 69 | F | PCV | 1 | yes | 29.4 |
| 845 | 66 | M | PCV | 1 | no | 1.0 |
| 846 | 79 | F | PCV | 0 | yes | 0.8 |
| 847 | 64 | M | PCV | 1 | no | 5.1 |
| 848 | 75 | M | PCV | 1 | yes | 3.0 |
| 849 | 66 | M | PCV | 1 | no | 21.2 |
| 850 | 73 | M | PCV | 1 | no | 4.3 |
| 851 | 74 | M | PCV | 1 | no | 2.5 |
| 852 | 81 | M | PCV | 0 | no | 4.1 |
| 853 | 75 | M | PCV | 0 | no | 0.5 |
| 854 | 80 | M | PCV | 1 | no | 5.4 |
| 855 | 70 | M | PCV | 0 | no | 4.6 |
| 856 | 58 | M | PCV | 0 | yes | 2.2 |
| 857 | 77 | F | PCV | 0 | no | 1.5 |
| 858 | 64 | M | PCV | 0 | no | 3.6 |
| 859 | 79 | M | PCV | 1 | no | 2.2 |
| 860 | 68 | F | PCV | 0 | no | 1.4 |
| 861 | 76 | F | PCV | 1 | yes | 9.8 |
| 862 | 58 | M | PCV | 1 | no | 25.0 |
|  |  |  | PCV | 0 | no | 0.3 |
| 863 | 64 | M | PCV | 0 | no | 0.8 |
| 864 | 88 | F | RAP | 0 | no | 1.8 |
| 865 | 89 | M | RAP | 0 | yes | 2.8 |
| 866 | 87 | M | RAP | 0 | yes | 10.0 |
| 867 | 90 | F | RAP | 1 | yes | 14.6 |
| 868 | 77 | M | RAP | 3 | no | 20.0 |
|  |  |  | RAP | 1 | yes | 2.6 |
| 869 | 91 | M | RAP | 0 | no | 9.0 |
|  |  |  | RAP | 1 | no | 0.3 |
| 870 | 88 | F | RAP | 1 | yes | 5.3 |
|  |  |  | RAP | 0 | yes | 4.0 |
| 871 | 84 | F | RAP | 1 | no | 7.2 |
|  |  |  | RAP | 1 | yes | 9.5 |
| 872 | 75 | M | RAP | 0 | no | 0.7 |
| 873 | 88 | M | RAP | 0 | no | 0.5 |
| 874 | 92 | M | RAP | 1 | yes | 3.0 |
| 875 | 73 | F | RAP | 1 | no | 7.2 |
| 876 | 85 | F | RAP | 1 | yes | 5.2 |
| 877 | 82 | M | RAP | 1 | no | 13.9 |
| 878 | 83 | M | RAP | 1 | yes | 5.3 |
|  |  |  | RAP | 0 | no | 4.7 |
| 879 | 82 | M | combined | 0 | no | NA |
|  |  |  | combined | 1 | no | NA |
| 880 | 79 | M | combined | 2 | no | NA |
|  |  |  | combined | 0 | no | NA |
| 881 | 85 | M | combined | 1 | no | NA |
|  |  |  | combined | 4 | no | NA |
| 882 | 54 | M | combined | 0 | no | NA |
|  |  |  | combined | 0 | no | NA |
| 883 | 80 | M | combined | 1 | no | NA |
|  |  |  | combined | 1 | no | NA |
| 884 | 75 | M | combined | 0 | no | NA |
|  |  |  | combined | 1 | no | NA |
| 885 | 85 | M | combined | 0 | yes | NA |
|  |  |  | combined | 2 | no | NA |
| Phase IV |  |  |  |  |  |  |
| patient No. | age | sex | AMD Subtype | LogMAR BCVA | PED | Lesion size (DA) |
| 886 | 80 | F | tAMD | 0 | yes | 2.6 |
|  |  |  | tAMD | 0 | no | 6.0 |
| 887 | 78 | M | tAMD | 1 | no | 4.3 |
| 888 | 74 | M | tAMD | 1 | no | 6.2 |
| 889 | 74 | Ｍ | tAMD | 1 | no | 8.8 |
| 890 | 76 | M | tAMD | 0 | yes | 8.1 |
| 891 | 74 | F | tAMD | 0 | no | 2.8 |
| 892 | 73 | M | tAMD | 0 | no | 2.1 |
| 893 | 67 | M | tAMD | 0 | yes | 9.5 |
| 894 | 74 | M | tAMD | 0 | yes | 6.0 |
| 895 | 59 | M | tAMD | 0 | no | 4.2 |
| 896 | 90 | M | tAMD | 1 | yes | 7.1 |
| 897 | 81 | M | tAMD | 0 | yes | 7.4 |
| 898 | 75 | M | tAMD | 1 | no | 4.8 |
| 899 | 81 | M | tAMD | 0 | no | 3.2 |
| 900 | 74 | M | tAMD | 1 | no | 1.0 |
| 901 | 71 | M | tAMD | 1 | no | 2.2 |
| 902 | 82 | M | tAMD | 1 | yes | 3.5 |
| 903 | 82 | F | tAMD | 0 | yes | 6.6 |
| 904 | 66 | M | tAMD | 0 | no | 6.1 |
| 905 | 76 | M | tAMD | 1 | no | 2.1 |
| 906 | 84 | M | tAMD | 2 | no | 6.1 |
| 907 | 83 | M | tAMD | 1 | yes | 5.2 |
| 908 | 60 | F | tAMD | 0 | no | 1.2 |
| 909 | 78 | M | tAMD | 1 | no | 8.6 |
| 910 | 79 | M | tAMD | 0 | no | 1.9 |
| 911 | 83 | M | tAMD | 0 | no | 4.0 |
| 912 | 88 | M | tAMD | 1 | no | 2.1 |
| 913 | 77 | F | tAMD | 1 | yes | 6.8 |
| 914 | 82 | M | tAMD | 1 | no | 3.0 |
| 915 | 85 | M | tAMD | 1 | no | 2.9 |
| 916 | 67 | F | tAMD | 0 | no | 3.6 |
| 917 | 74 | M | tAMD | 1 | no | 6.8 |
| 918 | 84 | F | tAMD | 2 | no | 6.6 |
| 919 | 80 | M | tAMD | 0 | yes | 3.9 |
| 920 | 70 | M | tAMD | 1 | no | 10.0 |
| 921 | 77 | M | tAMD | 0 | no | 5.2 |
| 922 | 67 | M | tAMD | 0 | yes | 2.5 |
| 923 | 70 | F | tAMD | 2 | yes | 16.9 |
| 924 | 67 | F | tAMD | 0 | no | 3.1 |
| 925 | 78 | M | tAMD | 1 | no | 3.9 |
| 926 | 72 | F | tAMD | 1 | no | 3.0 |
| 927 | 52 | M | tAMD | 1 | no | 1.1 |
| 928 | 75 | M | tAMD | 1 | yes | 7.4 |
| 929 | 87 | M | tAMD | 0 | no | 2.0 |
| 930 | 89 | M | tAMD | 1 | yes | 13.3 |
| 931 | 77 | M | tAMD | 1 | no | 8.8 |
| 932 | 61 | M | tAMD | 0 | no | 3.6 |
| 933 | 60 | M | tAMD | 1 | no | 3.2 |
| 934 | 74 | F | tAMD | 0 | no | 4.5 |
| 935 | 76 | M | tAMD | 1 | no | 9.6 |
| 936 | 51 | M | tAMD | 0 | no | 1.3 |
| 937 | 76 | M | tAMD | 0 | no | 1.4 |
| 938 | 68 | M | tAMD | 0 | yes | 4.0 |
| 939 | 58 | M | tAMD | 1 | no | 9.1 |
| 940 | 75 | M | tAMD | 0 | yes | 10.2 |
| 941 | 74 | M | tAMD | 1 | no | 1.1 |
| 942 | 75 | M | tAMD | 0 | no | 3.0 |
| 943 | 78 | M | tAMD | 1 | no | 4.8 |
| 944 | 53 | M | tAMD | 0 | no | 0.3 |
| 945 | 84 | F | tAMD | 1 | no | 0.2 |
| 946 | 80 | F | tAMD | 1 | no | 18.5 |
| 947 | 56 | M | tAMD | 1 | no | 1.7 |
| 948 | 74 | M | tAMD | 1 | yes | 20.0 |
| 949 | 89 | M | tAMD | 1 | no | 16.7 |
| 950 | 70 | F | tAMD | 0 | no | 1.9 |
| 951 | 71 | M | tAMD | 1 | no | 5.0 |
| 952 | 69 | M | tAMD | 1 | no | 3.0 |
| 953 | 78 | M | tAMD | 0 | no | 1.5 |
| 954 | 67 | M | tAMD | 0 | no | 2.9 |
| 955 | 77 | F | tAMD | 0 | no | 4.9 |
| 956 | 77 | M | tAMD | 1 | no | 1.2 |
| 957 | 76 | F | tAMD | 1 | no | 9.5 |
| 958 | 84 | M | tAMD | 0 | no | 1.3 |
| 959 | 72 | M | tAMD | 0 | yes | 5.8 |
| 960 | 88 | M | tAMD | 1 | no | 7.7 |
| 961 | 80 | M | tAMD | 1 | no | 9.5 |
| 962 | 70 | M | tAMD | 0 | yes | 17.9 |
| 963 | 77 | M | tAMD | 1 | yes | 12.1 |
| 964 | 75 | F | tAMD | 0 | yes | 17.0 |
| 965 | 60 | F | tAMD | 1 | no | 2.7 |
| 966 | 79 | M | tAMD | 0 | yes | 8.3 |
| 967 | 78 | M | tAMD | 0 | yes | 4.8 |
| 968 | 83 | M | tAMD | 1 | no | 6.0 |
| 969 | 62 | M | tAMD | 0 | no | 1.7 |
| 970 | 77 | M | tAMD | 0 | no | 3.0 |
| 971 | 81 | M | tAMD | 0 | no | 8.2 |
| 972 | 79 | F | tAMD | 0 | no | 4.8 |
| 973 | 67 | M | tAMD | 1 | no | 1.0 |
| 974 | 80 | M | tAMD | 0 | yes | 8.2 |
| 975 | 80 | M | tAMD | 0 | no | 4.8 |
| 976 | 93 | M | tAMD | 1 | no | 8.4 |
| 977 | 87 | F | tAMD | 0 | yes | 8.8 |
| 978 | 81 | M | tAMD | 0 | no | 1.4 |
| 979 | 83 | M | tAMD | 1 | no | 3.8 |
| 980 | 85 | F | tAMD | 0 | no | 1.8 |
| 981 | 72 | M | tAMD | 0 | no | 0.6 |
| 982 | 84 | M | tAMD | 1 | no | 2.5 |
| 983 | 59 | M | tAMD | 2 | no | 14.7 |
| 984 | 84 | M | tAMD | 0 | no | 3.8 |
| 985 | 80 | F | tAMD | 2 | no | 32.1 |
| 986 | 76 | M | tAMD | 1 | no | 4.5 |
| 987 | 79 | M | tAMD | 1 | no | 4.1 |
| 988 | 62 | F | tAMD | 0 | no | 2.9 |
| 989 | 68 | M | tAMD | 1 | no | 8.0 |
| 990 | 69 | M | tAMD | 1 | no | 6.7 |
| 991 | 82 | M | tAMD | 1 | no | 5.7 |
| 992 | 75 | M | tAMD | 0 | no | 1.1 |
| 993 | 80 | M | tAMD | 0 | no | 1.4 |
| 994 | 80 | M | tAMD | 0 | yes | 3.9 |
| 995 | 80 | M | tAMD | 1 | no | 4.6 |
| 996 | 77 | M | tAMD | 1 | no | 5.6 |
| 997 | 72 | M | tAMD | 1 | yes | 20.2 |
| 998 | 68 | M | tAMD | 0 | no | 1.3 |
| 999 | 76 | M | tAMD | 1 | no | 3.8 |
| 1000 | 85 | M | tAMD | 1 | no | 21.8 |
| 1001 | 80 | M | tAMD | 1 | no | 7.5 |
| 1002 | 68 | M | tAMD | 0 | no | 3.3 |
| 1003 | 76 | M | tAMD | 0 | no | 3.5 |
| 1004 | 74 | M | tAMD | 0 | no | 11.7 |
| 1005 | 69 | M | tAMD | 1 | no | 9.7 |
| 1006 | 71 | F | tAMD | 1 | no | 2.2 |
| 1007 | 79 | Ｍ | tAMD | 1 | yes | 6.2 |
| 1008 | 75 | M | tAMD | 0 | no | 2.9 |
| 1009 | 79 | M | tAMD | 0 | no | 2.4 |
| 1010 | 77 | M | tAMD | 1 | no | 3.7 |
| 1011 | 68 | F | tAMD | 0 | no | 1.3 |
| 1012 | 87 | M | tAMD | 0 | no | 0.9 |
| 1013 | 72 | M | tAMD | 0 | yes | 2.9 |
| 1014 | 82 | M | tAMD | 1 | no | 4.6 |
| 1015 | 86 | M | tAMD | 0 | no | 4.0 |
| 1016 | 86 | M | tAMD | 0 | no | 4.6 |
| 1017 | 68 | M | tAMD | 1 | no | 13.4 |
| 1018 | 72 | M | tAMD | 1 | no | 8.0 |
| 1019 | 85 | F | tAMD | 0 | no | 0.2 |
| 1020 | 84 | F | tAMD | 0 | no | 2.6 |
| 1021 | 80 | M | tAMD | 0 | yes | 3.8 |
| 1022 | 81 | M | tAMD | 0 | no | 2.0 |
| 1023 | 80 | M | tAMD | 1 | yes | 12.1 |
| 1024 | 78 | M | tAMD | 1 | no | 3.0 |
| 1025 | 80 | M | tAMD | 1 | yes | 14.8 |
|  |  |  | tAMD | 0 | no | 10.0 |
| 1026 | 71 | M | tAMD | 0 | no | 3.8 |
| 1027 | 69 | M | tAMD | 0 | no | 1.3 |
| 1028 | 72 | M | tAMD | 0 | yes | 2.8 |
| 1029 | 79 | M | tAMD | 0 | no | 3.7 |
| 1030 | 75 | M | tAMD | 0 | no | 2.0 |
| 1031 | 89 | M | tAMD | 0 | no | 2.4 |
| 1032 | 65 | M | tAMD | 0 | no | 5.9 |
| 1033 | 81 | M | tAMD | 1 | no | 9.6 |
| 1034 | 69 | F | tAMD | 0 | no | 9.7 |
| 1035 | 66 | F | tAMD | 0 | no | 3.5 |
| 1036 | 82 | M | tAMD | 1 | no | 5.0 |
| 1037 | 79 | M | tAMD | 0 | no | 1.1 |
| 1038 | 78 | F | tAMD | 1 | no | 3.9 |
| 1039 | 63 | M | PCV | 0 | yes | 18.0 |
| 1040 | 81 | M | PCV | 0 | yes | 7.5 |
| 1041 | 66 | M | PCV | 0 | no | 1.4 |
| 1042 | 77 | M | PCV | 1 | no | 2.1 |
| 1043 | 80 | M | PCV | 1 | no | 5.8 |
| 1044 | 65 | F | PCV | 1 | yes | 18.7 |
| 1045 | 83 | Ｍ | PCV | 1 | no | 5.0 |
| 1046 | 69 | M | PCV | 0 | yes | 5.6 |
| 1047 | 81 | F | PCV | 0 | yes | 4.1 |
| 1048 | 62 | M | PCV | 0 | no | 1.5 |
| 1049 | 77 | M | PCV | 0 | no | 2.4 |
| 1050 | 57 | F | PCV | 0 | no | 2.3 |
| 1051 | 82 | M | PCV | 0 | no | 5.6 |
| 1052 | 75 | M | PCV | 1 | yes | 14.2 |
| 1053 | 69 | F | PCV | 0 | no | 6.1 |
| 1054 | 85 | M | PCV | 0 | no | 4.6 |
| 1055 | 83 | M | PCV | 0 | yes | 3.9 |
| 1056 | 69 | M | PCV | 0 | no | 1.2 |
| 1057 | 64 | M | PCV | 0 | yes | 2.4 |
| 1058 | 79 | M | PCV | 0 | no | 1.8 |
| 1059 | 88 | M | PCV | 0 | yes | 6.0 |
| 1060 | 84 | M | PCV | 1 | yes | 11.4 |
| 1061 | 83 | F | PCV | 2 | yes | 12.4 |
| 1062 | 68 | M | PCV | 1 | no | 6.4 |
| 1063 | 73 | M | PCV | 0 | no | 2.8 |
| 1064 | 77 | M | PCV | 0 | no | 1.7 |
| 1065 | 84 | F | PCV | 0 | no | 3.7 |
| 1066 | 75 | M | PCV | 1 | yes | 19.4 |
| 1067 | 67 | M | PCV | 0 | no | 5.8 |
| 1068 | 67 | F | PCV | 0 | no | 2.8 |
| 1069 | 79 | F | PCV | 0 | no | 3.1 |
| 1070 | 83 | M | PCV | 0 | no | 1.0 |
| 1071 | 79 | F | PCV | 0 | no | 6.5 |
| 1072 | 55 | M | PCV | 0 | no | 1.7 |
| 1073 | 77 | F | PCV | 1 | yes | 2.2 |
| 1074 | 73 | M | PCV | 1 | no | 2.9 |
| 1075 | 77 | M | PCV | 0 | no | 5.6 |
| 1076 | 67 | M | PCV | 0 | no | 6.0 |
| 1077 | 65 | M | PCV | 0 | no | 1.2 |
| 1078 | 54 | M | PCV | 0 | no | 6.1 |
| 1079 | 79 | M | PCV | 1 | yes | 12.0 |
| 1080 | 67 | M | PCV | 0 | yes | 1.9 |
| 1081 | 76 | M | PCV | 0 | no | 1.6 |
| 1082 | 60 | M | PCV | 0 | yes | 3.6 |
| 1083 | 74 | F | PCV | 1 | no | 4.8 |
| 1084 | 74 | M | PCV | 1 | yes | 7.0 |
| 1085 | 78 | M | PCV | 0 | no | 0.9 |
| 1086 | 69 | M | PCV | 1 | yes | 11.8 |
| 1087 | 74 | Ｆ | PCV | 0 | no | 11.7 |
| 1088 | 71 | F | PCV | 1 | no | 1.9 |
| 1089 | 79 | M | PCV | 1 | yes | 18.7 |
| 1090 | 59 | F | PCV | 1 | yes | 3.8 |
| 1091 | 63 | F | PCV | 0 | no | 0.7 |
| 1092 | 68 | F | PCV | 0 | yes | 23.6 |
| 1093 | 78 | M | PCV | 0 | no | 1.7 |
| 1094 | 76 | M | PCV | 1 | no | 3.7 |
| 1095 | 71 | M | PCV | 0 | no | 2.6 |
| 1096 | 81 | M | PCV | 1 | yes | 4.6 |
| 1097 | 64 | M | PCV | 0 | yes | 5.0 |
| 1098 | 80 | M | PCV | 1 | no | 3.2 |
| 1099 | 66 | M | PCV | 0 | no | 6.6 |
| 1100 | 80 | M | PCV | 0 | no | 2.6 |
| 1101 | 69 | F | PCV | 0 | no | 1.9 |
| 1102 | 81 | M | PCV | 0 | yes | 2.6 |
| 1103 | 79 | M | PCV | 1 | no | 7.5 |
| 1104 | 73 | M | PCV | 0 | no | 8.0 |
| 1105 | 80 | F | PCV | 0 | no | 1.9 |
| 1106 | 79 | M | PCV | 0 | no | 3.4 |
| 1107 | 76 | M | PCV | 1 | yes | 3.0 |
| 1108 | 77 | M | PCV | 0 | no | 2.6 |
| 1109 | 67 | M | PCV | 1 | yes | 23.3 |
| 1110 | 70 | M | PCV | 1 | no | 5.2 |
| 1111 | 70 | M | PCV | 1 | no | 15.0 |
| 1112 | 67 | M | PCV | 1 | no | 4.5 |
| 1113 | 60 | F | PCV | 0 | yes | 6.7 |
| 1114 | 52 | F | PCV | 1 | no | 14.0 |
| 1115 | 76 | M | PCV | 1 | no | 5.1 |
| 1116 | 57 | Ｍ | PCV | 0 | no | 10.4 |
| 1117 | 67 | M | PCV | 0 | no | 2.7 |
| 1118 | 70 | F | PCV | 0 | no | 3.1 |
| 1119 | 79 | M | PCV | 0 | no | 3.7 |
| 1120 | 77 | M | PCV | 0 | no | 2.2 |
| 1121 | 86 | F | PCV | 0 | yes | 15.0 |
| 1122 | 80 | F | PCV | 1 | no | 14.9 |
| 1123 | 83 | M | PCV | 1 | no | 1.2 |
| 1124 | 75 | M | PCV | 0 | no | 4.4 |
| 1125 | 57 | M | PCV | 0 | no | 12.0 |
| 1126 | 70 | M | PCV | 1 | no | 5.2 |
| 1127 | 59 | Ｍ | PCV | 1 | no | 5.0 |
| 1128 | 82 | M | PCV | 1 | yes | 0.3 |
| 1129 | 75 | M | PCV | 1 | no | 0.5 |
| 1130 | 76 | M | PCV | 1 | no | 4.0 |
| 1131 | 71 | M | PCV | 0 | no | 2.6 |
| 1132 | 81 | M | PCV | 0 | yes | 26.3 |
| 1133 | 77 | M | PCV | 0 | no | 2.9 |
| 1134 | 70 | M | PCV | 0 | no | 2.3 |
| 1135 | 77 | M | PCV | 0 | no | 1.0 |
| 1136 | 86 | F | PCV | 0 | no | 9.7 |
| 1137 | 58 | F | PCV | 0 | no | 1.1 |
| 1138 | 72 | M | PCV | 1 | no | 4.6 |
| 1139 | 68 | F | PCV | 1 | no | 1.0 |
| 1140 | 81 | M | PCV | 0 | no | 8.3 |
| 1141 | 76 | M | PCV | 0 | no | 3.2 |
| 1142 | 61 | F | PCV | 1 | no | 0.4 |
| 1143 | 70 | M | PCV | 0 | no | 8.8 |
| 1144 | 82 | F | PCV | 0 | yes | 9.7 |
| 1145 | 74 | M | PCV | 0 | no | 1.7 |
| 1146 | 76 | M | PCV | 1 | yes | 6.3 |
| 1147 | 81 | M | PCV | 0 | no | 2.3 |
| 1148 | 77 | F | PCV | 1 | no | 0.7 |
| 1149 | 80 | M | PCV | 0 | no | 0.7 |
| 1150 | 73 | M | PCV | 0 | yes | 20.0 |
| 1151 | 73 | M | PCV | 1 | yes | 4.3 |
| 1152 | 91 | M | PCV | 1 | no | 2.5 |
| 1153 | 68 | M | PCV | 0 | yes | 0.5 |
| 1154 | 73 | M | PCV | 0 | yes | 10.9 |
| 1155 | 85 | M | PCV | 0 | no | 3.6 |
| 1156 | 78 | M | PCV | 0 | yes | 7.0 |
| 1157 | 87 | M | PCV | 1 | no | 7.8 |
| 1158 | 66 | M | PCV | 0 | no | 3.8 |
| 1159 | 82 | M | PCV | 0 | no | 2.2 |
| 1160 | 84 | F | PCV | 1 | no | 3.0 |
| 1161 | 86 | F | PCV | 1 | no | 8.2 |
| 1162 | 79 | F | PCV | 0 | no | 4.8 |
| 1163 | 87 | F | PCV | 1 | yes | 8.3 |
| 1164 | 61 | M | PCV | 0 | yes | 15.6 |
| 1165 | 76 | F | PCV | 2 | yes | 11.8 |
| 1166 | 79 | M | PCV | 0 | no | 3.3 |
| 1167 | 78 | M | PCV | 0 | yes | 36.3 |
| 1168 | 72 | M | PCV | 0 | no | 1.0 |
| 1169 | 73 | M | PCV | 0 | no | 1.3 |
| 1170 | 77 | M | PCV | 0 | yes | 16.8 |
| 1171 | 73 | M | PCV | 1 | no | 4.1 |
| 1172 | 77 | M | PCV | 0 | no | 2.1 |
| 1173 | 75 | F | PCV | 0 | yes | 11.2 |
| 1174 | 77 | M | PCV | 0 | no | 4.1 |
| 1175 | 68 | F | RAP | 0 | no | 1.1 |
| 1176 | 73 | M | RAP | 0 | no | 1.4 |
| 1177 | 81 | F | RAP | 1 | yes | 9.0 |
|  |  |  | RAP | 0 | no | 2.3 |
| 1178 | 92 | F | RAP | 1 | yes | 2.2 |
| 1179 | 82 | M | RAP | 1 | no | 1.3 |
|  |  |  | RAP | 1 | no | 1.7 |
| 1180 | 79 | M | RAP | 1 | no | 2.1 |
|  |  |  | RAP | 0 | no | 0.3 |
| 1181 | 87 | F | RAP | 0 | no | 1.5 |
| 1182 | 79 | M | RAP | 1 | yes | 6.2 |
| 1183 | 83 | F | RAP | 1 | yes | 7.2 |
|  |  |  | RAP | 1 | yes | 14.5 |
| 1184 | 79 | F | RAP | 1 | yes | 2.0 |
| 1185 | 80 | M | RAP | 0 | no | 2.4 |
| 1186 | 94 | M | RAP | 0 | yes | 5.9 |
| 1187 | 84 | M | RAP | 0 | yes | 6.9 |
| 1188 | 91 | F | RAP | 1 | yes | 11.1 |
| 1189 | 84 | M | RAP | 0 | yes | 1.8 |
| 1190 | 83 | M | RAP | 1 | no | 1.2 |
| 1191 | 81 | M | RAP | 0 | no | 1.4 |
| 1192 | 84 | F | RAP | 0 | yes | 5.4 |
|  |  |  | RAP | 2 | yes | 4.9 |
| 1193 | 75 | M | RAP | 0 | yes | 7.6 |
| 1194 | 89 | F | RAP | 0 | no | 1.1 |
| 1195 | 66 | F | RAP | 1 | yes | 14.1 |
| 1196 | 86 | F | RAP | 1 | yes | 1.3 |
| 1197 | 83 | M | RAP | 0 | yes | 4.6 |
| 1198 | 72 | M | RAP | 1 | no | 4.1 |
| 1199 | 86 | M | RAP | 1 | yes | 25.0 |
| 1200 | 76 | M | RAP | 0 | yes | 4.9 |
| 1201 | 88 | M | RAP | 1 | yes | 14.3 |
| 1202 | 87 | F | RAP | 1 | yes | 3.7 |
| 1203 | 81 | M | RAP | 0 | no | 1.4 |
| 1204 | 75 | F | RAP | 0 | no | 2.6 |
| 1205 | 79 | M | RAP | 1 | yes | 13.2 |
|  |  |  | RAP | 0 | no | 1.0 |
| 1206 | 82 | F | RAP | 1 | no | 0.8 |
| 1207 | 85 | F | RAP | 1 | no | 2.5 |
| 1208 | 82 | M | RAP | 1 | yes | 2.4 |
| 1209 | 74 | F | RAP | 0 | yes | 0.9 |
| 1210 | 89 | F | RAP | 1 | yes | 4.0 |
| 1211 | 88 | F | RAP | 1 | no | 0.9 |
| 1212 | 83 | M | combined | 1 | no | NA |
|  |  |  | combined | 1 | no | NA |
| 1213 | 82 | F | combined | 0 | no | NA |
|  |  |  | combined | 0 | no | NA |
| Phase V |  |  |  |  |  |  |
| patient No. | age | sex | AMD Subtype | LogMAR BCVA | PED | Lesion size (DA) |
| 1214 | 57 | M | tAMD | 0 | no | 0.5 |
| 1215 | 82 | M | tAMD | 2 | no | 4.3 |
| 1216 | 84 | M | tAMD | 0 | no | 20.5 |
| 1217 | 84 | F | tAMD | 0 | no | 11.5 |
| 1218 | 74 | M | tAMD | 0 | no | 1.3 |
| 1219 | 75 | M | tAMD | 1 | no | 4.6 |
| 1220 | 88 | F | tAMD | 0 | no | 6.3 |
| 1221 | 83 | M | tAMD | 0 | no | 2.6 |
| 1222 | 83 | F | tAMD | 1 | yes | 12.4 |
| 1223 | 84 | M | tAMD | 1 | no | 9.0 |
| 1224 | 51 | F | tAMD | 0 | no | 3.4 |
| 1225 | 83 | M | tAMD | 1 | no | 6.4 |
| 1226 | 75 | M | tAMD | 0 | no | 2.5 |
| 1227 | 91 | M | tAMD | 1 | no | 7.7 |
| 1228 | 67 | M | tAMD | 0 | no | 4.9 |
| 1229 | 70 | M | tAMD | 0 | no | 0.6 |
| 1230 | 69 | M | tAMD | 0 | no | 2.6 |
| 1231 | 78 | F | tAMD | 0 | yes | 8.5 |
| 1232 | 75 | F | tAMD | 1 | yes | 18.1 |
| 1233 | 55 | F | tAMD | 0 | no | 0.6 |
| 1234 | 80 | M | tAMD | 0 | no | 4.6 |
| 1235 | 64 | M | tAMD | 0 | no | 16.5 |
| 1236 | 88 | F | tAMD | 1 | no | 5.0 |
| 1237 | 70 | F | tAMD | 0 | no | 3.5 |
| 1238 | 78 | M | tAMD | 1 | no | 2.3 |
| 1239 | 69 | M | tAMD | 0 | no | 0.7 |
| 1240 | 81 | M | tAMD | 1 | no | 2.4 |
| 1241 | 80 | F | tAMD | 1 | no | 4.5 |
| 1242 | 49 | F | tAMD | 1 | no | 0.6 |
| 1243 | 77 | M | tAMD | 0 | yes | 2.7 |
| 1244 | 71 | M | tAMD | 0 | no | 12.2 |
| 1245 | 75 | M | tAMD | 1 | no | 8.8 |
| 1246 | 79 | M | tAMD | 0 | yes | 1.5 |
| 1247 | 77 | F | tAMD | 0 | no | 4.3 |
| 1248 | 77 | M | tAMD | 0 | no | 9.1 |
| 1249 | 81 | M | tAMD | 1 | no | 1.1 |
| 1250 | 81 | M | tAMD | 0 | no | 1.8 |
| 1251 | 68 | M | tAMD | 1 | no | 3.0 |
| 1252 | 88 | M | tAMD | 0 | no | 2.5 |
| 1253 | 78 | M | tAMD | 0 | no | 9.9 |
|  |  |  | tAMD | 1 | no | 16.4 |
| 1254 | 65 | M | tAMD | 1 | no | 3.5 |
| 1255 | 73 | M | tAMD | 0 | no | 3.0 |
| 1256 | 74 | M | tAMD | 1 | no | 2.7 |
| 1257 | 64 | M | tAMD | 1 | yes | 24.4 |
| 1258 | 73 | M | tAMD | 0 | no | 1.4 |
| 1259 | 87 | F | tAMD | 0 | no | 8.2 |
| 1260 | 82 | M | tAMD | 1 | no | 17.0 |
| 1261 | 76 | M | tAMD | 1 | no | 2.8 |
| 1262 | 82 | M | tAMD | 0 | no | 10.4 |
| 1263 | 72 | M | tAMD | 1 | no | 2.9 |
| 1264 | 80 | M | tAMD | 2 | no | 4.4 |
| 1265 | 83 | M | tAMD | 1 | no | 2.4 |
| 1266 | 79 | M | tAMD | 0 | no | 5.0 |
| 1267 | 74 | M | tAMD | 1 | no | 8.7 |
| 1268 | 73 | M | tAMD | 0 | yes | 5.1 |
| 1269 | 53 | F | tAMD | 0 | no | 1.8 |
| 1270 | 79 | M | tAMD | 0 | no | 2.3 |
| 1271 | 78 | M | tAMD | 1 | no | 3.4 |
| 1272 | 69 | M | tAMD | 0 | no | 5.5 |
| 1273 | 84 | F | tAMD | 1 | yes | 10.1 |
| 1274 | 93 | M | tAMD | 1 | no | 6.3 |
| 1275 | 74 | M | tAMD | 0 | no | 1.8 |
| 1276 | 78 | M | tAMD | 0 | no | 18.0 |
| 1277 | 80 | M | tAMD | 0 | no | 5.8 |
| 1278 | 72 | M | tAMD | 1 | no | 3.6 |
| 1279 | 58 | M | tAMD | 0 | no | 3.9 |
| 1280 | 80 | M | tAMD | 0 | no | 0.9 |
| 1281 | 79 | M | tAMD | 0 | no | 1.3 |
| 1282 | 75 | M | tAMD | 0 | no | 2.1 |
| 1283 | 79 | M | tAMD | 1 | no | 1.9 |
| 1284 | 85 | F | tAMD | 2 | no | 18.5 |
| 1285 | 84 | M | tAMD | 1 | no | 9.5 |
| 1286 | 77 | M | tAMD | 1 | no | 1.4 |
| 1287 | 77 | M | tAMD | 1 | no | 10.3 |
| 1288 | 78 | M | tAMD | 1 | no | 14.1 |
| 1289 | 74 | M | tAMD | 1 | no | 7.1 |
| 1290 | 80 | M | tAMD | 0 | no | 0.8 |
| 1291 | 89 | M | tAMD | 1 | yes | 9.6 |
|  |  |  | tAMD | 1 | no | 2.3 |
| 1292 | 77 | M | tAMD | 0 | yes | 10.5 |
| 1293 | 81 | M | tAMD | 1 | no | 1.0 |
| 1294 | 76 | M | tAMD | 1 | no | 1.8 |
| 1295 | 90 | F | tAMD | 1 | no | 3.9 |
| 1296 | 70 | F | tAMD | 1 | yes | 15.1 |
| 1297 | 75 | F | tAMD | 0 | yes | 25.3 |
| 1298 | 83 | M | tAMD | 0 | no | 3.9 |
| 1299 | 77 | F | tAMD | 0 | no | 2.0 |
| 1300 | 56 | M | tAMD | 0 | no | 2.9 |
| 1301 | 83 | M | tAMD | 0 | no | 0.3 |
| 1302 | 52 | M | tAMD | 0 | no | 1.7 |
| 1303 | 63 | M | tAMD | 0 | yes | 0.5 |
| 1304 | 71 | M | tAMD | 0 | no | 3.3 |
| 1305 | 73 | F | tAMD | 0 | no | 3.9 |
| 1306 | 75 | F | tAMD | 0 | no | 0.9 |
| 1307 | 69 | F | tAMD | 0 | no | 6.7 |
| 1308 | 81 | M | tAMD | 1 | no | 1.9 |
| 1309 | 77 | M | tAMD | 1 | no | 1.9 |
| 1310 | 87 | M | tAMD | 1 | no | 6.3 |
| 1311 | 80 | F | tAMD | 2 | yes | 39.4 |
| 1312 | 74 | M | tAMD | 0 | no | 4.3 |
| 1313 | 66 | M | tAMD | 1 | no | 12.0 |
| 1314 |  | M | tAMD | 1 | no | 0.3 |
| 1315 | 86 | M | tAMD | 1 | yes | 12.9 |
| 1316 | 85 | M | tAMD | 0 | no | 1.8 |
| 1317 | 85 | M | tAMD | 1 | no | 7.5 |
| 1318 | 78 | M | tAMD | 1 | yes | 6.5 |
| 1319 | 63 | M | tAMD | 1 | no | 3.7 |
| 1320 | 82 | F | tAMD | 0 | no | 3.9 |
| 1321 | 67 | M | tAMD | 0 | no | 1.3 |
| 1322 | 76 | M | tAMD | 0 | no | 0.9 |
| 1323 | 80 | M | tAMD | 0 | no | 2.3 |
| 1324 | 89 | M | tAMD | 2 | yes | 29.8 |
| 1325 | 86 | M | tAMD | 1 | no | 6.1 |
| 1326 | 75 | M | tAMD | 0 | yes | 9.7 |
| 1327 | 78 | M | tAMD | 0 | no | 7.8 |
| 1328 | 64 | M | tAMD | 0 | no | 11.2 |
| 1329 | 78 | M | tAMD | 1 | no | 3.5 |
| 1330 | 72 | M | tAMD | 0 | no | 27.3 |
| 1331 | 80 | M | tAMD | 0 | no | 5.6 |
| 1332 | 86 | M | tAMD | 1 | no | 6.4 |
| 1333 | 77 | F | tAMD | 0 | no | 1.4 |
| 1334 | 74 | M | tAMD | 0 | no | 0.4 |
| 1335 | 80 | M | tAMD | 0 | no | 4.0 |
| 1336 | 70 | M | tAMD | 0 | no | 1.4 |
| 1337 | 60 | F | tAMD | 0 | no | 1.0 |
| 1338 | 80 | M | tAMD | 0 | no | 3.1 |
| 1339 | 84 | F | tAMD | 1 | no | 0.4 |
|  |  |  | tAMD | 2 | no | 4.6 |
| 1340 | 84 | F | tAMD | 0 | no | 9.7 |
| 1341 | 73 | M | tAMD | 1 | no | 3.4 |
| 1342 | 83 | M | tAMD | 1 | no | 1.7 |
| 1343 | 87 | F | tAMD | 1 | no | 2.3 |
| 1344 | 77 | M | tAMD | 0 | no | 1.5 |
| 1345 | 80 | M | tAMD | 0 | no | 1.0 |
| 1346 | 77 | F | tAMD | 0 | yes | 25.1 |
| 1347 | 81 | M | tAMD | 0 | yes | 2.3 |
| 1348 | 87 | F | tAMD | 1 | yes | 33.1 |
| 1349 | 79 | M | tAMD | 0 | no | 5.1 |
| 1350 | 71 | M | tAMD | 1 | no | 12.3 |
| 1351 | 80 | F | tAMD | 1 | no | 11.0 |
| 1352 | 71 | M | tAMD | 0 | no | 1.7 |
| 1353 | 75 | M | tAMD | 0 | no | 2.5 |
| 1354 | 69 | M | tAMD | 0 | yes | 3.3 |
| 1355 | 66 | M | tAMD | 0 | no | 0.5 |
| 1356 | 63 | F | tAMD | 0 | no | 1.4 |
| 1357 | 83 | F | tAMD | 2 | no | 2.7 |
| 1358 | 77 | M | tAMD | 0 | yes | 7.5 |
| 1359 | 78 | M | tAMD | 0 | no | 0.2 |
| 1360 | 75 | F | tAMD | 0 | no | 4.0 |
| 1361 | 66 | M | tAMD | 0 | no | 1.6 |
| 1362 | 79 | M | tAMD | 1 | no | 2.1 |
| 1363 | 79 | F | tAMD | 0 | no | 0.2 |
| 1364 | 79 | F | tAMD | 0 | no | 2.4 |
| 1365 | 80 | F | tAMD | 1 | no | 14.3 |
| 1366 | 65 | M | tAMD | 1 | no | 1.7 |
| 1367 | 71 | M | tAMD | 1 | no | 3.7 |
| 1368 | 69 | M | tAMD | 0 | no | 5.4 |
| 1369 | 76 | M | tAMD | 1 | no | 2.1 |
| 1370 | 69 | F | tAMD | 1 | no | 1.4 |
| 1371 | 77 | M | tAMD | 0 | no | 0.7 |
|  |  |  | tAMD | 0 | no | 0.4 |
| 1372 | 81 | M | tAMD | 0 | no | 3.1 |
| 1373 | 63 | M | tAMD | 0 | no | 0.8 |
| 1374 | 71 | F | tAMD | 0 | no | 2.6 |
| 1375 | 84 | F | tAMD | 0 | no | 3.2 |
| 1376 | 63 | F | tAMD | 1 | no | 1.1 |
| 1377 | 61 | F | tAMD | 0 | no | 0.4 |
| 1378 | 87 | M | tAMD | 1 | no | 1.5 |
| 1379 | 81 | M | tAMD | 1 | no | 3.4 |
| 1380 | 77 | M | tAMD | 0 | no | 0.4 |
| 1381 | 70 | M | tAMD | 1 | no | 0.3 |
| 1382 | 79 | M | tAMD | 0 | no | 4.5 |
| 1383 | 63 | M | tAMD | 1 | no | 2.3 |
| 1384 | 90 | F | tAMD | 1 | no | 0.5 |
| 1385 | 82 | M | tAMD | 1 | no | 2.9 |
| 1386 | 87 | M | tAMD | 1 | no | 3.0 |
| 1387 | 83 | F | tAMD | 1 | no | 7.0 |
| 1388 | 86 | M | tAMD | 1 | no | 2.2 |
| 1389 | 82 | F | tAMD | 1 | yes | 12.0 |
| 1390 | 65 | M | tAMD | 0 | no | 5.0 |
| 1391 | 80 | M | tAMD | 1 | no | 1.8 |
| 1392 | 66 | M | PCV | 0 | no | 5.0 |
| 1393 | 67 | M | PCV | 0 | no | 1.9 |
| 1394 | 88 | F | PCV | 0 | no | 4.0 |
| 1395 | 75 | M | PCV | 1 | no | 3.5 |
| 1396 | 56 | M | PCV | 0 | no | 0.8 |
| 1397 | 84 | M | PCV | 0 | no | 2.6 |
| 1398 | 69 | M | PCV | 1 | no | 5.7 |
| 1399 | 83 | M | PCV | 1 | no | 0.8 |
| 1400 | 58 | M | PCV | 0 | no | 1.2 |
| 1401 | 79 | M | PCV | 0 | no | 3.5 |
| 1402 | 73 | F | PCV | 0 | no | 8.3 |
| 1403 | 84 | F | PCV | 1 | no | 1.4 |
| 1404 | 71 | M | PCV | 0 | no | 0.2 |
| 1405 | 78 | M | PCV | 1 | yes | 10.2 |
| 1406 | 84 | M | PCV | 0 | no | 2.4 |
| 1407 | 71 | M | PCV | 1 | no | 8.8 |
| 1408 | 69 | M | PCV | 0 | no | 0.5 |
| 1409 | 84 | M | PCV | 0 | no | 8.9 |
| 1410 | 60 | M | PCV | 0 | yes | 7.7 |
| 1411 | 70 | M | PCV | 0 | yes | 12.4 |
| 1412 | 76 | M | PCV | 1 | no | 2.0 |
| 1413 | 60 | M | PCV | 0 | yes | 1.5 |
| 1414 | 78 | M | PCV | 1 | no | 22.4 |
| 1415 | 61 | M | PCV | 0 | no | 4.6 |
| 1416 | 72 | M | PCV | 2 | no | 3.1 |
| 1417 | 78 | M | PCV | 1 | no | 1.2 |
| 1418 | 67 | M | PCV | 0 | no | 10.1 |
| 1419 | 83 | M | PCV | 1 | yes | 8.5 |
| 1420 | 71 | F | PCV | 0 | yes | 32.3 |
| 1421 | 75 | M | PCV | 0 | no | 2.8 |
| 1422 | 64 | M | PCV | 1 | no | 2.4 |
| 1423 | 77 | F | PCV | 1 | no | 2.0 |
| 1424 | 61 | M | PCV | 1 | no | 7.3 |
| 1425 | 72 | M | PCV | 0 | no | 0.4 |
| 1426 | 61 | M | PCV | 0 | no | 1.0 |
| 1427 | 79 | M | PCV | 0 | no | 2.0 |
| 1428 | 71 | M | PCV | 0 | yes | 15.2 |
| 1429 | 72 | M | PCV | 0 | no | 0.5 |
| 1430 | 66 | M | PCV | 1 | no | 4.3 |
| 1431 | 75 | M | PCV | 0 | no | 2.5 |
| 1432 | 56 | M | PCV | 0 | no | 1.6 |
| 1433 | 79 | M | PCV | 1 | yes | 4.0 |
| 1434 | 74 | M | PCV | 0 | yes | 5.5 |
| 1435 | 90 | M | PCV | 0 | no | 18.3 |
| 1436 | 83 | M | PCV | 1 | no | 9.5 |
| 1437 | 71 | M | PCV | 0 | no | 2.2 |
| 1438 | 90 | M | PCV | 1 | no | 5.5 |
| 1439 | 63 | M | PCV | 0 | no | 1.4 |
| 1440 | 75 | F | PCV | 0 | yes | 3.0 |
| 1441 | 79 | M | PCV | 1 | yes | 13.0 |
| 1442 | 81 | F | PCV | 1 | no | 2.9 |
|  |  |  | PCV | 0 | no | 11.6 |
| 1443 | 70 | M | PCV | 0 | no | 2.9 |
| 1444 | 59 | M | PCV | 0 | yes | 23.0 |
| 1445 | 73 | M | PCV | 0 | yes | 8.1 |
| 1446 | 76 | F | PCV | 0 | no | 1.3 |
| 1447 | 76 | F | PCV | 1 | no | 1.2 |
| 1448 | 74 | F | PCV | 0 | no | 2.9 |
| 1449 | 79 | M | PCV | 1 | no | 3.7 |
| 1450 | 82 | M | PCV | 1 | no | 25.8 |
|  |  |  | PCV | 0 | no | 3.8 |
| 1451 | 74 | F | PCV | 0 | no | 8.1 |
| 1452 | 69 | M | PCV | 0 | no | 2.2 |
| 1453 | 77 | M | PCV | 0 | yes | 2.9 |
|  |  |  | PCV | 0 | yes | 4.7 |
| 1454 | 76 | M | PCV | 1 | no | 4.9 |
| 1455 | 76 | M | PCV | 1 | no | 2.1 |
| 1456 | 67 | F | PCV | 0 | yes | 17.8 |
| 1457 | 74 | F | PCV | 1 | no | 24.0 |
| 1458 | 72 | M | PCV | 0 | no | 0.9 |
| 1459 | 66 | M | PCV | 0 | yes | 3.2 |
| 1460 | 83 | M | PCV | 0 | yes | 2.2 |
| 1461 | 63 | M | PCV | 0 | yes | 18.3 |
| 1462 | 74 | F | PCV | 1 | no | 1.6 |
| 1463 | 80 | M | PCV | 1 | no | 2.0 |
| 1464 | 82 | F | PCV | 0 | no | 0.8 |
| 1465 | 88 | M | PCV | 1 | no | 1.9 |
| 1466 | 63 | M | PCV | 0 | yes | 7.4 |
| 1467 | 68 | F | PCV | 1 | no | 7.3 |
| 1468 | 71 | M | PCV | 1 | yes | 9.7 |
| 1469 | 78 | M | PCV | 0 | no | 0.8 |
| 1470 | 79 | M | PCV | 0 | no | 0.9 |
| 1471 | 61 | M | PCV | 0 | no | 3.0 |
| 1472 | 75 | M | PCV | 0 | no | 2.4 |
| 1473 | 72 | M | PCV | 0 | yes | 1.1 |
| 1474 | 77 | M | PCV | 0 | no | 2.1 |
| 1475 | 68 | F | PCV | 1 | no | 8.6 |
| 1476 | 76 | M | PCV | 0 | no | 5.9 |
| 1477 | 81 | M | PCV | 1 | no | 6.8 |
| 1478 | 54 | F | PCV | 0 | no | 4.6 |
| 1479 | 76 | M | PCV | 1 | no | 4.7 |
| 1480 | 69 | M | PCV | 1 | no | 12.7 |
| 1481 | 84 | M | PCV | 1 | no | 33.4 |
| 1482 | 75 | M | PCV | 0 | no | 2.9 |
| 1483 | 67 | M | PCV | 1 | no | 5.9 |
| 1484 | 67 | M | PCV | 0 | no | 0.6 |
| 1485 | 80 | M | PCV | 1 | no | 5.7 |
| 1486 | 74 | M | PCV | 0 | no | 2.3 |
| 1487 | 72 | M | PCV | 1 | yes | 7.6 |
|  |  |  | PCV | 0 | no | 6.1 |
| 1488 | 80 | M | PCV | 1 | no | 2.1 |
| 1489 | 62 | M | PCV | 2 | yes | 39.5 |
| 1490 | 63 | M | PCV | 0 | no | 2.8 |
| 1491 | 76 | M | PCV | 1 | no | 13.8 |
| 1492 | 80 | M | PCV | 1 | yes | 3.0 |
| 1493 | 71 | F | PCV | 1 | no | 2.5 |
| 1494 | 71 | M | PCV | 1 | yes | 3.4 |
| 1495 | 79 | M | PCV | 0 | no | 3.5 |
| 1496 | 79 | M | PCV | 1 | no | 1.0 |
| 1497 | 68 | M | PCV | 0 | no | 0.6 |
| 1498 | 65 | M | PCV | 0 | no | 1.8 |
| 1499 | 78 | M | PCV | 1 | yes | 7.6 |
| 1500 | 68 | F | PCV | 0 | no | 2.3 |
| 1501 | 73 | M | PCV | 0 | yes | 2.2 |
| 1502 | 74 | M | PCV | 0 | no | 10.7 |
| 1503 | 72 | M | PCV | 1 | no | 4.2 |
| 1504 | 87 | F | PCV | 1 | yes | 1.6 |
| 1505 | 70 | F | PCV | 1 | no | 1.5 |
| 1506 | 71 | M | PCV | 0 | no | 1.1 |
| 1507 | 70 | M | PCV | 1 | no | 4.6 |
| 1508 | 70 | F | PCV | 0 | yes | 12.5 |
| 1509 | 75 | M | PCV | 0 | no | 5.8 |
| 1510 | 68 | M | PCV | 1 | no | 6.1 |
| 1511 | 61 | M | PCV | 0 | no | 4.5 |
| 1512 | 81 | M | PCV | 0 | no | 2.0 |
| 1513 | 71 | M | PCV | 1 | no | 1.2 |
| 1514 | 75 | M | PCV | 0 | no | 1.8 |
| 1515 | 73 | M | PCV | 0 | no | 4.9 |
| 1516 | 61 | M | PCV | 1 | no | 11.6 |
| 1517 | 82 | M | PCV | 0 | no | 4.2 |
| 1518 | 74 | M | PCV | 0 | yes | 3.5 |
|  |  |  | PCV | 0 | no | 1.6 |
| 1519 | 86 | M | PCV | 0 | no | 4.4 |
| 1520 | 64 | M | PCV | 0 | no | 1.2 |
| 1521 | 68 | M | PCV | 0 | no | 1.6 |
| 1522 | 76 | M | PCV | 1 | no | 4.6 |
| 1523 | 79 | M | PCV | 0 | no | 1.8 |
| 1524 | 71 | M | PCV | 1 | no | 3.1 |
| 1525 | 83 | M | PCV | 0 | no | 1.6 |
| 1526 | 85 | F | PCV | 0 | no | 2.6 |
| 1527 | 73 | F | PCV | 0 | yes | 4.2 |
| 1528 | 78 | M | PCV | 1 | no | 8.0 |
| 1529 | 65 | M | PCV | 0 | no | 4.5 |
| 1530 | 65 | M | PCV | 0 | yes | 3.1 |
| 1531 | 75 | M | PCV | 0 | no | 3.0 |
| 1532 | 81 | M | PCV | 0 | no | 5.8 |
| 1533 | 78 | M | PCV | 0 | no | 0.1 |
| 1534 | 86 | M | PCV | 1 | no | 19.7 |
| 1535 | 74 | M | PCV | 0 | no | 2.8 |
| 1536 | 65 | M | PCV | 1 | no | 0.6 |
| 1537 | 68 | F | PCV | 0 | no | 12.0 |
| 1538 | 78 | M | PCV | 1 | yes | 9.7 |
| 1539 | 86 | M | PCV | 1 | no | 4.4 |
| 1540 | 71 | M | PCV | 0 | no | 3.5 |
| 1541 | 71 | M | PCV | 0 | no | 0.7 |
| 1542 | 79 | F | PCV | 0 | no | 8.3 |
| 1543 | 77 | M | PCV | 0 | no | 12.4 |
| 1544 | 71 | M | PCV | 0 | no | 4.8 |
|  |  |  | PCV | 0 | no | 3.5 |
| 1545 | 70 | M | PCV | 1 | no | 10.3 |
| 1546 | 81 | M | PCV | 1 | no | 9.4 |
| 1547 | 68 | M | PCV | 2 | yes | 42.0 |
| 1548 | 75 | M | PCV | 0 | yes | 0.6 |
| 1549 | 70 | M | PCV | 1 | no | 2.1 |
| 1550 | 83 | F | PCV | 0 | no | 7.7 |
| 1551 | 58 | M | PCV | 0 | yes | 1.5 |
| 1552 | 65 | M | PCV | 0 | yes | 18.8 |
| 1553 | 79 | M | PCV | 2 | no | 9.3 |
| 1554 | 70 | M | PCV | 0 | no | 5.8 |
| 1555 | 62 | M | PCV | 0 | no | 0.9 |
| 1556 | 86 | M | PCV | 1 | yes | 3.0 |
| 1557 | 79 | M | PCV | 0 | no | 2.0 |
| 1558 | 60 | M | PCV | 1 | yes | 0.2 |
| 1559 | 80 | M | PCV | 1 | no | 3.8 |
| 1560 | 82 | M | PCV | 1 | yes | 10.2 |
| 1561 | 70 | M | PCV | 1 | yes | 14.7 |
| 1562 | 62 | M | PCV | 1 | yes | 4.8 |
| 1563 | 65 | F | PCV | 0 | yes | 15.3 |
| 1564 | 79 | M | PCV | 0 | no | 1.6 |
| 1565 | 77 | M | PCV | 0 | no | 0.4 |
| 1566 | 65 | M | PCV | 0 | no | 3.7 |
| 1567 | 66 | M | PCV | 1 | yes | 13.7 |
| 1568 | 75 | F | PCV | 0 | no | 5.3 |
| 1569 | 59 | M | PCV | 0 | no | 2.9 |
| 1570 | 82 | F | RAP | 1 | yes | 5.9 |
| 1571 | 89 | F | RAP | 0 | no | 0.9 |
| 1572 | 87 | F | RAP | 0 | no | 0.9 |
| 1573 | 91 | F | RAP | 0 | no | 1.3 |
| 1574 | 63 | F | RAP | 1 | yes | 1.6 |
| 1575 | 83 | F | RAP | 1 | yes | 3.6 |
| 1576 | 80 | M | RAP | 1 | yes | 10.5 |
|  |  |  | RAP | 1 | no | 7.0 |
| 1577 | 89 | F | RAP | 1 | no | 3.3 |
| 1578 | 79 | M | RAP | 1 | yes | 7.2 |
| 1579 | 77 | M | RAP | 1 | yes | 7.7 |
| 1580 | 77 | F | RAP | 1 | yes | 3.6 |
|  |  |  | RAP | 1 | yes | 4.6 |
| 1581 | 82 | F | RAP | 1 | no | 1.2 |
| 1582 | 64 | F | RAP | 0 | no | 1.9 |
| 1583 | 81 | M | RAP | 1 | yes | 7.4 |
|  |  |  | RAP | 1 | yes | 3.6 |
| 1584 | 86 | M | RAP | 0 | yes | 0.6 |
| 1585 | 88 | M | RAP | 1 | yes | 1.3 |
| 1586 | 83 | F | RAP | 1 | no | 3.3 |
| 1587 | 83 | M | RAP | 2 | no | 13.1 |
| 1588 | 80 | M | RAP | 1 | yes | 6.1 |
| 1589 | 74 | F | RAP | 1 | no | 1.0 |
| 1590 | 88 | F | RAP | 1 | no | 1.9 |
| 1591 | 76 | M | combined | 0 | no | NA |
| 1592 | 78 | M | combined | 0 | no | NA |
| 1593 | 78 | M | combined | 0 | yes | NA |
| 1594 | 76 | M | combined | 0 | no | NA |
| 1595 | 77 | F | combined | 0 | no | NA |
| 1596 | 83 | M | combined | 0 | no | NA |
| 1597 | 83 | F | combined | 0 | no | NA |
| 1598 | 81 | F | combined | 0 | no | NA |
| 1599 | 70 | M | combined | 0 | no | NA |
|  | 66 | M | combined | 1 | no | NA |
| 1600 | 66 |  | combined | 0 | yes | NA |

M, male; F, female; BCVA, best-corrected visual acuities; AMD, age-related macular degeneration; PED, pigment epithelial detachments; DA, Macular Photocoagulation Study disc areas; tAMD, typical AMD; PCV, polypoidal choroidal vasculopathy; RAP, retinal angiomatous proliferation; NA, not applicable.
