# Supplementary material for: Long-term characteristics of exudative age-related macular degeneration in Japanese patients
Source: PLoS One. 2021 Dec 14;16(12):e0261320. doi: 10.1371/journal.pone.0261320 (PMC8670714; doi:10.1371/journal.pone.0261320)
Supplement: S2 Table — (DOCX) [file pone.0261320.s002.docx]

Table S2: Raw data of CNV subtypes detected by fluorescein angiography in typical AMD

| **Phase I** |  |  |
| --- | --- | --- |
| patient No. | AMD Subtype | CNV Subtype |
| 1 | tAMD | predominantly |
| 2 | tAMD | occult |
| 3 | tAMD | minimally |
| 4 | tAMD | predominantly |
| 5 | tAMD | predominantly |
| 6 | tAMD | occult |
| 7 | tAMD | minimally |
| 8 | tAMD | predominantly |
| 9 | tAMD | predominantly |
| 10 | tAMD | predominantly |
| 11 | tAMD | minimally |
| 12 | tAMD | occult |
| 13 | tAMD | predominantly |
| 14 | tAMD | occult |
| 15 | tAMD | minimally |
| 16 | tAMD | minimally |
| 17 | tAMD | occult |
|  | tAMD | minimally |
| 18 | tAMD | occult |
| 19 | tAMD | occult |
|  | tAMD | occult |
| 20 | tAMD | predominantly |
| 21 | tAMD | predominantly |
|  | tAMD | predominantly |
| 22 | tAMD | occult |
| 23 | tAMD | occult |
| 24 | tAMD | minimally |
| 25 | tAMD | occult |
| 26 | tAMD | minimally |
| 27 | tAMD | occult |
| 28 | tAMD | predominantly |
| 29 | tAMD | minimally |
| 30 | tAMD | occult |
| 31 | tAMD | occult |
| 32 | tAMD | occult |
| 33 | tAMD | occult |
| 34 | tAMD | minimally |
| 35 | tAMD | minimally |
| 36 | tAMD | minimally |
| 37 | tAMD | minimally |
| 38 | tAMD | minimally |
| 39 | tAMD | minimally |
| 40 | tAMD | occult |
| 41 | tAMD | occult |
| 42 | tAMD | predominantly |
| 43 | tAMD | minimally |
| 44 | tAMD | occult |
| 45 | tAMD | minimally |
| 46 | tAMD | occult |
| 47 | tAMD | predominantly |
| 48 | tAMD | minimally |
| 49 | tAMD | minimally |
| 50 | tAMD | occult |
| 51 | tAMD | predominantly |
| 52 | tAMD | minimally |
| 53 | tAMD | minimally |
| 54 | tAMD | minimally |
|  | tAMD | predominantly |
| 55 | tAMD | occult |
| 56 | tAMD | minimally |
|  | tAMD | minimally |
| 57 | tAMD | occult |
| 58 | tAMD | minimally |
| 59 | tAMD | occult |
| 60 | tAMD | minimally |
| 61 | tAMD | occult |
| 62 | tAMD | predominantly |
| 63 | tAMD | predominantly |
| 64 | tAMD | occult |
| 65 | tAMD | minimally |
| 66 | tAMD | predominantly |
| 67 | tAMD | predominantly |
| 68 | tAMD | occult |
| 69 | tAMD | minimally |
| 70 | tAMD | occult |
| 71 | tAMD | minimally |
| 72 | tAMD | occult |
| 73 | tAMD | occult |
| 74 | tAMD | minimally |
| 75 | tAMD | occult |
| 76 | tAMD | minimally |
| 77 | tAMD | minimally |
| 78 | tAMD | predominantly |
| 79 | tAMD | occult |
| 80 | tAMD | minimally |
| 81 | tAMD | occult |
| 82 | tAMD | occult |
| 83 | tAMD | predominantly |
| 84 | tAMD | predominantly |
| 85 | tAMD | minimally |
| 86 | tAMD | occult |
| 87 | tAMD | minimally |
| 88 | tAMD | minimally |
| 89 | tAMD | predominantly |
|  | tAMD | occult |
| 90 | tAMD | minimally |
| 91 | tAMD | occult |
| 92 | tAMD | predominantly |
| 93 | tAMD | minimally |
| 94 | tAMD | occult |
| 95 | tAMD | predominantly |
| 96 | tAMD | occult |
| 97 | tAMD | minimally |
| 98 | tAMD | minimally |
| 99 | tAMD | minimally |
| 100 | tAMD | predominantly |
| 101 | tAMD | minimally |
| 102 | tAMD | occult |
| 274 | combined | occult |
| 275 | combined | occult |
| 276 | combined | occult |
| 277 | combined | minimally |
| 278 | combined | occult |
| 279 | combined | occult |
| 280 | combined | occult |
| 281 | combined | minimally |
| 282 | combined | occult |
| 283 | combined | minimally |
| 284 | combined | occult |
| 285 | combined | occult |
| 286 | combined | occult |
| 287 | combined | occult |
| 288 | combined | minimally |
| 289 | combined | predominantly |
| Phase II |  |  |
| patient No. | AMD Subtype | CNV Subtype |
| 290 | tAMD | occult |
| 291 | tAMD | occult |
| 292 | tAMD | predomi |
| 293 | tAMD | occult |
| 294 | tAMD | minimally |
| 295 | tAMD | occult |
| 296 | tAMD | minimally |
| 297 | tAMD | occult |
|  | tAMD | minimally |
| 298 | tAMD | occult |
| 299 | tAMD | occult |
| 300 | tAMD | predomi |
| 301 | tAMD | predomi |
| 302 | tAMD | predomi |
| 303 | tAMD | occult |
|  | tAMD | minimally |
| 304 | tAMD | occult |
|  | tAMD | minimally |
| 305 | tAMD | minimally |
| 306 | tAMD | occult |
| 307 | tAMD | minimally |
| 308 | tAMD | minimally |
| 309 | tAMD | minimally |
| 310 | tAMD | occult |
| 311 | tAMD | occult |
| 312 | tAMD | occult |
| 313 | tAMD | occult |
| 314 | tAMD | minimally |
| 315 | tAMD | minimally |
| 316 | tAMD | occult |
|  | tAMD | occult |
| 317 | tAMD | minimally |
| 318 | tAMD | occult |
| 319 | tAMD | occult |
| 320 | tAMD | occult |
| 321 | tAMD | occult |
| 322 | tAMD | occult |
| 323 | tAMD | occult |
| 324 | tAMD | predomi |
| 325 | tAMD | occult |
| 326 | tAMD | occult |
|  | tAMD | minimally |
| 327 | tAMD | minimally |
| 328 | tAMD | minimally |
| 329 | tAMD | minimally |
|  | tAMD | minimally |
| 330 | tAMD | minimally |
| 331 | tAMD | occult |
| 332 | tAMD | minimally |
| 333 | tAMD | predomi |
|  | tAMD | predomi |
| 334 | tAMD | predomi |
| 335 | tAMD | predomi |
| 336 | tAMD | predomi |
| 337 | tAMD | predomi |
| 338 | tAMD | minimally |
| 339 | tAMD | occult |
| 340 | tAMD | minimally |
| 341 | tAMD | occult |
| 342 | tAMD | minimally |
| 343 | tAMD | occult |
| 344 | tAMD | occult |
| 345 | tAMD | occult |
| 346 | tAMD | occult |
| 347 | tAMD | minimally |
| 348 | tAMD | minimally |
| 349 | tAMD | occult |
| 350 | tAMD | occult |
| 351 | tAMD | minimally |
| 352 | tAMD | occult |
|  | tAMD | occult |
| 353 | tAMD | minimally |
| 354 | tAMD | occult |
| 355 | tAMD | predominantly |
| 356 | tAMD | occult |
| 357 | tAMD | occult |
| 358 | tAMD | occult |
| 359 | tAMD | minimally |
| 360 | tAMD | predominantly |
| 361 | tAMD | occult |
| 362 | tAMD | occult |
| 363 | tAMD | minimally |
| 364 | tAMD | minimally |
| 365 | tAMD | occult |
| 366 | tAMD | occult |
| 367 | tAMD | occult |
| 368 | tAMD | occult |
| 369 | tAMD | occult |
| 370 | tAMD | minimally |
| 371 | tAMD | minimally |
| 372 | tAMD | minimally |
| 373 | tAMD | occult |
| 374 | tAMD | minimally |
| 375 | tAMD | minimally |
| 376 | tAMD | minimally |
| 377 | tAMD | occult |
| 378 | tAMD | minimally |
| 379 | tAMD | minimally |
| 380 | tAMD | occult |
| 381 | tAMD | minimally |
| 382 | tAMD | occult |
| 383 | tAMD | minimally |
| 384 | tAMD | minimally |
| 385 | tAMD | predominantly |
| 386 | tAMD | occult |
|  | tAMD | occult |
| 387 | tAMD | minimally |
| 388 | tAMD | occult |
| 389 | tAMD | minimally |
| 390 | tAMD | occult |
| 391 | tAMD | occult |
| 392 | tAMD | occult |
| 393 | tAMD | occult |
| 394 | tAMD | predominantly |
| 395 | tAMD | occult |
| 396 | tAMD | occult |
| 397 | tAMD | occult |
| 398 | tAMD | minimally |
| 399 | tAMD | minimally |
| 400 | tAMD | minimally |
| 401 | tAMD | predominantly |
| 402 | tAMD | occult |
| 403 | tAMD | occult |
| 404 | tAMD | occult |
| 548 | combined | occult |
| 549 | combined | occult |
| 550 | combined | occult |
| 551 | combined | occult |
| 552 | combined | occult |
| 553 | combined | minimally |
| 554 | combined | occult |
| 555 | combined | occult |
| 556 | combined | occult |
| 557 | combined | occult |
| 558 | combined | occult |
| 559 | combined | occult |
| 560 | combined | occult |
| 561 | combined | occult |
| Phase III |  |  |
| patient No. | AMD Subtype | CNV Subtype |
| 562 | tAMD | occult |
| 563 | tAMD | occult |
| 564 | tAMD | occult |
| 565 | tAMD | minimally |
| 566 | tAMD | minimally |
| 567 | tAMD | occult |
| 568 | tAMD | minimally |
| 569 | tAMD | occult |
| 570 | tAMD | minimally |
| 571 | tAMD | minimally |
| 572 | tAMD | occult |
| 573 | tAMD | minimally |
| 574 | tAMD | minimally |
| 575 | tAMD | occult |
| 576 | tAMD | occult |
| 577 | tAMD | occult |
| 578 | tAMD | occult |
| 579 | tAMD | minimally |
| 580 | tAMD | occult |
| 581 | tAMD | Predominantly |
| 582 | tAMD | minimally |
| 583 | tAMD | occult |
| 584 | tAMD | minimally |
| 585 | tAMD | minimally |
| 586 | tAMD | minimally |
| 587 | tAMD | occult |
| 588 | tAMD | occult |
| 589 | tAMD | occult |
| 590 | tAMD | minimally |
| 591 | tAMD | predominantly |
| 592 | tAMD | occult |
| 593 | tAMD | minimally |
| 594 | tAMD | occult |
|  | tAMD | occult |
| 595 | tAMD | minimally |
| 596 | tAMD | minimally |
| 597 | tAMD | occult |
| 598 | tAMD | minimally |
| 599 | tAMD | occult |
| 600 | tAMD | occult |
| 601 | tAMD | predominantly |
| 602 | tAMD | predominantly |
| 603 | tAMD | minimally |
| 604 | tAMD | occult |
| 605 | tAMD | occult |
| 606 | tAMD | minimally |
| 607 | tAMD | minimally |
| 608 | tAMD | minimally |
| 609 | tAMD | minimally |
| 610 | tAMD | occult |
|  | tAMD | occult |
| 611 | tAMD | predominantly |
| 612 | tAMD | predominantly |
| 613 | tAMD | minimally |
| 614 | tAMD | occult |
| 615 | tAMD | predominantly |
| 616 | tAMD | minimally |
| 617 | tAMD | occult |
| 618 | tAMD | occult |
| 619 | tAMD | minimally |
| 620 | tAMD | predominantly |
| 621 | tAMD | occult |
| 622 | tAMD | predominantly |
| 623 | tAMD | minimally |
| 624 | tAMD | occult |
|  | tAMD | occult |
| 625 | tAMD | predominantly |
|  | tAMD | occult |
| 626 | tAMD | occult |
|  | tAMD | occult |
| 627 | tAMD | occult |
| 628 | tAMD | minimally |
| 629 | tAMD | occult |
| 630 | tAMD | occult |
| 631 | tAMD | minimally |
| 632 | tAMD | minimally |
| 633 | tAMD | occult |
| 634 | tAMD | predominantly |
| 635 | tAMD | minimally |
| 636 | tAMD | classic |
| 637 | tAMD | occult |
|  | tAMD | occult |
| 638 | tAMD | occult |
| 639 | tAMD | occult |
| 640 | tAMD | occult |
| 641 | tAMD | occult |
| 642 | tAMD | occult |
| 643 | tAMD | minimally |
| 644 | tAMD | minimally |
| 645 | tAMD | occult |
| 646 | tAMD | occult |
| 647 | tAMD | occult |
| 648 | tAMD | occult |
| 649 | tAMD | occult |
| 650 | tAMD | minimally |
| 651 | tAMD | occult |
| 652 | tAMD | occult |
| 653 | tAMD | occult |
|  | tAMD | occult |
| 654 | tAMD | occult |
| 655 | tAMD | minimally |
| 656 | tAMD | occult |
| 657 | tAMD | minimally |
| 658 | tAMD | occult |
| 659 | tAMD | occult |
| 660 | tAMD | occult |
|  | tAMD | occult |
| 661 | tAMD | minimally |
| 662 | tAMD | predominantly |
| 663 | tAMD | occult |
| 664 | tAMD | minimally |
| 665 | tAMD | occult |
| 666 | tAMD | occult |
| 667 | tAMD | occult |
| 668 | tAMD | predominantly |
| 669 | tAMD | occult |
| 670 | tAMD | minimally |
| 671 | tAMD | occult |
| 672 | tAMD | predominantly |
| 673 | tAMD | occult |
| 674 | tAMD | occult |
| 675 | tAMD | occult→type2 |
| 676 | tAMD | minimally |
| 677 | tAMD | occult |
| 678 | tAMD | minimally |
| 679 | tAMD | minimally |
| 680 | tAMD | occult |
|  | tAMD | occult |
| 681 | tAMD | minimally |
| 682 | tAMD | predominantly |
| 683 | tAMD | occult |
| 684 | tAMD | occult |
| 685 | tAMD | occult |
| 686 | tAMD | occult |
| 687 | tAMD | occult |
| 688 | tAMD | occult |
| 689 | tAMD | occult |
|  | tAMD | occult |
| 690 | tAMD | predominantly |
| 691 | tAMD | occult |
|  | tAMD | occult |
| 692 | tAMD | occult |
| 693 | tAMD | minimally |
| 694 | tAMD | occult |
| 695 | tAMD | minimally |
| 696 | tAMD | occult |
| 697 | tAMD | occult |
| 698 | tAMD | minimally |
| 699 | tAMD | predominantly |
| 700 | tAMD | occult |
|  | tAMD | occult |
| 701 | tAMD | occult |
| 702 | tAMD | predominantly |
| 703 | tAMD | occult |
| 704 | tAMD | occult |
| 705 | tAMD | occult |
| 706 | tAMD | occult |
| 707 | tAMD | occult |
| 708 | tAMD | minimally |
| 709 | tAMD | predominantly |
| 710 | tAMD | predominantly |
| 711 | tAMD | occult |
| 712 | tAMD | minimally |
| 713 | tAMD | occult |
|  | tAMD | occult |
| 714 | tAMD | occult |
| 715 | tAMD | predominantly |
| 716 | tAMD | minimally |
| 717 | tAMD | occult |
| 718 | tAMD | occult |
|  | tAMD | occult |
| 719 | tAMD | predominantly |
| 720 | tAMD | occult |
|  | tAMD | occult |
| 721 | tAMD | predominantly |
| 722 | tAMD | occult |
| 723 | tAMD | predominantly |
| 724 | tAMD | minimally |
| 725 | tAMD | occult |
| 726 | tAMD | minimally |
|  | tAMD | minimally |
| 727 | tAMD | predominantly |
| 728 | tAMD | minimally |
| 729 | tAMD | occult |
| 730 | tAMD | minimally |
| 731 | tAMD | occult |
| 732 | tAMD | predominantly |
| 733 | tAMD | occult |
| 879 | combined | minimally |
| 880 | combined | occult |
| 881 | combined | occult |
| 882 | combined | occult |
| 883 | combined | occult |
| 884 | combined | occult |
| 885 | combined | minimally |
| Phase IV |  |  |
| patient No. | AMD Subtype | CNV Subtype |
| 886 | tAMD | occult |
|  | tAMD | occult |
| 887 | tAMD | occult |
| 888 | tAMD | occult |
| 889 | tAMD | minimally |
| 890 | tAMD | occult |
| 891 | tAMD | occult |
| 892 | tAMD | occult |
| 893 | tAMD | occult |
| 894 | tAMD | occult |
| 895 | tAMD | predominantly |
| 896 | tAMD | occult |
| 897 | tAMD | occult |
| 898 | tAMD | occult |
| 899 | tAMD | occult |
| 900 | tAMD | predominantly |
| 901 | tAMD | predominantly |
| 902 | tAMD | occult |
| 903 | tAMD | minimally |
| 904 | tAMD | occult |
| 905 | tAMD | occult |
| 906 | tAMD | minimally |
| 907 | tAMD | minimally |
| 908 | tAMD | minimally |
| 909 | tAMD | occult |
| 910 | tAMD | predominantly |
| 911 | tAMD | occult |
| 912 | tAMD | minimally |
| 913 | tAMD | minimally |
| 914 | tAMD | occult |
| 915 | tAMD | minimally |
| 916 | tAMD | minimally |
| 917 | tAMD | occult |
| 918 | tAMD | predominantly |
| 919 | tAMD | occult |
| 920 | tAMD | minimally |
| 921 | tAMD | occult |
| 922 | tAMD | occult |
| 923 | tAMD | minimally |
| 924 | tAMD | minimally |
| 925 | tAMD | minimally |
| 926 | tAMD | occult |
| 927 | tAMD | minimally |
| 928 | tAMD | minimally |
| 929 | tAMD | occult |
| 930 | tAMD | occult |
| 931 | tAMD | occult |
| 932 | tAMD | occult |
| 933 | tAMD | predominantly |
| 934 | tAMD | minimally |
| 935 | tAMD | occult |
| 936 | tAMD | minimally |
| 937 | tAMD | minimally |
| 938 | tAMD | occult |
| 939 | tAMD | predominantly |
| 940 | tAMD | occult |
| 941 | tAMD | predominantly |
| 942 | tAMD | predominantly |
| 943 | tAMD | minimally |
| 944 | tAMD | predominantly |
| 945 | tAMD | predominantly |
| 946 | tAMD | occult |
| 947 | tAMD | predominantly |
| 948 | tAMD | minimally |
| 949 | tAMD | occult |
| 950 | tAMD | predominantly |
| 951 | tAMD | minimally |
| 952 | tAMD | occult |
| 953 | tAMD | occult |
| 954 | tAMD | occult |
| 955 | tAMD | minimally |
| 956 | tAMD | occult |
| 957 | tAMD | occult |
| 958 | tAMD | occult |
| 959 | tAMD | occult |
| 960 | tAMD | minimally |
| 961 | tAMD | minimally |
| 962 | tAMD | occult |
| 963 | tAMD | occult |
| 964 | tAMD | occult |
| 965 | tAMD | predominantly |
| 966 | tAMD | occult |
| 967 | tAMD | occult |
| 968 | tAMD | predominantly |
| 969 | tAMD | occult |
| 970 | tAMD | occult |
| 971 | tAMD | minimally |
| 972 | tAMD | predominantly |
| 973 | tAMD | minimally |
| 974 | tAMD | occult |
| 975 | tAMD | occult |
| 976 | tAMD | occult |
| 977 | tAMD | occult |
| 978 | tAMD | occult |
| 979 | tAMD | predominantly |
| 980 | tAMD | minimally |
| 981 | tAMD | occult |
| 982 | tAMD | minimally |
| 983 | tAMD | occult |
| 984 | tAMD | occult |
| 985 | tAMD | minimally |
| 986 | tAMD | occult |
| 987 | tAMD | minimally |
| 988 | tAMD | occult |
| 989 | tAMD | minimally |
| 990 | tAMD | occult |
| 991 | tAMD | occult |
| 992 | tAMD | occult |
| 993 | tAMD | predominantly |
| 994 | tAMD | occult |
| 995 | tAMD | occult |
| 996 | tAMD | minimally |
| 997 | tAMD | minimally |
| 998 | tAMD | predominantly |
| 999 | tAMD | minimally |
| 1000 | tAMD | occult |
| 1001 | tAMD | occult |
| 1002 | tAMD | predominantly |
| 1003 | tAMD | occult |
| 1004 | tAMD | occult |
| 1005 | tAMD | occult |
| 1006 | tAMD | predominantly |
| 1007 | tAMD | occult |
| 1008 | tAMD | occult |
| 1009 | tAMD | minimally |
| 1010 | tAMD | occult |
| 1011 | tAMD | occult |
| 1012 | tAMD | minimally |
| 1013 | tAMD | occult |
| 1014 | tAMD | minimally |
| 1015 | tAMD | occult |
| 1016 | tAMD | occult |
| 1017 | tAMD | minimally |
| 1018 | tAMD | occult |
| 1019 | tAMD | minimally |
| 1020 | tAMD | minimally |
| 1021 | tAMD | occult |
| 1022 | tAMD | occult |
| 1023 | tAMD | occult |
| 1024 | tAMD | occult |
| 1025 | tAMD | predominantly |
|  | tAMD | predominantly |
| 1026 | tAMD | occult |
| 1027 | tAMD | occult |
| 1028 | tAMD | occult |
| 1029 | tAMD | occult |
| 1030 | tAMD | occult |
| 1031 | tAMD | occult |
| 1032 | tAMD | minimally |
| 1033 | tAMD | minimally |
| 1034 | tAMD | occult |
| 1035 | tAMD | minimally |
| 1036 | tAMD | predominantly |
| 1037 | tAMD | occult |
| 1038 | tAMD | occult |
| 1212 | combined | minimally |
| 1213 | combined | occult |
| Phase V |  |  |
| patient No. | AMD Subtype | CNV Subtype |
| 1214 | tAMD | occult |
| 1215 | tAMD | predominantly |
| 1216 | tAMD | occult |
| 1217 | tAMD | occult |
| 1218 | tAMD | minimally |
| 1219 | tAMD | occult |
| 1220 | tAMD | occult |
| 1221 | tAMD | occult |
| 1222 | tAMD | minimally |
| 1223 | tAMD | occult |
| 1224 | tAMD | predominantly |
| 1225 | tAMD | minimally |
| 1226 | tAMD | occult |
| 1227 | tAMD | minimally |
| 1228 | tAMD | occult |
| 1229 | tAMD | minimally |
| 1230 | tAMD | predominantly |
| 1231 | tAMD | occult |
| 1232 | tAMD | minimally |
| 1233 | tAMD | predominantly |
| 1234 | tAMD | occult |
| 1235 | tAMD | occult |
| 1236 | tAMD | occult |
| 1237 | tAMD | occult |
| 1238 | tAMD | occult |
| 1239 | tAMD | predominantly |
| 1240 | tAMD | predominantly |
| 1241 | tAMD | minimally |
| 1242 | tAMD | occult |
| 1243 | tAMD | occult |
| 1244 | tAMD | occult |
| 1245 | tAMD | occult |
| 1246 | tAMD | occult |
| 1247 | tAMD | occult |
| 1248 | tAMD | occult |
| 1249 | tAMD | minimally |
| 1250 | tAMD | occult |
| 1251 | tAMD | minimally |
| 1252 | tAMD | occult |
| 1253 | tAMD | occult |
|  | tAMD | occult |
| 1254 | tAMD | occult |
| 1255 | tAMD | occult |
| 1256 | tAMD | minimally |
| 1257 | tAMD | occult |
| 1258 | tAMD | occult |
| 1259 | tAMD | minimally |
| 1260 | tAMD | occult |
| 1261 | tAMD | minimally |
| 1262 | tAMD | occult |
| 1263 | tAMD | occult |
| 1264 | tAMD | predominantly |
| 1265 | tAMD | minimally |
| 1266 | tAMD | occult |
| 1267 | tAMD | occult |
| 1268 | tAMD | occult |
| 1269 | tAMD | occult |
| 1270 | tAMD | occult |
| 1271 | tAMD | occult |
| 1272 | tAMD | minimally |
| 1273 | tAMD | occult |
| 1274 | tAMD | occult |
| 1275 | tAMD | occult |
| 1276 | tAMD | occult |
| 1277 | tAMD | occult |
| 1278 | tAMD | predominantly |
| 1279 | tAMD | occult |
| 1280 | tAMD | occult |
| 1281 | tAMD | occult |
| 1282 | tAMD | occult |
| 1283 | tAMD | minimally |
| 1284 | tAMD | occult |
| 1285 | tAMD | predominantly |
| 1286 | tAMD | predominantly |
| 1287 | tAMD | occult |
| 1288 | tAMD | predominantly |
| 1289 | tAMD | minimally |
| 1290 | tAMD | minimally |
| 1291 | tAMD | occult |
|  | tAMD | occult |
| 1292 | tAMD | occult |
| 1293 | tAMD | occult |
| 1294 | tAMD | predominantly |
| 1295 | tAMD | minimally |
| 1296 | tAMD | minimally |
| 1297 | tAMD | occult |
| 1298 | tAMD | occult |
| 1299 | tAMD | occult |
| 1300 | tAMD | occult |
| 1301 | tAMD | occult |
| 1302 | tAMD | minimally |
| 1303 | tAMD | occult |
| 1304 | tAMD | occult |
| 1305 | tAMD | occult |
| 1306 | tAMD | minimally |
| 1307 | tAMD | minimally |
| 1308 | tAMD | minimally |
| 1309 | tAMD | occult |
| 1310 | tAMD | minimally |
| 1311 | tAMD | occult |
| 1312 | tAMD | occult |
| 1313 | tAMD | predominantly |
| 1314 | tAMD | minimally |
| 1315 | tAMD | minimally |
| 1316 | tAMD | occult |
| 1317 | tAMD | predominantly |
| 1318 | tAMD | minimally |
| 1319 | tAMD | minimally |
| 1320 | tAMD | occult |
| 1321 | tAMD | occult |
| 1322 | tAMD | occult |
| 1323 | tAMD | occult |
| 1324 | tAMD | occult |
| 1325 | tAMD | occult |
| 1326 | tAMD | occult |
| 1327 | tAMD | occult |
| 1328 | tAMD | occult |
| 1329 | tAMD | minimally |
| 1330 | tAMD | occult |
| 1331 | tAMD | occult |
| 1332 | tAMD | occult |
| 1333 | tAMD | occult |
| 1334 | tAMD | occult |
| 1335 | tAMD | occult |
| 1336 | tAMD | occult |
| 1337 | tAMD | predominantly |
| 1338 | tAMD | occult |
| 1339 | tAMD | occult |
|  | tAMD | occult |
| 1340 | tAMD | occult |
| 1341 | tAMD | predominantly |
| 1342 | tAMD | predominantly |
| 1343 | tAMD | occult |
| 1344 | tAMD | occult |
| 1345 | tAMD | minimally |
| 1346 | tAMD | occult |
| 1347 | tAMD | occult |
| 1348 | tAMD | occult |
| 1349 | tAMD | minimally |
| 1350 | tAMD | predominantly |
| 1351 | tAMD | minimally |
| 1352 | tAMD | occult |
| 1353 | tAMD | predominantly |
| 1354 | tAMD | minimally |
| 1355 | tAMD | occult |
| 1356 | tAMD | occult |
| 1357 | tAMD | predominantly |
| 1358 | tAMD | occult |
| 1359 | tAMD | occult |
| 1360 | tAMD | occult |
| 1361 | tAMD | occult |
| 1362 | tAMD | predominantly |
| 1363 | tAMD | occult |
| 1364 | tAMD | occult |
| 1365 | tAMD | minimally |
| 1366 | tAMD | minimally |
| 1367 | tAMD | minimally |
| 1368 | tAMD | minimally |
| 1369 | tAMD | occult |
| 1370 | tAMD | predominantly |
| 1371 | tAMD | occult |
|  | tAMD | occult |
| 1372 | tAMD | occult |
| 1373 | tAMD | predominantly |
| 1374 | tAMD | minimally |
| 1375 | tAMD | predominantly |
| 1376 | tAMD | occult |
| 1377 | tAMD | predominantly |
| 1378 | tAMD | occult |
| 1379 | tAMD | minimally |
| 1380 | tAMD | occult |
| 1381 | tAMD | occult |
| 1382 | tAMD | occult |
| 1383 | tAMD | occult |
| 1384 | tAMD | occult |
| 1385 | tAMD | occult |
| 1386 | tAMD | predominantly |
| 1387 | tAMD | occult |
| 1388 | tAMD | occult |
| 1389 | tAMD | minimally |
| 1390 | tAMD | occult |
| 1391 | tAMD | minimally |
| 1591 | combined | occult |
| 1592 | combined | occult |
| 1593 | combined | occult |
| 1594 | combined | occult |
| 1595 | combined | occult |
| 1596 | combined | occult |
| 1597 | combined | occult |
| 1598 | combined | occult |
| 1599 | combined | occult |
| 1600 | combined | occult |

AMD, age-related macular degeneration; CNV, choroidal neovascularization; tAMD, typical AMD.
